# Exploration of linear and third-order nonlinear optical properties for donor–π-linker–acceptor chromophores derived from ATT-2 based non-fullerene molecule†

**DOI:** 10.1039/d3ra04580c

**Published:** 2023-11-01

**Authors:** Muhammad Sagir, Kalsoom Mushtaq, Muhammad Khalid, Mashal Khan, Muhammad Bilal Tahir, Ataualpa A. C. Braga

**Affiliations:** a Institute of Chemical and Environmental Engineering, Khwaja Fareed University of Engineering & Information Technology Rahim Yar Khan 64200 Pakistan; b Institute of Chemistry, Khwaja Fareed University of Engineering & Information Technology Rahim Yar Khan 64200 Pakistan; c Centre for Theoretical and Computational Research, Khwaja Fareed University of Engineering & Information Technology Rahim Yar Khan 64200 Pakistan; d Institute of Physics, Khwaja Fareed University of Engineering & Information Technology Rahim Yar Khan 64200 Pakistan; e Departamento de Química Fundamental, Instituto de Química, Universidade de Saõ Paulo Av. Prof. Lineu Prestes, 748 Sao Paulo 05508-000 Brazil khalid@iq.usp.br

## Abstract

In the current study, seven non-fullerene compounds abbreviated as ATTD2–ATTD8 were designed through structural tailoring and their nonlinear optical (NLO) properties were reported. The objective of this study was to explore the potential for newly configured D–π–A type non-fullerene-based compounds. Quantum chemical methods were adopted and revealed the molecules as highly efficient materials with favorable NLO characteristics for use in optoelectronic devices. The M06 functional along with the 6-311G(d,p) basis set in chloroform solvent were utilized for the natural bonding orbital (NBO) analysis, absorption spectra and computational assessments of frontier molecular orbitals (FMOs), global reactivity descriptors (GRPs), transition density matrix (TDM) and nonlinear optical properties (NLO) for ATTR1 and ATTD2–ATTD8. The HOMO–LUMO energy gap was significantly reduced in all the designed moieties compared to the reference compound in the following decreasing order: ATTR1 > ATTD8 > ATTD4 > ATTD5 > ATTD2 > ATTD7 > ATTD6 > ATTD3. All of the designed molecules (ATTD2–ATTD8) showed good NLO response. Global reactivity parameters were found to be closely associated with the band gap between the HOMO and LUMO orbitals, and the compound with the smallest energy gap, ATTD3, exhibited a lower hardness value of 1.754 eV and higher softness value of 0.570 eV with outstanding NLO response. For the reference compound and ATTD2–ATTD8 derivatives, attributes like dipole moment (*μ*_tot_), average polarizability 〈*α*〉, first hyperpolarizability (*β*_tot_), and second hyperpolarizability *γ*_tot_ were calculated. Out of all the derivatives, ATTD3 revealed the highest amplitude with a *β*_tot_ of 8.23 × 10^−27^ esu, which was consistent with the reduced band gap (1.754 eV) and suggested it was the best possibility for NLO materials in the future.

## Abstract 2

In the current study, seven non-fullerene compounds abbreviated as ATTD2–ATTD8 were designed through structural tailoring and their nonlinear optical (NLO) properties were reported.

## Graphical abstract

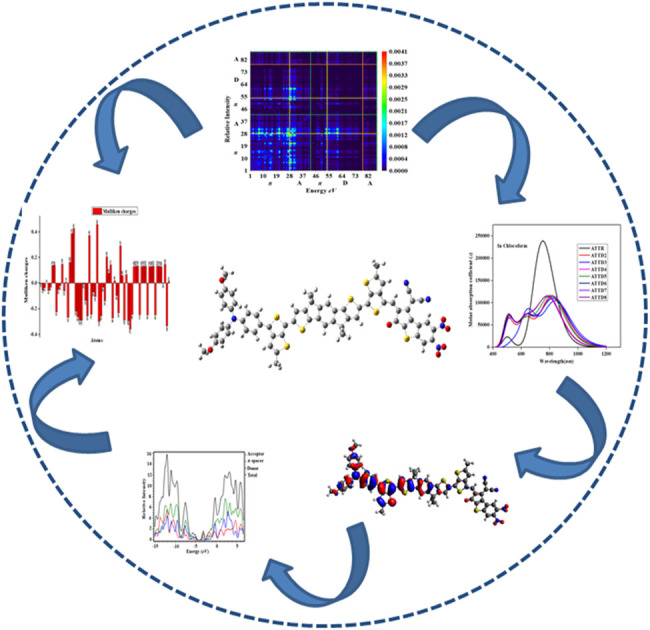

## Introduction

In recent decades, the significance of nonlinear optical (NLO) materials has grown within the fields of photonics and optoelectronics.^1^ Ongoing investigations have shown the importance of promising nonlinear (NLO) materials owing to their distinctive optical properties^2^ and these materials have been used in a variety of fields, such as solid-state physics, biophysics, material science and chemical dynamics.^3^ Additionally, they have found applications in the telecommunication industry^4^ and optical devices.^5^ Studies of first, second and third-order NLO properties are included in the field of nonlinear optics. The manufacturing of NLO materials is now a focus of advanced research, both theoretically and experimentally.^6,7^ Organic NLO materials exhibit attractive properties, including NLO susceptibilities and solubility that can be improved through structural modifications. To understand the relationship between the various parts of the studied compound, it is essential to understand its molecular structure.^8^ Organic materials are of great interest for their nonlinear optical response in various kinds of optical applications because of their unique molecular architectures, which are attributed to the existence of numerous functional groups and the accessibility of a wide range of synthetic techniques. They are extremely versatile due to their distinctive structural characteristics, which have led to their widespread use in many optical applications,^9^ including frequency doubling and the formation of terahertz (THz) waves.^10^

Overall, organic materials are considered robust materials for NLO applications due to their diverse molecular structures and ability to be tailored for specific optical functions. It is possible to obtain first, second and third-order nonlinear polarizabilities by using non-centrosymmetric π-spacer compounds to improve the density of charge transfer between the donor and acceptor.^9–11^

The magnitude of the HOMO–LUMO band gap plays a significant role in the determination of the electrical and structural properties of organic compounds as it significantly influences the extent of charge transfer that takes place within them.^12–14^ The molecular arrangements can be altered by modifying the D–π–A framework within the system, which in turn affects the intramolecular charge transfer (ICT) properties of the compound. The NLO study reveals that first hyper-polarizability (*β*_tot_) is associated with the ICT process occurring from the donor *via* the π-conjugated spacers to the acceptor moieties, indicating a correlation between ICT and the NLO characteristics of the compound.^15^ It has been reported that fullerene and its conjugated derivatives are promising candidates among various types of delocalized electronic conjugated molecules due to their nonlinear optical NLO performance.^16–19^

Fullerene-based compounds have some drawbacks that can limit their performance in certain applications. One such limitation is their low absorption in the visible range, which means they may not be as effective in certain types of photovoltaic devices that rely on visible light. Additionally, these compounds may suffer from poor stability, which can impact their long-term durability and reliability.^20,21^ In recent years, there has been a growing interest in non-fullerene (NF) based compounds due to their ability to adopt diverse chemical structures, possess a broad range of electron affinities, exhibit tunable energy levels and have a simple synthesis process. This has made them attractive candidates for a wide range of applications in areas such as organic photovoltaics and organic field-effect transistors.^22,23^

Advantages of NF-based organic acceptors (NFAs), which have taken the place of traditional fullerene acceptors, are (i) light absorption, (ii) a diversity of D–A blends, (iii) a greater dipole moment and (iv) balanced charge transfer.^24^ These advanced features of NFAs have led to a significant increase in their efficiency in the recent years. This is a result of their π-conjugated structure, which promotes effective charge transfer at the donor–acceptor interface and allows for effective electronic delocalization.^25^

In the realm of materials science and molecular design, the pursuit of promising nonlinear optical (NLO) properties has become a focal point for groundbreaking research. NLO properties refer to a material's ability to exhibit nonlinear responses when exposed to intense electromagnetic fields, a phenomenon crucial for various technological applications such as laser systems and photonic devices. The theoretical design of organic compounds with enhanced NLO properties is a burgeoning field driven by the quest for novel materials that can surpass the limitations of existing ones. By tailoring the electronic and structural characteristics of organic molecules through computational methods, researchers aim to manipulate their hyperpolarizabilities and molecular nonlinearities, thus paving the way for the development of efficient and sustainable NLO materials. This work not only holds great promise for advancing the field of optics but also contributes to the broader landscape of materials science, offering innovative solutions to meet the demands of emerging technologies.

Density functional theory (DFT) studies, involving information like natural bond orbitals (NBOs), frontier molecular orbitals (FMOs) and ultraviolet-visible (UV-Vis) spectra, were carried out for the title compounds to understand their NLO behavior. Synthesized compound (ATT-2) has been reported in the literature^26^ and, according to our best knowledge, its NLO study has not been performed yet. ATTR1 is a non-fullerene based compound with the IUPAC name 2,2'-((2*Z*,2′*Z*)-(((4,4,9,9-tetramethyl-4,9-dihydro-*s*-indaceno[1,2-*b*:5,6-*b*′]dithiophene-2,7-diyl)bis(2-methylthieno[3,4-*b*]thiophene-6,4-diyl))bis(methaneylylidene))bis(3-oxo-2,3-dihydro-1*H*-indene-2,1-diylidene))dimalononitrile and is comprised of two acceptors and a π-spacer. In this study, we designed a series of seven D–π–A type NF-based chromophores (ATTD2–ATTD8) in order to obtain significant NLO materials. The study focused on tailoring the acceptor (A) groups while keeping the donor (D) and π-spacers constant. To determine the efficiency of the engineered molecules as effective NLO materials, multiple variables for all designed compounds (ATTD2–ATTD8) and the reference molecule (ATTR1) were calculated. These NLO-based findings could provide some insight for the development of novel organic entities with D–π–A architecture which are considered to be fullerene-free. The present research could also help researchers and experimentalists to develop potential NLO materials that exceed present expectations.

## Computational procedure

In this study, density functional theory (DFT) and time-dependent density functional theory (TD-DFT) were used to determine the absorption spectra, natural bond orbitals (NBOs), electronic characteristics and *γ*_tot_ findings for the reference compound and its derivatives. All computations were done using the Gaussian 09 program.^27^ The structural optimization of the given moieties was accomplished in chloroform using the M06 level of theory^28^ and the 6-311G(d,p) basis set.^29^ Avogadro software^30^ was utilized to produce FMO diagrams, displaying the highest occupied molecular orbitals (HOMOs) and lowest unoccupied molecular orbitals (LUMOs) of molecules. The visualization of FMOs provides an insight into the electronic properties and predicted reactivity of the molecules. Using Gaussum^31^ and Origin 8.5 (ref. 32) software, the UV-Vis spectral analysis was carried out at the mentioned level utilizing the TD-DFT approach. The input files for the computations were created using Gauss View 5.0 (ref. 33) and the remaining data were evaluated using Chemcraft 1.6 (ref. 34) and Multiwfn 3.7.^35^ Gaussian output files provide three linear polarizability tensors (*α*_*xx*_, *α*_*yy*_, *α*_*zz*_) and ten hyper-polarizability tensors (*β*_*xxx*_, *β*_*xyy*_, *β*_*xzz*_, *β*_*yyy*_, *β*_*xxy*_, *β*_*yzz*_, *β*_*zzz*_, *β*_*xxz*_, *β*_*yyz*_, *β*_*xyz*_) along the *x*, *y* and *z* directions, respectively.

Dipole moments were calculated using eqn (1).^36^

1 *μ* = (*μ*_*x*_^2^ + *μ*_*y*_^2^ + *μ*_*z*_^2^)^1/2^

Average polarizability was determined with eqn (2).^37^

2 〈*α*〉 = (*a*_*xx*_ + *a*_*yy*_ + *a*_*zz*_)^1/3^

The magnitude of total first hyper-polarizability (*β*_tot_)^38^ was measured *via*eqn (3),

3 *β*_tot_ = (*β*_*x*_^2^ + *β*_*y*_^2^ + *β*_*z*_^2^)^1/2^

where *β*_*x*_ = *β*_*xxx*_ + *β*_*xyy*_ + *β*_*xzz*_, *β*_*y*_ = *β*_*yxx*_ + *β*_*yyy*_ + *β*_*yzz*_ and *β*_*z*_ = *β*_*zxx*_ + *β*_*zyy*_ + *β*_*zzz*_.

The second hyper-polarizability *γ*_tot_ was computed utilizing eqn (4),^39^4
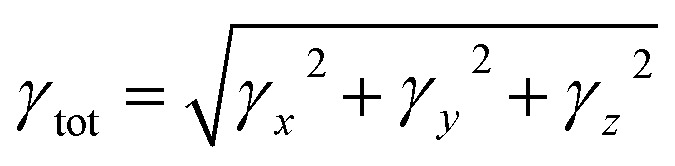
where 
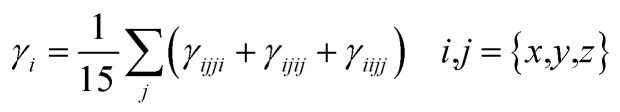
.

## Results and discussion

**Fig. 1:**
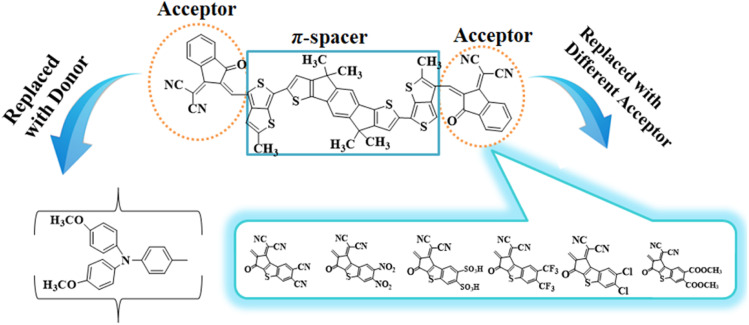
Graphical representations of ATTR1 and ATTD2–ATTD8 derivatives.

**Fig. 2:**
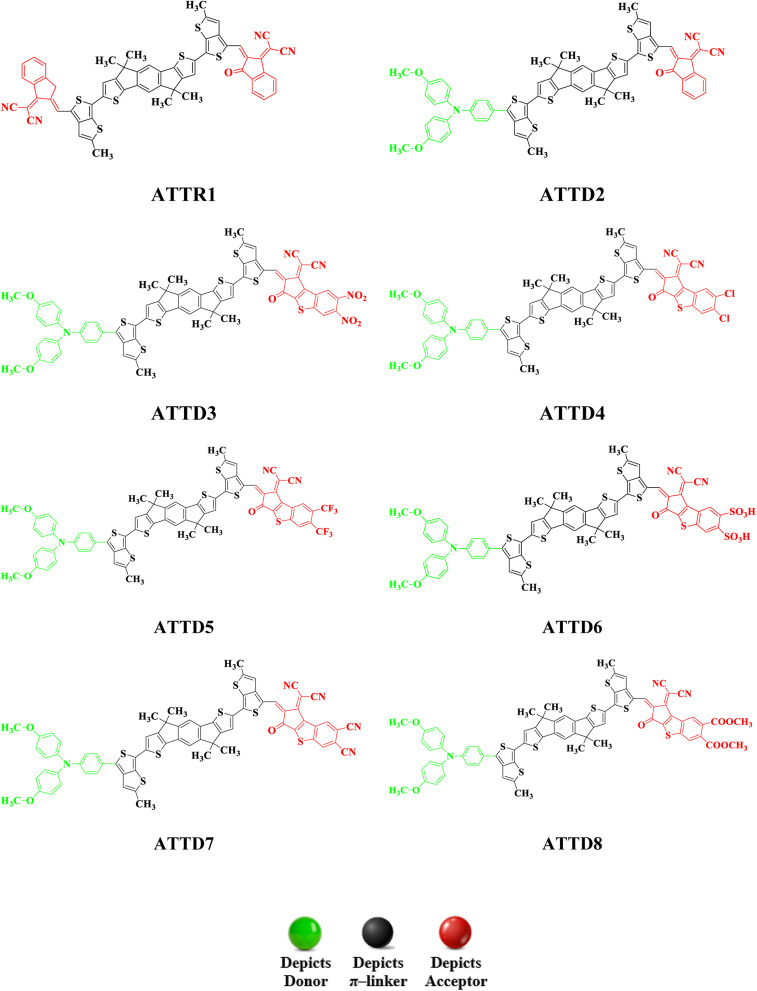
Structures of reference (ATTR1) and designed compounds (ATTD2–ATTD8).

To explore the NLO characteristics of the non-fullerene chromophores, *i.e.*, ATTR1 and its derivatives (ATTD2–ATTD8), a quantum chemical investigation was conducted. Firstly, the parent compound (ATT-2) was modified into the reference compound (ATTR1) by side-chain modification, as shown in Fig. S6.† To avoid computational cost, we replaced bulky groups such as octyl acetate and *n*-hexyl with simple methyl groups. The first derivative (ATTD2) was manufactured by replacing one terminal acceptor unit of ATTR1 (A–π–A) with a donor moiety (IUPAC name 4-methoxy-*N*-(4-methoxyphenyl)-*N*-phenylaniline) and the other terminal acceptor unit with another strong acceptor moiety in order to establish a strong push–pull scheme based on D–π–A architecture. In rest of the derivatives (ATTD3–ATTD8), the donor species is the same, while the acceptor terminal is modified with various strong acceptors. Fig. 1 depicts the structures of the series of acceptors which were utilized for molecular modelling. Our literature survey revealed that acceptor species are important in modifying the energy band gap (*E*_LUMO_ − *E*_HOMO_) and maximum wavelength (*λ*_max_) of organic chromophores. Therefore, we designed seven derivatives abbreviated as ATTD2–ATTD8 from the reference molecule. Fig. 2 shows the title compounds designed by combining the donors, π-linkers and acceptor groups in order to obtain efficient non-fullerene based NLO materials. The IUPAC names and the abbreviations of the investigated moieties are given in the Table S38.† The NLO characteristics of ATTR1 and its associated derivatives (ATTD2–ATTD8) have not been reported yet. Therefore, our research is unique in its nature and supports the fact that delocalized π-electron structure systems such as NFAs exhibit good NLO characteristics and could be used as future candidates by experimentalists in research laboratories in various fields. The Cartesian coordinates of ATTR1 and the designed derivatives (ATTD2–ATTD8) are presented in Tables S1–S8.† The optimized framework of the studied compounds is displayed in Fig. S1.†

### Frontier molecular orbitals (FMOs) analysis

Frontier molecular orbitals analysis is a powerful method for understanding the electronic properties of organic compounds, allowing for predictions of their reactivity, stability and numerous electronic transitions.^40^ It is an important variable to determine the charge transfer probability among the chromophores.^41^ The various interactions and reactions between molecules in a conjugated framework are controlled by the highest occupied molecular orbital (HOMO) and lowest unoccupied molecular orbital (LUMO).^42–44^ The transfer of electrons from HOMO to LUMO is essential for understanding the chemical nature of a molecule.^45^ Materials with large HOMO–LUMO energy gaps (*E*_gap_) are considered to be hard molecules with high kinetic strength and low chemical reactivity.^46^ In contrast, substances with smaller HOMO–LUMO energy gaps are soft molecules that are less stable and more reactive. These compounds have outstanding NLO characteristics and are very polarizable.^47^ In this study, we determined the *E*_HOMO_, *E*_LUMO_ and molecular orbital energy gaps (Δ*E*) of the above mentioned moieties at the M06/6-311G(d,p) level. Table 1 displays the information attained. Table S9† provides the results for HOMO−1/LUMO+1 and HOMO−2/LUMO+2.

**Table 1:** Calculated energies of *E*_HOMO_, *E*_LUMO_ and band gap (in eV) of the examined compounds

Compounds	*E*_HOMO_	*E*_LUMO_	Δ*E*
ATTR1	−5.612	−3.444	2.168
ATTD2	−5.089	−3.262	1.827
ATTD3	−5.111	−3.357	1.754
ATTD4	−5.083	−3.211	1.872
ATTD5	−5.098	−3.263	1.835
ATTD6	−5.110	−3.328	1.782
ATTD7	−5.108	−3.321	1.787
ATTD8	−5.085	−3.211	1.874

The evaluated HOMO–LUMO values of the reference compound (ATTR1) (−5.612/−3.444 eV) closely match the experimentally determined values of −5.50/−3.90 eV,^26^ indicating the suitability of the computational method used for the investigation of the compounds. All of the derivatives show lower band gap values than ATTR1 (2.168 eV). The values ranged from 1.874 to 1.754 eV. In the case of ATTD2, the energy gap value is reduced to 1.827 eV due to the incorporation of a donor species, *i.e.*, 4-methoxy-*N*-(4-methoxyphenyl)-*N*-phenylaniline, attached to the π-linker which enhances its electron donating ability towards the acceptor group, creating a strong push–pull mechanism (D-π-A). ATTD3 is the derivative which was found to have the lowest energy difference of 1.754 eV due to the introduction of 2-(2-methylene-6,7-dinitro-3-oxo-2,3-dihydro-1*H*-benzo[*b*]cyclopenta[*d*]thiophen-1-ylidene)malononitrile. The nitro group, which has a greater electron withdrawing capacity, remarkably reduced the band gap between the orbitals by firmly pushing the electronic cloud towards itself and deactivating the ring by lowering resonance. ATTD4 was designed by the incorporation of 2-(6,7-dichloro-2-methylene-3-oxo-2,3-dihydro-1*H*-benzo[*b*]cyclopenta[*d*]thiophen-1-ylidene)malononitrile and results in a higher energy gap value of 1.872 eV due to the halo groups that withdraw electrons through inductive effect and release electrons through resonance. The insertion of 2-(2-methylene-3-oxo-6,7-bis(trifluoromethyl)-2,3-dihydro-1*H*-benzo[*b*]cyclopenta[*d*]thiophen-1-ylidene)malononitrile as the acceptor moiety in ATTD5 results in a reduced energy gap value of 1.835 eV due to the highly electronegative nature of –F which enhances the electron withdrawing capacity of the acceptor groups. By substituting the end-capped acceptor molecules with 1-(dicyanomethylene)-2-methylene-3-oxo-2,3-dihydro-1*H*-benzo[*b*]cyclopenta[*d*]thiophene-6,7-disulfonic acid and 1-(dicyanomethylene)-2-methylene-3-oxo-2,3-dihydro-1*H*-benzo[*b*]cyclopenta[*d*]thiophene-6,7-dicarbonitrile in ATTD6 and ATTD7, respectively, the energy gap is further diminished. Specifically, the energy gaps are reduced to 1.782 and 1.787 eV, respectively. The introduction of the cyano (–CN) unit results in maximum charge transfer and a reduced band gap is observed due to resonance and stronger inductive impact. In the case of ATTD8, the energy gap value is reduced to 1.874 eV due to the presence of an efficient acceptor moiety, *i.e.*, dimethyl 1-(dicyanomethylene)-2-methylene-3-oxo-2,3-dihydro-1*H*-benzo[*b*]cyclopenta[*d*]thiophene-6,7-dicarboxylate. Here, the electron-withdrawing capacity of the carboxyl group is enhanced by the presence of electronegative oxygen atoms, causing a reduction in the band gap compared to ATTR1, although its energy gap is the highest among all the derivatives (ATTD2–ATTD8).

**Fig. 3:**
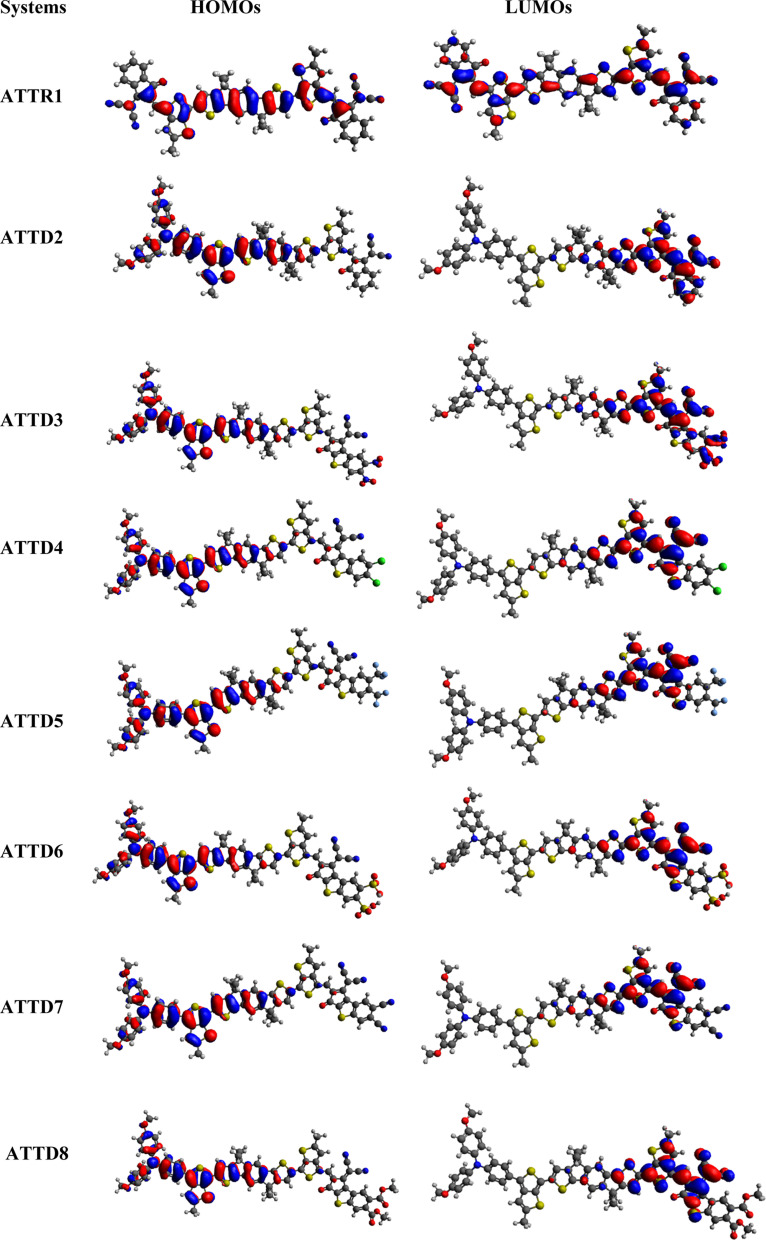
HOMOs and LUMOs of reference (ATTR1) and designed compounds (ATTD2–ATTD8).

The FMO study reveals that the designed derivatives (ATTD2–ATTD8) are good candidates for NLO properties due to their reduced band gaps. The energy difference between HOMO and LUMO decreases in the following sequence: ATTR1 > ATTD8 > ATTD4 > ATTD5 > ATTD2 > ATTD7 > ATTD6 > ATTD3. This indicates that modifying the acceptor components by introducing various electronegative substituents is an effective approach to decrease the band gap and enhance the NLO characteristics of NFAs. Transfer of charges in FMOs can be examined by analyzing their surfaces, as depicted in Fig. 3. The charge density in ATTR1 varies slightly across HOMO–LUMO, with the majority of the variability concentrated on the π-linker and to a lesser extent on the acceptor units. The electronic cloud of the HOMO is predominantly present over the donor and partially on the π-spacer in the proposed compounds (ATTD2–ATTD8). However, in the LUMO, the acceptor component usually comprises the majority of electron charge density, while a small portion is located over the π-spacers. All of the investigated compounds may be regarded as major constituents as a result of this charge transfer phenomena, among which ATTD3 has proved to be a promising NLO material. Fig. S2† highlights the inclusion of ICT in the analysis of the chromophores, alongside the assessment of orbital energies.

### Density of states (DOS) analysis

**Fig. 4:**
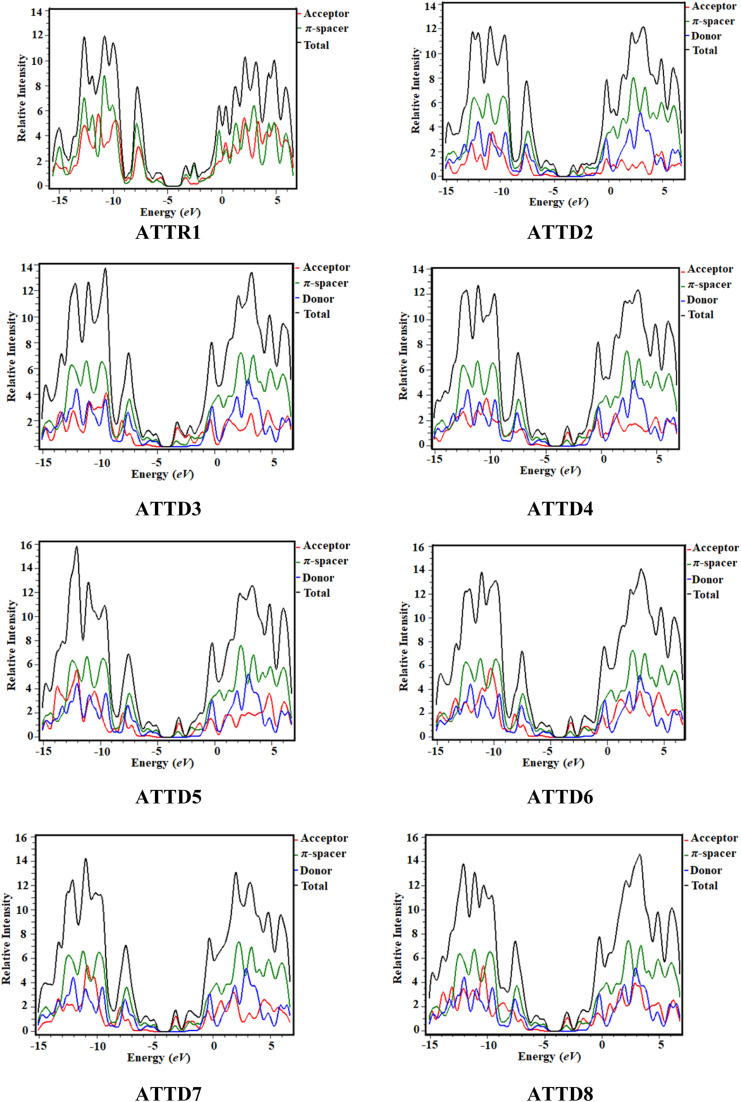
DOS plots of reference (ATTR1) and designed derivatives (ATTD2–ATTD8).

The density of states analysis (DOS) provides further confirmation for the delocalization of electrons observed in the HOMOs and LUMOs of the chromophores.^48^ The density of states measures the number of electronic states that are occupied by electrons at a specific energy level per unit of energy and volume. By performing DOS calculations, one can determine the energy gap and the overall distribution of energy levels with respect to energy, which gives information on the scattering of electrons.^49^ The reduction in the energy gap is a result of the enhanced electron-attracting capacity exhibited by the acceptor groups.^50^ In the DOS pictographs, the valence band exhibiting negative values is represented by the HOMO, whereas the conduction band (LUMO) shows positive values.^48,51^ The π-conjugated bridge connecting the electron-donating and electron-accepting moieties serves as a pivotal factor in fine-tuning the nonlinear optical characteristics while also exerting influence over the intra-molecular electron transfer processes.^52^ The optical and electrical characteristics of compounds are significantly altered by their band gap, which is an essential aspect of the band structure.^53^Fig. 4 shows the graphical outcomes of the DOSs for the studied compounds.

For the DOS computation, the molecules are fragmented. ATTR1 is divided into donor and acceptor segments, while ATTD2–ATTD8 are divided into three units: donor, π-spacer and acceptor portions. The HOMOs of ATTR1 are only present on the π-spacer, while the acceptor terminal has the main charge concentration of the LUMOs, as shown clearly in Fig. 3. The donor contributions towards the HOMOs are 69.7, 37.7, 40.8, 36.3, 36.1, 40.3, 38.0 and 38.1% in ATTR1 and ATTD2–ATTD8, respectively. Similarly, the donor contributions towards the LUMO are 47.3% in ATTR1 and 0.2% in all the designed species (ATTD2–ATTD8). The acceptor contributions were also calculated for the HOMOs and LUMOs of the respective compounds and were found to be 30.3, 2.3, 1.9, 2.2, 2.2, 1.9, 2.0, 2.1% and 52.7, 48.1, 54.1, 59.0, 56.9, 57.7, 56.8 and 59.3%, correspondingly. The π-linker also manifests distinct contributions of 60.0, 57.4, 61.5, 61.7, 57.9, 59.9 and 59.8% to the HOMOs while contributing 51.7, 45.7, 40.8, 43.0, 42.1, 43.0 and 40.5% towards the LUMOs of ATTD2–ATTD8, respectively. With the help of various strategies, variable acceptors can be facilitated for electronic transitions, as shown by the percentage contributions. By these outcomes, it could be seen from the DOS graphs that the HOMOs of all the designed chromophores are largely concentrated on the donor moiety as shown by the highest peaks of blue color positioned at nearly −12 eV. Similarly, the LUMOs are significantly positioned on acceptor part, indicated by the greater peaks of red color located near 3.2 eV, expect for the reference compound. For ATTR1, the highest charge density of the HOMO is identified to be −3.5 eV and highest density of the LUMO is determined to be 2.8 eV. This study reveals that compounds with different withdrawing acceptors exhibit distinct distribution patterns of electron density. Thus, the electronic properties of molecules are strongly influenced by the acceptor moieties.

### UV-visible analysis

To understand the optical properties of the system in chloroform solvent, a conductor like the polarizable continuum model^54^ is used to calculate the bands of absorption. This model considers solvent polarity and its ability to stabilize π → π* and n → π* transition states at appropriate energy levels, which contribute to the overall understanding of optical behavior.^55^ The light absorption process is significantly influenced by the electronic transition from the ground state to the first excited state. Consequently, an effective method for enhancing the light-absorption capacity of small-molecule acceptors involves elevating the electron density gradient.^56^ Tables S10–S17† provide the findings from the UV-Vis absorption spectra (in solvent) of ATTR1 and ATTD2–ATTD8, including the transition energy (*E*), oscillator strength (*f*_os_), absorption wavelengths (*λ*) and all possible molecular transitions along with their percentages.^57^Table 2 presents the maximum absorption wavelengths (*λ*_max_), highest oscillator strengths (*f*_os_), transition energies (eV) and major molecular orbital assessments (%) for the series of compounds under investigation. Thereby, we can get valuable information on the electronic properties and absorption characteristics of the above-mentioned compounds. Moreover, the absorption spectra were obtained, which show the relationship between the *λ*_max_ and the molar absorptivity constant values of the studied compounds in solvent and gas phase, as seen in Fig. S5.†

**Table 2:** Wavelength (*λ*_max_), excitation energy (*E*), oscillator strength (*f*_os_) and nature of molecular orbital contributions of compounds (ATTR1 and ATTD2–ATTD8) in chloroform^a^

Comp.	DFT, *λ* (nm)	*E* (eV)	*f*_os_	Major MO contributions
ATTR1	751.510	1.650	3.297	H → L (93%)
ATTD2	818.864	1.514	1.578	H → L (92%)
ATTD3	846.944	1.464	1.399	H → L (92%)
ATTD4	799.434	1.551	1.465	H → L (88%)
ATTD5	816.169	1.519	1.474	H → L (90%)
ATTD6	835.698	1.484	1.368	H → L (90%)
ATTD7	836.093	1.483	1.431	H → L (90%)
ATTD8	796.558	1.557	1.439	H → L (88%)

a MO = molecular orbital, H = HOMO, L = LUMO, *f*_os_ = oscillator strength, DFT = density functional theory.

The designed compounds ATTD2–ATTD8 have absorption values in chloroform solvent ranging from 846.944 to 796.558 nm and exhibit higher *λ*_max_ values than ATTR1 (751.510 nm), as displayed in Table 2. Moreover, the *λ*_max_ of the reference compound is close to the experimental value (600–940 nm).^26^ In the case of the derivatives (ATTD2–ATTD8), the existence of electron accepting groups significantly influences the absorbance shift towards longer wavelengths (bathochromic shift). By replacing the –CN groups of ATTD7 with –COOCH_3_ in ATTD8, the lowest absorbance value of 796.558 nm with an oscillation strength of 1.439 and transition energy of 1.557 eV is observed in ATTD8. By introducing a strong acceptor unit, the absorbance value in ATTD4 is raised to 799.434 nm with an oscillation strength of 1.551. The highest absorbance values are obtained in ATTD3 and ATTD7 at 846.944 and 836.093 nm with transition energies of 1.464 and 1.483 eV, representing 92% and 90% (HOMO → LUMO) electronic transitions, respectively. This cloud is due to the inclusion of the strong electron withdrawing cyano groups and nitro groups. Both groups reduce the energy band gap and lower the transition energies, which are important factors contributing to increased charge mobility and higher power conversion efficiency.^58^ The absorption value in ATTD2 is observed to be the lowest at 818.864 nm with an oscillation strength of 1.578. Similarly, the oscillation strength of ATTD6 is 1.368 and the absorption value is raised to 835.698 nm. There is no resonance effect in ATTD6, since there is no possibility of any orbitals or electron pairs overlapping with those of the ring due to the electron deficient sulphonic acid group. The descending order of *λ*_max_ in all the studied compounds in solvent phase is as follows: ATTD3 > ATTD7 > ATTD6 > ATTD2 > ATTD5 > ATTD4 > ATTD8 > ATTR1. Compounds ATTD2–ATTD8 exhibit greater red-shifts with lower oscillator strengths compared to ATTR1 due to the interaction of the electron-withdrawing groups on the acceptor moieties with the chloroform solvent. The energy difference between their excited and ground states is reduced as a result of this interaction, causing a bathochromic shift and improved optical characteristics.

In order to determine the photo-physical properties of the molecules in gaseous phase, a TD-DFT analysis is conducted using the M06 method at the 6-311G(d,p) basis set. Due to solvent effect, the maximum values for the investigated compounds in chloroform solvent are seen to be more red shifted than in gaseous phase. The major information from Tables S18–S25† is summarized in Table 3. When analyzed in the gaseous phase, the absorption maxima of the explored compounds are found in the range of 832.444 to 757.850 nm. The investigation showed that all the derivatives displayed superior optical properties compared to ATTR1. The highest absorption value (*λ*_max_) is obtained for ATTD3, *i.e.*, 832.444 nm, with an oscillation frequency of 1.035 and transition energy of 1.489 eV. It is the highest *λ*_max_ value among all the studied chromophores. However, the value obtained for the compound in the gaseous phase is less than the solvent *λ*_max_ value. Similarly, the HOMO–LUMO contributions for ATTD3 are 97% compared to 99% in the case of chloroform. The following decreasing order of *λ*_max_ is observed in the gaseous phase for the series of compounds under investigation: ATTD3 > ATTD7 > ATTD6 > ATTD5 > ATTD2 > ATTD4 > ATTD8 > ATTR1. The order is approximately similar to that of the solvent phase with the exception of ATTD5 and ATTD2 which show reverse order in the case of chloroform.

**Table 3:** Wavelength (*λ*_max_), excitation energy (*E*), oscillator strength (*f*_os_) and nature of molecular orbital contributions of compounds (ATTR1 and ATTD2–ATTD8) in gaseous phase^a^

Comp.	DFT, *λ* (nm)	*E* (eV)	*f*_os_	Major MO contributions
ATTR1	695.057	1.784	3.007	H → L (95%)
ATTD2	767.181	1.616	1.310	H → L (96%)
ATTD3	832.444	1.489	1.035	H → L (97%)
ATTD4	765.098	1.621	1.215	H → L (94%)
ATTD5	790.212	1.569	1.169	H → L (96%)
ATTD6	822.122	1.508	1.041	H → L (97%)
ATTD7	822.940	1.507	1.073	H → L (97%)
ATTD8	757.850	1.636	1.234	H → L (94%)

a MO = molecular orbital, H = HOMO, L = LUMO, *f*_os_ = oscillator strength, DFT = density functional theory.

Overall, the UV-Vis analysis proved that incorporating efficient acceptor moieties into the reference molecule structure can lead to chromophores with lower band gaps and wider absorption spectra, which results in a more desirable NLO response.

### Transition density matrix (TDM) and binding energy (*E*_b_) investigation

**Fig. 5:**
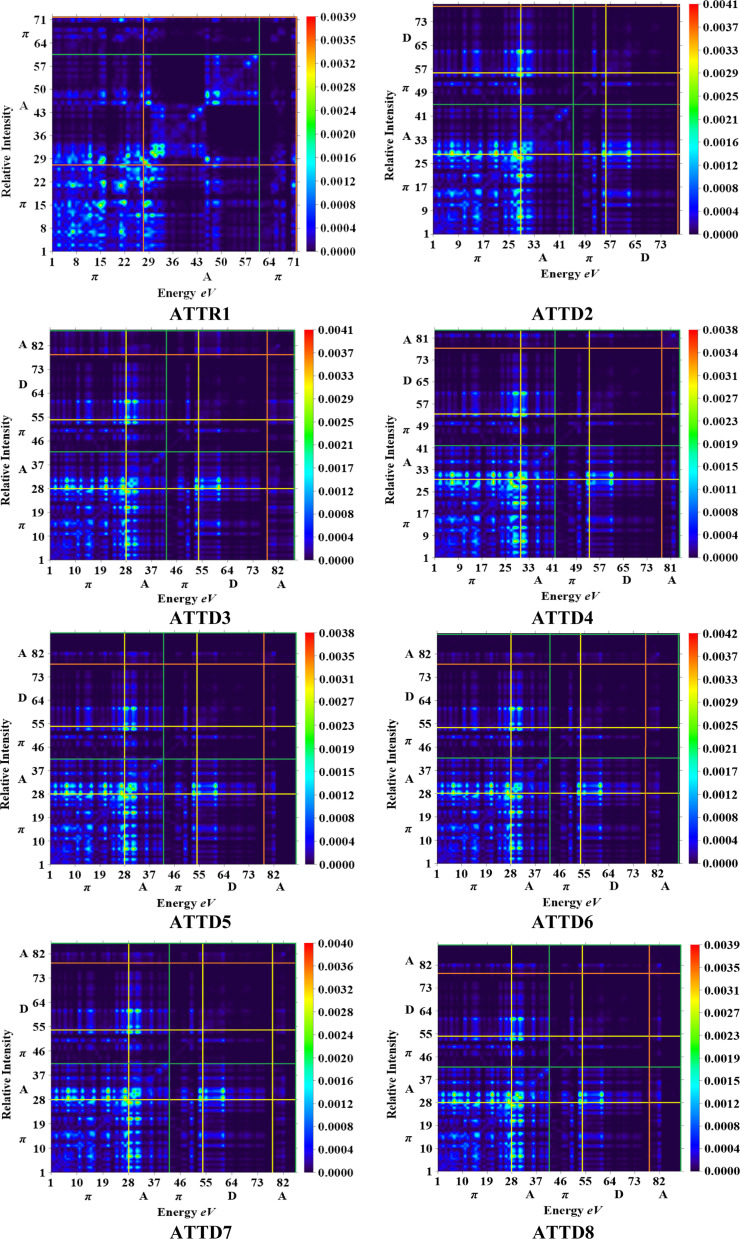
TDM graphs for ATTR1 and ATTD2–ATTD8.

The M06/6-311G(d,p) functional is used to calculate the TDM of the substances under investigation (ATTR1 and ATTD2–ATTD8). TDM assists in the evaluation of (a) the interaction between donor and acceptor moieties in the excited state, (b) electronic charge excitations and (c) the localization as well as delocalization of electron–hole pairs.^48^ An electrostatic potential map can be used to graphically represent the distribution of charges.^59^ The contribution of hydrogen atoms to electronic transitions is considered insignificant due to their low affinity in the determination of effective charge transfer. Due to their negligible influence on transitions compared to the other atoms, the H-atoms are neglected.^60^ The TDM diagrams used to explain the nature of the transition in each of the studied compounds are depicted in Fig. 5. The compounds are fragmented into three parts, *i.e.*, donor, linker and acceptor, to facilitate the understanding of the electronic charge transfer density. The TDM heat maps clearly show that charge is carried diagonally in all the derivatives and the flow of charge originates from the donor towards the central core π-spacer and ultimately reaches the end-capped acceptor.

According to the FMOs study, charge transfer occurs over the entire molecule in a considerable way, which causes TDM heat maps to fluctuate dramatically. These maps demonstrate that the charge coherence diagonally extended towards the terminal acceptor section *via* the π-bridge when the hole–electron pairs started to build up. In the TDM map, it also appears that excitation of coherence and electron–hole pair creation spread non-diagonally. These TDM heat maps exposed that all of the proposed chromophores had effective diagonal charge transfer coherence. The heat maps for ATTR1 displayed an increased electron concentration on both the donor and acceptor regions, depicted by the presence of green and red spots. Conversely, ATTD2 to ATDD8 portray an exceptional charge allocation observed towards the π-linker from donor, with only a small amount of charge detectable on the acceptor. The pictograph of TDM indicates that there is easier and more substantial dissociation of excitation in the excited state. This outcome holds significant importance for the development of NLO materials.

The binding energy (*E*_b_) is the one of most reliable characteristics to assess the optoelectronic capabilities, competence and exciton dissociation potential of compounds. It is simple to estimate the coulombic forces (also known as the relationship between the hole and the electron) with the help of *E*_b_, as binding energy and coulombic forces are directly related to one another. The weak coulombic interactions between the hole and electron caused by an exciton binding energy with a low value make excitation dissociation simple. Moreover, higher current charge density (*J*_sc_) and improved PCE are obtained by a lower value of *E*_b_. By calculating the difference between the first singlet excitation energy (*E*_opt_) and the HOMO-LOMO, one can determine the value of *E*_b_, as illustrated in the equation below.^60^

5 *E*_b_ = *E*_LUMO–HOMO_ − *E*_opt_

ATTR1 showed lower excitation binding energies and higher charge dissociation efficiencies than the modified compounds (ATTD2–ATTD8). As illustrated in Table 4, ATTD3 has the lowest binding energy and hence displays a greater flow rate of charges. In descending order, the studied chromophores' *E*_b_ values are as follows: ATTR1 > ATTD4 > ATTD8 > ATTD5 > ATTD2 > ATTD7 > ATTD6 > ATTD3. In summary, the designed chromophores exhibit lower binding energy values than ATTR1. These findings support the conclusions of TDM, indicating that these developed compounds have high polarizability and are appropriate for usage in the field of NLO.

**Table 4:** Computed *E*_LUMO-HOMO_, *E*_opt_ and *E*_b_ of ATTR1 and ATTD2–ATTD8 compounds. Units are in eV

System	*E*_H–L_	*E*_opt_	*E*_b_
ATTR1	2.168	1.650	0.518
ATTD2	1.827	1.514	0.313
ATTD3	1.754	1.464	0.290
ATTD4	1.872	1.551	0.321
ATTD5	1.835	1.519	0.316
ATTD6	1.782	1.484	0.298
ATTD7	1.787	1.483	0.304
ATTD8	1.874	1.557	0.317

### Global reactivity descriptors (GRDs)

The strength of FMOs (*E*_gap_ = *E*_LUMO_ − *E*_HOMO_) is useful for determining global reactivity parameters, which include the chemical potential (*μ*),^61^ ionization potential (IP),^62^ electron affinity (EA), global softness (*σ*),^63^ hardness (*η*),^64^ electronegativity (*X*),^65^ and electrophilicity index (*ω*)^66^ of the examined compounds, and is calculated using eqn (6)–(12), whose results are depicted in Table 5.

6 IP = −*E*_HOMO_

7 EA = −*E*_LUMO_

8
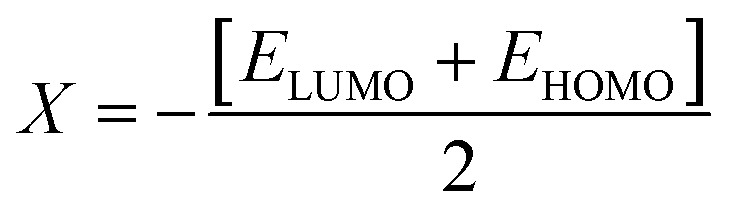

9 *η* = IP − EA

10
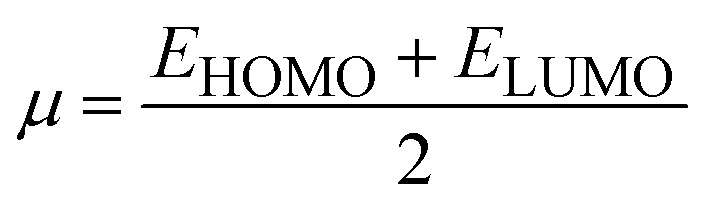
11
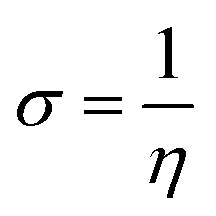
12
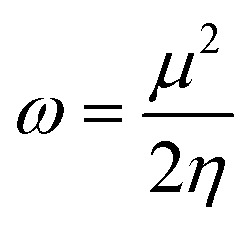


**Table 5:** Global reactivity descriptors of ATTR1 and ATTD2–ATTD8. Units are in eV^a^

Compounds	IP	EA	*X*	*η*	*μ*	*ω*	*σ*	Δ*N*_max_
ATTR1	5.612	3.444	4.528	2.168	−4.528	4.728	0.461	4.177
ATTD2	5.089	3.262	4.175	1.827	−4.175	4.770	0.547	0.382
ATTD3	5.111	3.357	4.234	1.754	−4.234	5.110	0.570	0.389
ATTD4	5.083	3.211	4.147	1.872	−4.147	4.588	0.534	4.430
ATTD5	5.098	3.263	4.180	1.835	−4.180	4.760	0.544	4.558
ATTD6	5.11	3.328	4.219	1.782	−4.219	5.150	0.561	4.735
ATTD7	5.108	3.321	4.214	1.787	−4.214	4.968	0.559	4.718
ATTD8	5.085	3.211	4.148	1.874	−4.148	4.590	0.533	4.426

a IP = ionization potential, EA = electron affinity, *X* = electronegativity, *μ* = chemical potential, *η* = global hardness, *σ* = global softness and *ω* = global electrophilicity.

The value of Δ*N*_max_ = −*μ*/*η* represents the propensity of a molecule to absorb more electrical charge from its surroundings.^67^ The ionization potential, which expresses the ability of an atom to donate electrons, is equal to the energy required to remove an electron from the HOMO orbital. The electrophilic strength of substances is evaluated using this parameter. In addition, the chemical potential, hardness and compound stability are directly correlated with the HOMO/LUMO energy gap, while reactivity is inversely related. Smaller energy gap molecules are therefore thought to be more reactive, less stable and softer molecules that compete more intensely for the optimal NLO response.^68–70^ Higher values of chemical potential (*μ*) indicate greater stability and lower reactivity when analyzing the stability and reactivity of compounds. Conversely, more negative *μ* values indicate less stability and higher reactivity in D–π–A structures.^71^ Because of the increased stability, the molecules become harder and less reactive. In contrast, molecules with small energy gaps are softer, more flexible, reactive, and less kinetically and physically stable. Because of this, suitable NLO materials with stronger polarizabilities have a smaller energy gap.^72^Table 5 shows that the reference chromophore has higher values for hardness and chemical potential than all its derivatives. There is a direct relationship between the band gap of a compound and its hardness, chemical potential and stability, while an inverse relationship exists with reactivity. Hence, a molecule with a larger energy gap is regarded as having greater strength, stability and lower reactivity. Conversely, molecules with small band gaps are softer, more reactive and less stable. The overall decreasing order of hardness is as follows: ATTR1 > ATTD8 > ATTD4 > ATTD5 > ATTD2 > ATTD7 > ATTD6 > ATTD3 with values of 2.168, 1.874, 1.872, 1.835, 1.827, 1.787, 1.782, and 1.754 eV, respectively. Arranged in descending order of softness, the compounds can be ranked as follows: ATTD3 > ATTD6 > ATTD7 > ATTD2 > ATTD5 > ATTD4 > ATTD8 > ATTR1 with corresponding values of 0.570, 0.561, 0.559, 0.547, 0.544, 0.534, 0.533 and 0.461 eV, respectively. The electron accepting capacity of the molecules under consideration is determined by their electron affinity (EA) values, which are closely connected to the energies of their LUMOs. Ionization potential values for ATTR1 are determined to be larger than those of ATTD2–ATTD8, indicating that the derivatives have maximum electron donating capability. In the designed derivatives ATTD2–ATTD8, the Δ*N*_max_ values are in the range of 0.382–4.735 eV. The computed results show that these compounds retain favorable global reactivity parameters that are necessary for exhibiting a nonlinear optical response.

### Natural bonding orbitals (NBOs) investigation

The most suitable method is NBO analysis, which enables the interpretation of the nucleophilic and electrophilic hyper-conjugative interactions, other bonding interactions and mode of electronic transitions.^73^ The NBO study helps to comprehend the uniform image of the donor–acceptor framework created by clarifying the charge density shift from fully filled to half-filled non-bonded NBOs.^74^ The presence of positive charges on the π-spacers indicates their capacity to facilitate the transfer of electrons from donor to acceptor, while negative charges on acceptors signify their aptitude for accepting electrons.^75^ By applying second-order perturbation theory, we can explore the stabilization energy of molecules and, for this purpose, eqn (13) is utilized,^76^13
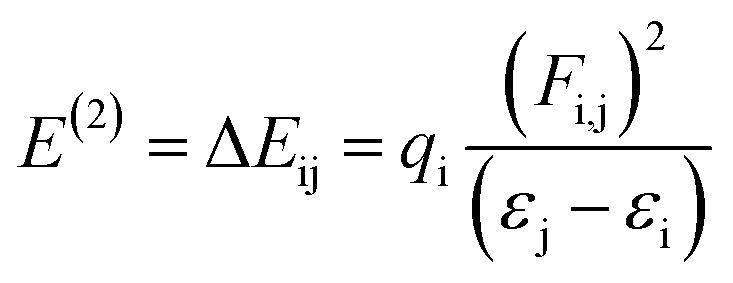
where *E*^(2)^ is the energy of stabilization, subscripts i and j stand for the donor and acceptor, respectively, and *ε*_i_, *ε*_j_, *F*_i,j_ and *q*_i_ signify the diagonal and off-diagonal NBO Fock matrix elements and orbital occupancy, respectively. The major results of the NBO analysis are given in Table S39.†

Among the molecular electronic transitions, π → π* transitions are the most dominant in charge transfer within the molecules compared to σ → σ*, LP → π* and LP → σ* transitions. In reference chromophore ATTR1, π → π* transitions possess the stabilization energy of 33.74 kcal mol^−1^ while the lowest energy π → π* transitions are 0.78 kcal mol^−1^. The feeble transitions (σ → σ*) possess a highest energy transition of σ(C33–H53) → σ*(S26–C31) at 10.71 kcal mol^−1^ and a lowest energy transition of σ(C67–N68) → σ*(C56–C57) at 0.5 kcal mol^−1^. Among lone pair transitions, LP → π* transitions have a maximum stabilization energy value of 28.61 kcal mol^−1^ which occurs for LP2(S26) → π*(C24–C25), while LP → σ* possess a highest value of 20.79 kcal mol^−1^ observed in the LP2(O52) → σ*(C43–C47) transition. Table S26† provides several examples of transitions exhibiting conjugation.

In ATTD2, 34.84 kcal mol^−1^ is the uppermost stabilization energy detected for π(C27–C28) → π*(C33–C34) transitions, while transitions such as π(C107–C108) → π*(C97–C99) are observed to have the least energy, *i.e.*, 0.76 kcal mol^−1^. The major σ → σ* transitions are σ (C33–H35) → σ*(C27–S30) at 10.85 kcal mol^−1^ and the lowest transitions are σ(C73–S83) → σ*(C28–C29) at 0.51 kcal mol^−1^. Moreover, the LP → π* transition from LP2 (O118) → π*(C102–C104) has the highest value of 32.47 kcal mol^−1^, while the transition from LP2(O51) → σ*(C42–C46) has the maximum stabilization energy of 20.86 kcal mol^−1^. Several transitions of ATTD2 demonstrating conjugation are presented in the Table S27.†

Compound ATTD3 exhibits a maximum π → π* transition energy at 36.23 kcal mol^−1^ for π(C27–C28) → π*(C33–C34) and a minimum energy of 0.76 kcal mol^−1^ for π(C101-102) → π*(C91–C93) transitions. The maximum and minimum stabilization energy values for σ → σ* transitions are 10.77 kcal mol^−1^ obtained for σ(C33–H35) → σ*(C27–S30) and 0.51 kcal mol^−1^ for σ(C69–S79) → σ*(C28–C29) transitions. Additionally, LP → σ* exhibits the greatest stabilization energy of 22.65 kcal mol^−1^ for LP2(O47) → σ*(C41–C42), while the LP → π* transition has the highest energy of 32.44 kcal mol^−1^ for LP2(O112) → π*(C96–C98). Table S28† shows a variety of conjugations with different transitions for ATTD3.

The chemical structure of chromophore ATTD4 reveals a highest stabilization energy of 34.1 kcal mol^−1^ for π(C27–C28) → π*(C33–C34), while the lowest energy is observed at 0.77 kcal mol^−1^ for π(C45–N46) → π*(C43–N44) transitions. The highest stabilization energy for σ → σ* transitions occurs at 10.58 kcal mol^−1^ for σ(C33–H35) → σ*(C27–S30) and the lowest value is observed at 0.51 kcal mol^−1^ for σ(S17–C20) → σ*(C10–C21) transitions. The highest energy for LP → σ* transitions occurs at 22.21 kcal mol^−1^ for LP2(O47) → σ*(C41–C42); for LP → π* transitions, the highest value is 32.59 kcal mol^−1^ for LP2(O111) → π*(C104–C108). Table S29† presents various transitions for ATTD4.

The largest stabilization energy, *i.e.*, 35.22 kcal mol^−1^, of the π → π* type is obtained for π(C27–C28) → π*(C33–C34) transitions in chromophore ATTD5, while the lowest energy transition occurs at 0.78 kcal mol^−1^ for π(C45–N46) → π*(C43–N44). Furthermore, the highest stabilization energy for the σ → σ* type is 10.72 kcal mol^−1^ observed for σ(C33–H35) → σ*(C27–S30) and the lowest recognized value is 0.5 kcal mol^−1^ for σ(C45–N46) → σ*(C36–C37) transitions. The LP → π* transition has a maximum energy of 32.51 kcal mol^−1^ observed in LP(O112) → π*(C96–C98) and LP → σ* has a maximum stabilization energy of 22.37 kcal mol^−1^ for LP(O47) → σ*(C41–C42) transitions. In Table S30,† we report a variety of examples indicating conjugation in ATTD5 through distinct sorts of transitions.

In the same way, the electronic transitions in ATTD6 are investigated and the results show that π(C27–C28) → π*(C33–C34) transitions possess the greatest stabilization energy of 36.16 kcal mol^−1^, while the lowest value of 0.77 kcal mol^−1^ is obtained for π(C101–C102) → π*(C91–C93). The σ → σ* weak interaction transitions, specifically σ(C33–H35) → σ*(C27–S30) exhibit a maximum stabilization energy of 10.74 kcal mol^−1^, while the minimum energy value of 0.5 kcal mol^−1^ is obtained for σ(C45–N46) → σ*(C36–C37) transitions. The resonance phenomena in the compound results in the LP(O131) → σ*(S128–O129) and LP(O112) → π*(C96–C98) transitions having maximum stabilization energies of 32.44 and 2.68 kcal mol^−1^, respectively. Table S31† provides several instances of conjugation through different types of transitions in ATTD6.

The study of the ATTD7 molecule reveals that the highest stabilization energy of 35.93 kcal mol^−1^ is associated with π(C27–C28) → π*(C33–C34) transitions, whereas π(C45–N46) → π*(C43–N44) are observed to have the lowest value, *i.e.*, 0.76 kcal mol^−1^. The weak interaction transitions, namely σ(C33–H35) → σ*(C27–S30) and σ(C69–S79) → σ*(C28–C29), exhibit the highest and lowest stabilization energies, *i.e.*, 10.72 and 0.51 kcal mol^−1^. In addition, resonance effects in the molecule lead to the LP(O47) → σ*(C41–C42) and LP(N90) → π*(C83–C87) transitions having stabilization energies of 22.62 and 32.73 kcal mol^−1^, respectively. Different transitions are displayed in Table S32† for ATTD7.

The following significant electronic transitions take place in the final derivative ATTD8: π(C27–C28) → π*(C33–C34), π(C101–C102) → π*(C91–C93), σ(C33–H35) → σ*(C27–S30), σ(C123–H124) → σ*(125-C128), LP(O132) → σ*(C131–O133) and LP(O130) → π*(C128–O129), with stabilization energies of 34.19, 0.72, 10.61, 0.5, 33.13 and 50.66 kcal mol^−1^, respectively. Other transitions are recorded in Table S33.†

The above data demonstrate that the non-covalent interactions between filled and unfilled orbitals are very important in stabilizing the series of compounds under study (ATTR1 and ATTD2–ATTD8). Hence, NBO analysis of these compounds shows that robust ICT and extended hyper-conjugation play a significant role in stabilizing these molecules and offer evidence of charge transfer features that are essential for prospective NLO capabilities.

### Electron–hole analysis

In order to understand the transfer of the electronic cloud in a molecule, hole–electron analysis is a very useful and widely used tool.^77,78^ Hole analysis was performed for all designed compounds and is shown in Fig. S3.†ATTR1 shows hole transfer at the carbon atom of the –CN group in the acceptor region at an intensity of 0.057 and electron transfer at the carbon atom in the thiophene group at an intensity of 0.072. The electron withdrawing nature of the thiophene ring acts as a π–bridge between the donor and acceptor atoms for electronic charge transfer. ATTD2 and ATTD3 show electron transfer from the donor towards the carbon atom of the thiophene group with intensities of 0.123 and 0.152, respectively. Here, the change in intensity shows the greater electron withdrawing capacity of the acceptor region through the π-spacer. Compounds ATTD5–ATTD8 also show electron transfer in the intensity range of 0.148–0.155, while exceptional and efficient electron transfer is found in ATTD4 due to the presence of –Cl groups in the acceptor region, giving the greatest intensity of 0.156. Therefore, ATTD4 is an efficient compound for NLO applications. However, in the case of ATTD7, where the –CN group has been replaced by a carboxyl group, the electron intensity is 0.155. This might be because of the resonance and a strong negative inductive effect. Overall, this investigation shows that only the reference compound shows the hole band and hole intensity as well as electron band intensity. Consequently, the reference compound acts as both a hole-type and electron-type material, while all the derivative compounds show themselves as electron-type materials.

### Natural population investigation (NPA)

According to natural population research, molecules with more electronegative atoms like O and N possess different electrical density distributions across the structure.^79^ The M06/6-311G(d,p) basis set was used for natural population-based analysis on NBOs using TD-DFT. The phenomenon is related to charge transformation and the electronegativity equalization process is performed in reaction to access the electrostatic capability on the outside surfaces of the structure.^80,81^ According to the charge distribution analysis, in all the compounds, the fluorine and nitrogen atoms connected to oxygen have negative charges, whereas the S-atoms have positive charges (see Fig. S4†). In addition, all hydrogen atoms in ATTR1 and ATTD2–ATTD8 are positively charged. According to the study of natural population, the charge distribution of all hydrogen atoms is uniform. Certain carbon atoms display negative charges, whereas others exhibit positive charges, owing to their individual electron donating or accepting nature. The electrostatic attraction between the atoms can be predicted by the partial charge separation on the chemical structures of the designed compounds and may have a significant impact on inter- and intramolecular interactions. The extensive investigation of NBO charges indicated that the oxygen and nitrogen atoms are primarily responsible for the asymmetrical distribution of charges in the examined compounds.

### Non-linear optics (NLO)

Optoelectronic devices, communication, networking and signal processing often rely on NLO products. The optical behavior of these products is determined by the electrical characteristics of the molecule, which are related to both their linear and nonlinear responses.^73^ In organic compounds, the NLO reaction occurs through asymmetric polarizability. The inclusion of electron-withdrawing and electron-donating moieties in the molecules at the proper locations creates a significant push–pull architecture which may produce the improved NLO response. Additionally, these electron-withdrawing and electron-donating groups are joined to the π-conjugated linker, which improves the NLO behavior. The potency of the optical reaction is contingent upon the electrical attributes of the entire material. In the context of molecules, these attributes find expression in polarizability (*α*) for linear reactions and hyperpolarizabilities (*β*, *γ*, *etc.*) for nonlinear responses.^82^ As such, these properties constitute essential metrics for evaluating the optical capabilities of molecules and should be subject to measurement for a comprehensive assessment.^83^ According to published research, the energy difference between the LUMO and HOMO has an effect on the polarizability of molecules. Compounds having a narrower energy gap have higher hyper-polarizability and linear polarizability values.^84^ In order to perform optical analyses of the designed compounds, their electronic characteristics are evaluated. This includes measuring linear response properties like linear polarizability 〈*α*〉 and nonlinear response properties like first and second hyper-polarizabilities (*β*_tot_ and *γ*_tot_).^85^ The polarizability of organic chromophores can be estimated using the dipole moment (*μ*).^86^ The evaluated values of *α*_tot_, *β*_tot_, *μ*_tot_ and *γ*_tot_ for the designed compounds are represented in Table 6. Additional results for all tensors are found in Tables S34–S37.†

**Table 6:** Dipole moment (*μ*_tot_), average polarizability 〈*α*〉, and first (*β*_tot_) and second-order hyper-polarizabilities (*γ*_tot_)^a^

System	*μ*_tot_	〈*α*〉 × 10^−22^	*β*_tot_ × 10^−27^	*γ*_tot_ × 10^−31^
ATTR1	0.46	3.05	0.05	0.72
ATTD2	18.38	2.97	6.98	1.29
ATTD3	27.26	3.19	8.23	1.66
ATTD4	19.33	3.09	6.03	1.10
ATTD5	22.83	3.11	6.86	1.24
ATTD6	24.79	3.21	7.64	1.45
ATTD7	28.14	3.20	7.84	1.49
ATTD8	16.49	3.13	5.96	1.08

a *μ*_tot_ in D; 〈*α*〉, *β*_tot_ and 〈*γ*〉 in esu.

In order to evaluate the NLO properties of ATTR1 and its derivatives ATTD2–ATTD8, it is important to assess their linear and NLO responses. The dipole polarizability has three tensors along the *x*, *y*, and *z* directions. The molecules were analyzed for their dipole moments and all derivatives show higher dipole moments than ATTR1 (0.46 D). The ATTD5 compound possesses the highest value of 28.14 D and ATTD8 displays the lowest value of 16.49 D among all the derivatives. This is due to the nature of the trifluoromethyl (–CF_3_) and acetate (–COOCH_3_) groups, respectively, on their acceptor moieties. Additionally, in comparison to the standard molecule (*para*-nitroaniline), which has a dipole moment of 4.9662 D,^87^ all the tailored molecules possess significantly higher dipole moments which are 0.09, 3.70, 5.48, 3.89, 3.55, 4.59, 4.99, 5.66 and 3.32 times greater, respectively, than that of the standard molecule. The chromophores are arranged in descending order based on their respective values of *μ*_tot_ as follows: ATTD7 > ATTD3 > ATTD6 > ATTD5 > ATTD4 > ATTD2 > ATTD8 > ATTR1.

The average polarizability 〈*α*〉 values for the studied compounds are depicted along with their major contributing tensors in Table S35.†ATTD3 and ATTD7 possess the highest 〈*α*〉 values of 3.19 × 10^−22^ esu and 3.20 × 10^−22^ esu, respectively, among all the designed chromophores. These values correspond to their lower energy gaps, *i.e.*, 1.754 and 1.787 eV, respectively. Similarly, the compound ATTD3 attained the highest first hyperpolarizability (*β*_tot_) of 8.23 × 10^−27^ esu which is in accordance with its NLO characteristics. This information demonstrates that strong acceptor moieties enhance the polarizability. Table S36† presents the tabulated values of the second-order nonlinear optical properties and their respective tensors for chromophores ATTR1 and ATTD2–ATTD8 with thiophene-based linkers. Increasing the number of thiophene rings in the π-spacer caused a progressive increase in *β*_tot_. As the energy gap between the HOMO and LUMO reduces in compounds, their hyper-polarizability increases.^75^ATTD3 revealed the highest *β*_tot_ value compared to the standard molecule (p-NA) of 3.61 × 10^−31^.^87^ The other derivatives also possess higher values, *i.e.*, 0.13 × 10^−5^, 1.93 × 10^−3^, 2.27 × 10^−3^, 6.03 × 10^−3^, 1.90 × 10^−3^, 2.11 × 10^−3^, 2.17 × 10^−3^ and 1.65 × 10^−3^ times larger than that of the standard molecule. The *β*_tot_ values of the aforementioned chromophores are observed to be higher due to intra-molecular charge transfer from electron donating to electron withdrawing groups along the *β*_*xxx*_, *β*_*xxy*_, *β*_*xyy*_, *β*_*yyy*_, *β*_*xxz*_, *β*_*yyz*_, *β*_*xzz*_, *β*_*yzz*_ and *β*_*zzz*_ co-ordinates compared to reference compound ATTR1, which has the lowest *β*_tot_ of 0.05 × 10^−27^ esu, potentially due to constraints in the charge transfer. A compatible collaboration between the molecular structures and *β*_tot_ is also observed. The decreasing order of *β*_tot_ values for all compounds is ATTD3 > ATTD7 > ATTD6 > ATTD2 > ATTD5 > ATTD4 > ATTD8 > ATTR1. The polarizability is influenced by the energy gap, and a shorter energy gap coupled with higher polarizability is known to yield significant nonlinear optical responses, which are associated with larger hyperpolarizabilities.^88^ The higher value of *β*_tot_ for ATTD3 indicates better NLO properties which make it a promising candidate for applications in optical technologies.

The estimation of nonlinear optical response relies on the third-order nonlinear optical parameter *γ*_tot_ which is studied as a fundamental feature.^89^ In nonlinear optical compounds, it is considered a crucial parameter. Among the analyzed moieties, ATTR1 possesses the lowest *γ*_tot_ value of 0.72 × 10^−31^ esu with the following tensor values along the *γ*_*x*_, *γ*_*y*_, and *γ*_*z*_ orientations: 0.72 × 10^−31^, 0.10 × 10^−33^, and 0.43 × 10^−35^ esu, respectively. Among the designed derivatives, ATTD3 exhibited the greatest *γ*_tot_ value of 1.66 × 10^−31^ esu with the largest contribution of *γ*_*x*_ (1.63 × 10^−31^ esu). ATTD8 possesses the lowest value of 1.08 × 10^−31^ esu. The decrease in the second-order hyper-polarizability can be attributed to the presence of carboxyl groups on the acceptor component. Mesomeric effects and -*I* properties of these groups lead to a reduction in the *γ*_tot_. The decreasing order of *γ*_tot_ values for all the examined compounds is ATTD3 > ATTD7 > ATTD6 > ATTD2 > ATTD5 > ATTD4 > ATTD8 > ATTR1. Table S37† displays the second hyper-polarizabilities and major contributing tensors (esu) of the studied compounds.

## Conclusion

The primary objective of this study was to elucidate the effect of benzothiophene-based acceptor units on the electrical, photophysical, and nonlinear optical (NLO) characteristics of newly designed chromophores ATTD2–ATTD8. Interestingly, all of the derivatives (ATTD2–ATTD8) showed a significant reduction in their HOMO–LUMO energy gaps (1.827–1.754 eV) with bathochromic shifts (818.864 nm) compared to the reference compound (Δ*E* = 2.168 eV and *λ*_max_ = 751.510 nm) as the conjugation length increased. The NBO analysis showed that the increased conjugation improved the CT process in chromophores. Additionally, the GRP findings exhibited a higher value of softness with a greater value of Δ*N*_max_ and smaller hardness in the derivatives than in ATTR1. The chromophores also demonstrated significant NLO response compared to ATTR1. Remarkable results can be observed in the values of 〈*α*〉, *γ*_tot_ and *β*_tot_ when the designed moieties are compared to ATTR1. Particularly, significantly promising results are achieved in ATTD3, which exhibits a 〈*α*〉 of 3.19 × 10^−22^, *γ*_tot_ of 1.66 × 10^−31^ and *β*_tot_ of 8.23 × 10^−27^ esu. It can be inferred that modifying the molecular structure of all the derivative compounds with benzothiophene-based acceptors plays a pivotal role in precisely adjusting the non-linear optical (NLO) properties. This research contributes to a comprehensive understanding of the connection between structure and properties in regards to electronic characteristics. Furthermore, this study is likely to inspire experimental researchers to synthesize these compounds owing to their noteworthy NLO attributes.

## Data availability

All data generated or analyzed during this study are included in this published article and its ESI.†

## Conflicts of interest

There are no conflicts of interest to declare.

## Supplementary Material

RA-013-D3RA04580C-s001
