# Supplementary material for: Exploration of linear and third-order nonlinear optical properties for donor–π-linker–acceptor chromophores derived from ATT-2 based non-fullerene molecule
Source: RSC Adv. 2023 Nov 1;13(45):31855–72. doi: 10.1039/d3ra04580c (PMC10618729; doi:10.1039/d3ra04580c)
Supplement: RA-013-D3RA04580C-s001 [file RA-013-D3RA04580C-s001.pdf]

Electronic Supplementary Material (ESI) for RSC Advances.
This journal is © The Royal Society of Chemistry 2023

Supplementary Information

Exploration of Linear and Third-order Nonlinear Optical Properties for Donor-π linker-Acceptor Chromophores Derived from ATT-2 Based Non-fullerene Molecule

Muhammad Sagir,[1] Kalsoom Mushtaq,[2,3] Muhammad Khalid,*[2,3] Mashal Khan,[2,3] Muhammad Bilal Tahir,[4] Ataualpa A. C. Braga,[5]

[1]Institute of Chemical and Environmental Engineering, Khwaja Fareed University of Engineering & Information Technology, Rahim Yar Khan, 64200, Pakistan

[2]Institute of Chemistry, Khwaja Fareed University of Engineering & Information Technology, Rahim Yar Khan, 64200, Pakistan

[3]Centre for Theoretical and Computational Research, Khwaja Fareed University of Engineering & Information Technology, Rahim Yar Khan, 64200, Pakistan

[4]Institute of Physics, Khwaja Fareed University of Engineering & Information Technology, Rahim Yar Khan, 64200, Pakistan

[5]Departamento de Qu´ımica Fundamental, Instituto de Qu´ımica, Universidade de Sao˜ Paulo, Av. Prof. Lineu Prestes, 748, Sao Paulo, 05508-000, Brazil

*Corresponding author's E-mail address:

Dr. Muhammad Khalid (khalid@iq.usp.br)

Table S1: Cartesian coordinates of **ATTR1.**

Atom	X-axis	Y-axis	Z-axis
C	-0.32841	-1.38659	0.071583
C	0.987857	-0.97567	0.071205
C	1.314433	0.399344	0.072734
C	0.328379	1.386507	0.071646
C	-0.98788	0.975588	0.07121
C	-1.31446	-0.39943	0.072669
H	-0.59179	-2.44215	0.071861
H	0.591763	2.442065	0.071957
C	-2.75294	-0.47503	0.076341
C	-3.3118	0.784515	0.073734
C	-4.70572	0.771678	0.083639
H	-5.33353	1.657391	0.093914
C	3.311776	-0.7846	0.073786
C	4.705695	-0.77175	0.083693
C	5.222884	0.513981	0.09422
H	5.333518	-1.65745	0.093934
S	-3.9363	-1.71879	0.088532
S	3.936247	1.718716	0.088625
C	2.752906	0.474947	0.076424

C	-5.22292	-0.51404	0.09416
C	-2.23735	1.841134	0.071518
C	2.23732	-1.84122	0.071514
C	-8.5806	-2.13978	0.228462
C	-7.17471	-2.14801	0.256742
C	-6.59723	-0.89675	0.103412
S	-7.82843	0.323573	-0.09209
C	9.132428	0.853809	0.048027
C	8.580556	2.139758	0.228311
C	7.174667	2.147965	0.256769
S	7.828384	-0.3236	-0.09223
C	-9.13248	-0.85381	0.048323
C	6.597179	0.896704	0.103452
C	-10.5141	-0.66275	-0.00671
C	10.51402	0.662733	-0.00696
C	11.33509	-0.43662	-0.16559
H	11.02439	1.614006	0.105084
C	12.78172	-0.43934	-0.20011
C	13.64621	0.631379	-0.0948
C	13.40661	-4.60411	-0.7195
C	14.5477	-3.81216	-0.64905
C	14.47201	-2.43232	-0.47902
C	13.21618	-1.84364	-0.37936
C	12.0805	-2.6555	-0.45255
C	12.1485	-4.02234	-0.62011
H	15.38704	-1.85801	-0.42875
H	11.23745	-4.61052	-0.67091
C	10.87417	-1.81918	-0.32631
C	15.05957	0.504365	-0.14774
N	16.21402	0.453526	-0.18501
C	13.22982	1.978088	0.077059
N	12.92553	3.085118	0.218368
O	9.725823	-2.22136	-0.35332
H	-11.0245	-1.61401	0.105355
C	-11.3351	0.436628	-0.16536
C	-10.8741	1.81918	-0.3259
C	-12.7817	0.439425	-0.20004
C	-13.6463	-0.63123	-0.09487
O	-9.72569	2.221298	-0.35265
C	-13.2161	1.843739	-0.37942
C	-12.0803	2.655549	-0.45245
C	-12.1483	4.022385	-0.62002
C	-13.4064	4.604189	-0.71959
C	-14.5475	3.812288	-0.64928
C	-14.4719	2.432447	-0.47922
H	-11.2372	4.610566	-0.67069
H	-15.3869	1.858128	-0.42901
C	-13.23	-1.97794	0.077252

N	-12.9257	-3.08495	0.218797
C	-15.0596	-0.50418	-0.1483
N	-16.214	-0.4533	-0.18598
C	2.311373	-2.70961	1.327119
H	1.484149	-3.42744	1.347644
H	3.250703	-3.27365	1.345095
H	2.259441	-2.09625	2.232511
C	2.313412	-2.70842	-1.18471
H	1.486173	-3.42618	-1.20733
H	2.263115	-2.09424	-2.08963
H	3.252873	-3.2723	-1.20144
C	-2.31142	2.709448	1.327183
H	-1.4842	3.427286	1.347766
H	-3.25076	3.273482	1.34518
H	-2.2595	2.09603	2.232534
C	-2.31342	2.708412	-1.18464
H	-2.2631	2.094301	-2.0896
H	-3.25288	3.272282	-1.20135
H	-1.48619	3.426186	-1.20721
C	-9.14007	-3.43563	0.401992
H	-10.2019	-3.66233	0.413542
C	-8.19354	-4.39659	0.558506
C	9.140036	3.435601	0.401816
H	10.20184	3.662307	0.413288
C	8.193516	4.396548	0.55848
C	8.396431	5.85167	0.763727
H	7.962151	6.18506	1.711959
H	9.464016	6.082102	0.774941
H	7.926538	6.436645	-0.03361
C	-8.39645	-5.85172	0.763729
H	-7.92648	-6.43668	-0.03357
H	-7.96224	-6.1851	1.711994
H	-9.46403	-6.08216	0.77486
S	-6.55297	-3.76241	0.502303
S	6.552938	3.762369	0.502405
H	15.52486	-4.27749	-0.7279
H	13.50365	-5.67657	-0.85172
H	-15.5246	4.277651	-0.72824
H	-13.5033	5.676658	-0.85184

Table S2: Cartesian coordinates of **ATTD2.**

Atom	X-axis	Y-axis	Z-axis
C	1.552638	1.449256	0.125102
C	2.866492	1.025011	0.111581
C	3.180835	-0.34947	0.041245
C	2.182847	-1.32536	-0.01368
C	0.872378	-0.90209	-0.0016
C	0.555646	0.475533	0.065535

H	1.300415	2.50642	0.176608
H	2.435653	-2.38246	-0.06769
C	-0.88253	0.561947	0.052683
C	-1.45114	-0.68503	-0.02196
C	-2.8515	-0.66347	-0.06818
H	-3.48434	-1.54225	-0.14699
C	5.190608	0.814685	0.100357
C	6.582752	0.787248	0.080825
C	7.088443	-0.50197	-0.00383
H	7.220462	1.66555	0.112809
S	-2.06846	1.814818	0.082159
S	5.786094	-1.69141	-0.05438
C	4.617218	-0.43829	0.033108
C	-3.35909	0.620157	-0.0243
C	-0.38716	-1.75161	-0.05885
C	4.124111	1.878555	0.157507
C	-6.73468	2.270868	-0.23752
C	-5.30874	2.264617	-0.2291
C	-4.73273	1.02902	-0.04929
S	-6.00397	-0.15756	0.150069
C	10.99425	-0.89593	-0.04179
C	10.417	-2.16408	-0.27458
C	9.012586	-2.15028	-0.27454
S	9.709472	0.292155	0.187023
C	-7.27702	1.014355	-0.05399
C	8.454695	-0.89862	-0.05124
C	12.37649	-0.72531	-0.0303
C	13.22207	0.353345	0.159019
H	12.86967	-1.67452	-0.21567
C	14.66544	0.334395	0.111932
C	15.50487	-0.73142	-0.14872
C	15.39837	4.423071	0.973867
C	16.51845	3.624158	0.771348
C	16.40561	2.267081	0.480629
C	15.13471	1.710705	0.393958
C	14.01951	2.528672	0.599434
C	14.12472	3.872834	0.887035
H	17.30476	1.684323	0.330982
H	13.23058	4.469402	1.039214
C	12.79294	1.722053	0.457321
C	16.92033	-0.62606	-0.17391
N	18.0758	-0.59337	-0.20648
C	15.05728	-2.0503	-0.4238
N	14.72858	-3.13598	-0.65119
O	11.65357	2.134828	0.573483
C	4.207425	2.810281	-1.05122
H	3.38692	3.535997	-1.0351
H	5.152133	3.365684	-1.04016

H	4.150105	2.245056	-1.98717
C	4.205322	2.679305	1.456667
H	3.382801	3.400475	1.515948
H	4.149517	2.019342	2.328481
H	5.148757	3.235037	1.503628
C	-0.45519	-2.55825	-1.35505
H	0.362651	-3.28589	-1.4009
H	-1.40189	-3.10757	-1.41267
H	-0.38372	-1.90244	-2.22892
C	-0.48809	-2.67814	1.152485
H	-0.43809	-2.10902	2.08655
H	-1.43645	-3.22729	1.133528
H	0.328273	-3.40892	1.148727
C	-7.26395	3.578456	-0.50728
H	-8.32029	3.810087	-0.58732
C	-6.30993	4.517204	-0.67562
C	10.95307	-3.45641	-0.52752
H	12.01083	-3.69732	-0.57293
C	9.988775	-4.39427	-0.71507
C	10.16771	-5.83972	-0.99759
H	9.712899	-6.11931	-1.9535
H	11.2318	-6.08248	-1.03984
H	9.704067	-6.45865	-0.2225
C	-6.47932	5.963309	-0.9639
H	-6.03538	6.584622	-0.17847
H	-6.00216	6.24447	-1.90892
H	-7.54226	6.208137	-1.03184
S	-4.6657	3.867456	-0.52335
S	8.360935	-3.74036	-0.59348
H	17.50736	4.065453	0.841342
H	15.52311	5.477131	1.199073
C	-8.66943	0.601024	-0.01114
C	-9.07606	-0.65835	-0.469
C	-9.66179	1.452098	0.491666
C	-10.4008	-1.04462	-0.44523
H	-8.33998	-1.33872	-0.88949
C	-10.989	1.072936	0.521083
H	-9.38186	2.414837	0.909115
C	-11.3896	-0.18189	0.044401
H	-10.6828	-2.01982	-0.82944
H	-11.7311	1.748596	0.93464
N	-12.7346	-0.56051	0.059004
C	-13.1039	-1.92681	0.169976
C	-14.0071	-2.4877	-0.73508
C	-12.5879	-2.72562	1.182358
C	-14.3783	-3.81171	-0.62581
H	-14.4188	-1.86896	-1.52761
C	-12.9397	-4.06541	1.287824

H	-11.8917	-2.29576	1.897226
C	-13.843	-4.61572	0.38255
H	-15.0794	-4.25862	-1.32355
H	-12.5144	-4.66253	2.086282
C	-13.7637	0.409532	-0.0577
C	-14.8477	0.396244	0.810293
C	-13.7207	1.378365	-1.06384
C	-15.8738	1.326026	0.691774
H	-14.8931	-0.35803	1.59094
C	-14.7241	2.317425	-1.17614
H	-12.8852	1.390341	-1.75793
C	-15.8117	2.300135	-0.30035
H	-16.7074	1.286986	1.383842
H	-14.6989	3.075921	-1.95215
O	-16.7453	3.259498	-0.49803
O	-14.2599	-5.90276	0.397939
C	-17.8578	3.28498	0.367934
H	-17.5532	3.444296	1.40988
H	-18.4805	4.119672	0.04734
H	-18.4404	2.35746	0.303008
C	-13.7429	-6.7523	1.397871
H	-14.1936	-7.73093	1.235734
H	-12.6519	-6.84272	1.324175
H	-14.0059	-6.39841	2.402507

Table S3: Cartesian coordinates of **ATTD3.**

**Atom**	**X-axis**	**Y-axis**	**Z-axis**
C	0.456422	-0.98066	-0.40025
C	-0.83451	-0.49166	-0.39732
C	-1.08171	0.896051	-0.47598
C	-0.03741	1.82085	-0.55636
C	1.250637	1.333283	-0.55579
C	1.49885	-0.05775	-0.47947
H	0.6568	-2.04835	-0.34031
H	-0.23796	2.888845	-0.61426
C	2.929901	-0.21655	-0.49363
C	3.562057	0.998874	-0.57322
C	4.961027	0.902787	-0.60101
H	5.64195	1.745019	-0.67942
C	-3.14441	-0.16576	-0.34602
C	-4.53298	-0.06697	-0.30753
C	-4.97493	1.246603	-0.37837
H	-5.21195	-0.91205	-0.24392
S	4.046099	-1.52954	-0.43686
S	-3.61708	2.367209	-0.49381
C	-2.51156	1.056318	-0.44529
C	5.398549	-0.40552	-0.53697
C	2.552423	2.117118	-0.62526

C	-2.13142	-1.28117	-0.3127
C	8.673282	-2.25656	-0.64042
C	7.253882	-2.1548	-0.72218
C	6.746868	-0.89319	-0.517
S	8.074541	0.195722	-0.1798
C	-8.84684	1.861525	-0.15819
C	-8.22138	3.080615	-0.50445
C	-6.8239	2.984924	-0.60477
S	-7.61238	0.617047	0.051792
C	9.280993	-1.04889	-0.35761
C	-6.31821	1.71614	-0.35316
C	-10.2305	1.769864	-0.03652
C	-11.1131	0.752756	0.279955
H	-10.6908	2.726124	-0.26557
C	-12.5582	0.857602	0.368462
C	-13.3175	2.008494	0.368046
C	-14.0498	-2.60943	0.948043
C	-14.3052	-1.25542	0.608498
C	-13.0773	-0.5096	0.595521
C	-12.0054	-1.32519	0.827879
C	-10.7301	-0.61402	0.629118
C	-14.695	2.052852	0.702302
N	-15.8082	2.160656	0.995434
C	-12.7886	3.309299	0.145577
N	-12.4059	4.384492	-0.03973
O	-9.62013	-1.09529	0.732485
C	-2.29271	-2.2138	-1.51289
H	-1.50695	-2.97697	-1.51399
H	-3.26199	-2.72355	-1.47232
H	-2.23425	-1.65714	-2.45389
C	-2.21714	-2.07066	0.993253
H	-1.43251	-2.83398	1.034087
H	-2.1016	-1.41055	1.859026
H	-3.18662	-2.57586	1.070734
C	2.659454	2.899187	-1.93422
H	1.879133	3.666217	-1.99108
H	3.632704	3.398839	-2.00123
H	2.553551	2.234015	-2.79745
C	2.70027	3.056386	0.57111
H	2.623831	2.505638	1.514373
H	3.674166	3.558297	0.54272
H	1.920562	3.825874	0.556089
C	9.133809	-3.58473	-0.9368
H	10.17545	-3.88454	-0.96319
C	8.134303	-4.44894	-1.20765
C	-8.70345	4.388145	-0.78442
H	-9.74558	4.690578	-0.75742
C	-7.70609	5.257667	-1.09084

C	-7.82697	6.696379	-1.43239
H	-7.43419	6.903647	-2.43313
H	-8.87602	6.999151	-1.40595
H	-7.26996	7.322306	-0.72772
C	8.228797	-5.88958	-1.55274
H	7.692155	-6.51245	-0.82888
H	7.800077	-6.09637	-2.53926
H	9.275573	-6.20339	-1.56271
S	6.52981	-3.69792	-1.12889
S	-6.11263	4.516972	-1.05453
C	10.68955	-0.74357	-0.17393
C	11.22567	0.508864	-0.50006
C	11.56641	-1.7003	0.352995
C	12.56502	0.789359	-0.32209
H	10.58348	1.270108	-0.93568
C	12.90741	-1.42827	0.533497
H	11.17794	-2.66382	0.669512
C	13.43753	-0.17668	0.194874
H	12.95343	1.763642	-0.60197
H	13.55637	-2.18517	0.96255
N	14.79591	0.101642	0.368847
C	15.23495	1.417488	0.670522
C	16.28208	1.992484	-0.05242
C	14.65426	2.146667	1.700691
C	16.73336	3.25941	0.254098
H	16.74436	1.429632	-0.85844
C	15.0875	3.431736	2.002279
H	13.84522	1.705662	2.276194
C	16.13733	3.993969	1.281321
H	17.54818	3.715245	-0.29953
H	14.60811	3.973133	2.809213
C	15.76491	-0.93185	0.292275
C	16.73425	-1.06604	1.277463
C	15.78093	-1.81565	-0.79037
C	17.70829	-2.05411	1.196566
H	16.73231	-0.38159	2.121149
C	16.72934	-2.81374	-0.86681
H	15.03589	-1.71321	-1.57437
C	17.70488	-2.94124	0.124314
H	18.45474	-2.12833	1.979326
H	16.74925	-3.50622	-1.70243
O	18.5954	-3.94557	-0.04699
O	16.64678	5.228668	1.497894
C	19.59122	-4.11607	0.936764
H	19.15278	-4.32654	1.920267
H	20.19237	-4.96937	0.624332
H	20.23617	-3.23182	1.013912
C	16.08619	6.004239	2.533885

H	16.63653	6.944486	2.548065
H	15.02452	6.212925	2.351634
H	16.19242	5.511308	3.508391
C	-15.0581	-3.56241	1.028036
H	-14.8494	-4.59783	1.271525
C	-15.6267	-0.90941	0.289963
H	-15.9046	0.094024	0.001121
C	-16.6173	-1.85338	0.365843
C	-16.3427	-3.17668	0.737429
S	-12.3537	-2.96066	1.162442
N	-17.9977	-1.37259	0.172406
O	-18.1856	-0.58006	-0.72181
O	-18.8288	-1.76979	0.956658
N	-17.3603	-4.23615	0.669294
O	-18.1739	-4.15911	-0.22375
O	-17.2807	-5.13128	1.480468

Table S4: Cartesian coordinates of **ATTD4.**

Atom	X-axis	Y-axis	Z-axis
C	0.116887	-1.02876	0.153791
C	-1.17577	-0.54446	0.133566
C	-1.42754	0.839265	0.012643
C	-0.38601	1.764495	-0.08919
C	0.903994	1.281166	-0.07073
C	1.156841	-0.10548	0.049615
H	0.32002	-2.09382	0.244795
H	-0.58962	2.829453	-0.18239
C	2.588837	-0.26012	0.032192
C	3.216376	0.953405	-0.09585
C	4.614758	0.859893	-0.14517
H	5.290491	1.701808	-0.26231
C	-3.48787	-0.22748	0.137852
C	-4.87865	-0.13594	0.138381
C	-5.32567	1.171095	0.017603
H	-5.55402	-0.98283	0.214651
S	3.709939	-1.56758	0.111504
S	-3.97121	2.295771	-0.0956
C	-2.85914	0.993537	0.01782
C	5.058295	-0.44457	-0.04898
C	2.202719	2.066688	-0.17179
C	-2.4708	-1.33647	0.224537
C	8.334814	-2.2921	-0.18511
C	6.911865	-2.20153	-0.18304
C	6.408225	-0.92805	-0.05774
S	7.746694	0.189702	0.096107
C	-9.21007	1.753175	0.035527
C	-8.57865	2.989763	-0.20807
C	-7.17498	2.907703	-0.2341

S	-7.98131	0.506181	0.245534
C	8.949457	-1.06274	-0.05125
C	-6.67347	1.632938	-0.01629
C	-10.6025	1.645484	0.072482
C	-11.4891	0.608541	0.267414
H	-11.0578	2.611352	-0.12443
C	-12.9408	0.695178	0.270902
C	-13.7103	1.837431	0.341467
C	-14.4376	-2.80921	0.371091
C	-14.6755	-1.42847	0.158502
C	-13.4541	-0.68664	0.323638
C	-12.3938	-1.51688	0.561572
C	-11.1192	-0.78663	0.526584
C	-15.1022	1.842492	0.616385
N	-16.2267	1.918475	0.874209
C	-13.1786	3.154557	0.277579
N	-12.7904	4.242131	0.218176
O	-10.0107	-1.26192	0.669007
C	-2.60968	-2.31034	-0.94492
H	-1.82213	-3.07083	-0.90709
H	-3.57816	-2.82151	-0.90269
H	-2.53735	-1.78596	-1.90337
C	-2.5741	-2.08067	1.555684
H	-1.78812	-2.83968	1.635186
H	-2.47365	-1.38997	2.399266
H	-3.54346	-2.58573	1.636267
C	2.297983	2.811389	-1.50318
H	1.517107	3.576573	-1.57567
H	3.270412	3.309149	-1.59285
H	2.185095	2.121656	-2.34605
C	2.356098	3.039898	0.996335
H	2.287504	2.515776	1.955254
H	3.328317	3.543507	0.947123
H	1.573817	3.806315	0.964687
C	8.786491	-3.63994	-0.39283
H	9.827265	-3.93696	-0.45867
C	7.779216	-4.52766	-0.52073
C	-9.05474	4.31091	-0.43843
H	-10.0994	4.605599	-0.44976
C	-8.0502	5.201604	-0.63668
C	-8.16259	6.657213	-0.90161
H	-7.72316	6.922254	-1.86885
H	-9.21339	6.955194	-0.90919
H	-7.64402	7.244081	-0.13651
C	7.863541	-5.99316	-0.74143
H	7.376018	-6.55017	0.066066
H	7.378402	-6.28833	-1.67809
H	8.910224	-6.30436	-0.786

S	6.176785	-3.77632	-0.40596
S	-6.45318	4.46815	-0.55331
C	10.3646	-0.7344	-0.01212
C	10.85532	0.475228	-0.51883
C	11.29619	-1.62473	0.53708
C	12.20219	0.775707	-0.49874
H	10.16765	1.183157	-0.9744
C	12.64469	-1.33259	0.561975
H	10.95133	-2.54783	0.993396
C	13.12953	-0.12777	0.036379
H	12.54984	1.713263	-0.92088
H	13.33826	-2.0365	1.010847
N	14.49668	0.156651	0.046519
C	14.97122	1.494113	0.056845
C	15.94413	1.900825	-0.85887
C	14.50113	2.415766	0.983929
C	16.42961	3.191825	-0.84193
H	16.32084	1.187505	-1.58646
C	14.96765	3.724251	0.993697
H	13.75241	2.10913	1.70894
C	15.94148	4.119098	0.080052
H	17.18643	3.51663	-1.54906
H	14.57458	4.418354	1.727397
C	15.45354	-0.89228	-0.00334
C	16.49382	-0.93996	0.914703
C	15.38432	-1.87423	-0.99454
C	17.45633	-1.94095	0.856583
H	16.55685	-0.1765	1.685245
C	16.32345	-2.88245	-1.04847
H	14.58058	-1.83933	-1.72471
C	17.37044	-2.92425	-0.12481
H	18.25852	-1.94908	1.585854
H	16.27971	-3.65133	-1.81333
O	18.2436	-3.94723	-0.26844
O	16.47093	5.362361	0.008478
C	19.30873	-4.03976	0.651153
H	18.94328	-4.16344	1.678196
H	19.88475	-4.92068	0.369524
H	19.95896	-3.15721	0.605566
C	16.00149	6.333428	0.917177
H	16.54123	7.252959	0.69266
H	14.92528	6.509918	0.796624
H	16.20248	6.043888	1.956217
C	-15.4423	-3.76022	0.263927
H	-15.2444	-4.81169	0.438655
C	-15.9618	-1.04977	-0.24273
H	-16.2013	-0.02239	-0.47478
C	-16.9597	-1.99196	-0.35866

C	-16.7087	-3.34833	-0.0934
S	-12.7561	-3.17993	0.698036
Cl	-17.9638	-4.53564	-0.23777
Cl	-18.5399	-1.46414	-0.84749

Table S5: Cartesian coordinates of **ATTD5.**

Atom	X-axis	Y-axis	Z-axis
C	0.991879	-1.00829	-0.22681
C	-0.29273	-0.50253	-0.21442
C	-0.52014	0.890271	-0.24209
C	0.536392	1.803169	-0.28631
C	1.817826	1.29877	-0.29788
C	2.047261	-0.09718	-0.26618
H	1.178054	-2.08	-0.2037
H	0.349902	2.875079	-0.30736
C	3.476233	-0.27388	-0.27754
C	4.124847	0.934783	-0.31587
C	5.522254	0.822288	-0.31741
H	6.211649	1.660854	-0.34487
C	-2.59943	-0.14412	-0.16135
C	-3.98693	-0.02373	-0.11423
C	-4.40726	1.298964	-0.12256
H	-4.67889	-0.85972	-0.07378
S	4.575352	-1.60137	-0.23989
S	-3.02977	2.399749	-0.19353
C	-1.94713	1.070155	-0.20742
C	5.944784	-0.49284	-0.27649
C	3.130017	2.067221	-0.33569
C	-1.60236	-1.27512	-0.16821
C	9.191421	-2.39667	-0.19835
C	7.770258	-2.28372	-0.22142
C	7.286934	-0.99621	-0.25098
S	8.644229	0.109973	-0.23301
C	-8.2707	1.986501	0.065745
C	-7.60138	3.226247	-0.0134
C	-6.2034	3.104384	-0.08831
S	-7.0819	0.682755	0.043406
C	9.825747	-1.17104	-0.20614
C	-5.74115	1.795349	-0.07093
C	-9.65986	1.921442	0.16849
C	-10.581	0.898934	0.276925
H	-10.083	2.920489	0.139508
C	-12.0187	1.041295	0.429568
C	-12.7192	2.188993	0.735693
C	-13.677	-2.38351	0.19302
C	-13.8649	-0.98366	0.20828
C	-12.598	-0.31481	0.336288
C	-11.5647	-1.20967	0.335255

C	-10.2604	-0.53009	0.283814
C	-14.081	2.208892	1.132756
N	-15.1743	2.294105	1.499032
C	-12.1306	3.481196	0.810673
N	-11.6971	4.551419	0.87072
O	-9.17125	-1.06572	0.240656
C	-1.77011	-2.15493	-1.40697
H	-0.99519	-2.92863	-1.4367
H	-2.7469	-2.65164	-1.3926
H	-1.69893	-1.56009	-2.32343
C	-1.70731	-2.11642	1.103324
H	-0.93203	-2.89016	1.11792
H	-1.59027	-1.49411	1.996453
H	-2.68323	-2.61258	1.153256
C	3.252902	2.892847	-1.61602
H	2.481727	3.670397	-1.64977
H	4.232304	3.382868	-1.66084
H	3.143634	2.259581	-2.50248
C	3.28493	2.962371	0.893407
H	3.198888	2.379563	1.816363
H	4.264782	3.453385	0.88591
H	2.514495	3.741237	0.903638
C	9.623331	-3.76615	-0.22276
H	10.66034	-4.08302	-0.23771
C	8.602981	-4.64763	-0.24395
C	-8.03471	4.581073	-0.01751
H	-9.06794	4.908002	0.043652
C	-7.00259	5.459435	-0.0945
C	-7.06894	6.941435	-0.11902
H	-6.62592	7.346315	-1.03484
H	-8.10971	7.268666	-0.06705
H	-6.52794	7.379609	0.726047
C	8.664891	-6.13036	-0.27369
H	8.167846	-6.57162	0.59711
H	8.176552	-6.53604	-1.16639
H	9.706889	-6.45979	-0.27458
S	7.011809	-3.86307	-0.24306
S	-5.4324	4.671161	-0.16704
C	11.24466	-0.86025	-0.16656
C	11.7711	0.267029	-0.80954
C	12.14008	-1.67663	0.535632
C	13.11798	0.562361	-0.76248
H	11.11203	0.910214	-1.38722
C	13.48907	-1.38753	0.590333
H	11.76253	-2.53153	1.08916
C	14.00848	-0.26019	-0.05927
H	13.49642	1.434474	-1.28642
H	14.15198	-2.02815	1.16333

N	15.3705	0.042408	-0.00524
C	15.8189	1.388412	-0.07862
C	16.82517	1.743882	-0.97871
C	15.28961	2.365808	0.75425
C	17.28738	3.04225	-1.03745
H	17.24574	0.984644	-1.63226
C	15.73362	3.680466	0.687891
H	14.51254	2.09769	1.464661
C	16.74243	4.024526	-0.20804
H	18.07042	3.329782	-1.73199
H	15.29684	4.420366	1.348468
C	16.34109	-0.97413	0.192342
C	17.31702	-0.83467	1.170282
C	16.35491	-2.11632	-0.61232
C	18.29596	-1.80394	1.350249
H	17.31662	0.052069	1.798091
C	17.31047	-3.09362	-0.42666
H	15.60362	-2.23259	-1.38836
C	18.29317	-2.94563	0.554729
H	19.04605	-1.65998	2.119027
H	17.33004	-3.98467	-1.04627
O	19.19206	-3.95253	0.652622
O	17.25529	5.268867	-0.34602
C	20.19874	-3.84283	1.634296
H	19.7721	-3.79226	2.643846
H	20.81142	-4.73987	1.55073
H	20.82919	-2.96066	1.467134
C	16.74311	6.288717	0.48273
H	17.28331	7.198571	0.222963
H	15.67083	6.445816	0.311238
H	16.90664	6.064146	1.54415
C	-14.7404	-3.26901	0.080168
H	-14.5636	-4.33783	0.086841
C	-15.1715	-0.51491	0.020746
H	-15.3652	0.545691	-0.03279
C	-16.2355	-1.38396	-0.09718
C	-16.0236	-2.78235	-0.05895
S	-11.9967	-2.85983	0.270487
C	-17.1342	-3.79961	-0.1206
C	-17.5917	-0.76241	-0.31714
F	-16.6597	-5.02232	-0.37382
F	-18.0248	-3.52842	-1.07439
F	-17.793	-3.87881	1.038354
F	-17.9804	-0.86199	-1.59194
F	-18.5347	-1.32978	0.437048
F	-17.5901	0.537026	-0.01852

Table S6: Cartesian coordinates of **ATTD6.**

Atom	X-axis	Y-axis	Z-axis
C	-1.38674	-0.84903	0.381327
C	-0.10479	-0.33691	0.369936
C	0.116917	1.055944	0.43008
C	-0.94361	1.962778	0.500365
C	-2.22265	1.452109	0.508582
C	-2.44552	0.055928	0.450369
H	-1.56811	-1.92083	0.335652
H	-0.76197	3.034834	0.544133
C	-3.87329	-0.12899	0.469608
C	-4.52775	1.075215	0.535278
C	-5.92478	0.953289	0.566125
H	-6.62094	1.783867	0.634325
C	2.199144	0.031759	0.311222
C	3.585821	0.157149	0.270515
C	4.002218	1.480121	0.323022
H	4.281266	-0.6752	0.218393
S	-4.96438	-1.4632	0.431103
S	2.622494	2.575164	0.423976
C	1.543246	1.242546	0.394802
C	-6.33798	-0.36389	0.518391
C	-3.53871	2.21247	0.570622
C	1.206927	-1.10282	0.294121
C	-9.5751	-2.27947	0.634367
C	-8.15774	-2.15008	0.710352
C	-7.67667	-0.87802	0.505478
S	-9.02778	0.185113	0.176769
C	7.862493	2.168138	0.112891
C	7.211632	3.379799	0.435421
C	5.815551	3.259055	0.527296
S	6.654271	0.897403	-0.08846
C	-10.2079	-1.08405	0.356452
C	5.336144	1.976363	0.294182
C	9.249179	2.099309	0.012645
C	10.15411	1.095333	-0.28007
H	9.690242	3.065236	0.23979
C	11.59773	1.227134	-0.34029
C	12.34173	2.388096	-0.33953
C	13.17624	-2.19963	-0.89186
C	13.38546	-0.84885	-0.53104
C	12.14402	-0.12963	-0.5513
C	11.09693	-0.96495	-0.82013
C	9.803247	-0.27844	-0.64126
C	13.72095	2.441875	-0.66818
N	14.83402	2.548691	-0.96241
C	11.7921	3.682801	-0.13425
N	11.38898	4.752555	0.038618
O	8.704131	-0.77838	-0.76608

C	1.38717	-2.0156	1.50691
H	0.615688	-2.79309	1.520428
H	2.365681	-2.50782	1.471783
H	1.320021	-1.44689	2.440121
C	1.305944	-1.90835	-1.00101
H	0.535023	-2.68599	-1.03076
H	1.17791	-1.26226	-1.87554
H	2.284264	-2.39708	-1.07255
C	-3.65767	3.010012	1.869125
H	-2.89118	3.791622	1.914257
H	-4.63965	3.492914	1.931244
H	-3.5383	2.358586	2.741008
C	-3.70632	3.13258	-0.63809
H	-3.62224	2.5704	-1.57394
H	-4.68911	3.617092	-0.61407
H	-2.94069	3.916209	-0.63589
C	-10.0091	-3.61665	0.930377
H	-11.0449	-3.93586	0.961046
C	-8.9923	-4.46212	1.195132
C	7.667311	4.700336	0.69856
H	8.704072	5.021139	0.674526
C	6.65168	5.555757	0.983374
C	6.743081	7.001945	1.302074
H	6.335912	7.218771	2.294979
H	7.786735	7.323263	1.281459
H	6.182701	7.605963	0.58113
C	-9.05747	-5.90461	1.539354
H	-8.51317	-6.51667	0.812037
H	-8.61944	-6.1039	2.523328
H	-10.098	-6.23833	1.554953
S	-7.40276	-3.68039	1.109868
S	5.072411	4.7849	0.946372
C	-11.6239	-0.80914	0.180378
C	-12.1888	0.424878	0.526415
C	-12.479	-1.77943	-0.35742
C	-13.5357	0.674534	0.358617
H	-11.5633	1.195308	0.970099
C	-13.8271	-1.53803	-0.52851
H	-12.0682	-2.72855	-0.68906
C	-14.3867	-0.3055	-0.16842
H	-13.9467	1.634596	0.654913
H	-14.4589	-2.30467	-0.96579
N	-15.7525	-0.05928	-0.33103
C	-16.2281	1.24908	-0.6102
C	-17.2877	1.782532	0.126537
C	-15.675	2.008829	-1.63322
C	-17.7798	3.038468	-0.16136
H	-17.7275	1.195203	0.927605

C	-16.1498	3.283574	-1.9163
H	-14.856	1.599858	-2.2182
C	-17.213	3.80306	-1.18302
H	-18.6052	3.462036	0.402176
H	-15.6936	3.851415	-2.71875
C	-16.6948	-1.11852	-0.27321
C	-17.6636	-1.25595	-1.25844
C	-16.6869	-2.02414	0.791458
C	-18.615	-2.26708	-1.19445
H	-17.6801	-0.5547	-2.08807
C	-17.6122	-3.04482	0.850534
H	-15.9423	-1.92017	1.575741
C	-18.5885	-3.1744	-0.13955
H	-19.3619	-2.34261	-1.97665
H	-17.6138	-3.75387	1.672397
O	-19.4572	-4.20019	0.016164
O	-17.7625	5.02332	-1.38262
C	-20.4564	-4.3707	-0.96412
H	-20.0212	-4.55674	-1.95397
H	-21.0407	-5.23957	-0.66277
H	-21.1171	-3.49667	-1.0215
C	-17.2338	5.825467	-2.41493
H	-17.8099	6.750206	-2.41374
H	-16.1765	6.061049	-2.24041
H	-17.3359	5.340045	-3.39365
C	14.21594	-3.12003	-0.95105
H	14.02948	-4.14968	-1.23655
C	14.68289	-0.48354	-0.15213
H	14.8935	0.518243	0.194439
C	15.71632	-1.39424	-0.19271
C	15.49566	-2.72447	-0.62344
S	11.49628	-2.58938	-1.16159
S	16.65396	-4.09638	-0.52644
O	16.95393	-4.32994	0.867008
O	16.132	-5.15308	-1.34908
O	17.96258	-3.58557	-1.27876
H	18.52165	-3.07504	-0.66175
S	17.30526	-0.62052	0.090205
O	17.95686	-0.40522	-1.17183
O	17.12832	0.441729	1.042612
O	18.18782	-1.79336	0.76615
H	17.93078	-1.99047	1.681208

Table S7: Cartesian coordinates of **ATTD7.**

Atom	X-axis	Y-axis	Z-axis
C	0.101856	-1.03693	-0.04097
C	1.3965	-0.55828	-0.03027
C	1.654513	0.827115	0.055888

C	0.616698	1.759833	0.130953
C	-0.67517	1.28237	0.12077
C	-0.93382	-0.10632	0.036344
H	-0.10632	-2.10289	-0.10522
H	0.825252	2.825842	0.196639
C	-2.36573	-0.25457	0.051701
C	-2.98911	0.964616	0.143469
C	-4.38799	0.877752	0.186392
H	-5.0613	1.724979	0.2759
C	3.709296	-0.25338	-0.04091
C	5.0989	-0.16897	-0.04366
C	5.553118	1.139306	0.045565
H	5.769748	-1.02121	-0.09652
S	-3.49131	-1.55961	0.001072
S	4.204235	2.273523	0.131077
C	3.086204	0.974548	0.049044
C	-4.83576	-0.42753	0.122398
C	-1.97119	2.075564	0.194029
C	2.687121	-1.35912	-0.09877
C	-8.11771	-2.26285	0.275498
C	-6.6949	-2.17475	0.290478
C	-6.18699	-0.90654	0.131599
S	-7.52131	0.208261	-0.07531
C	9.440298	1.6904	-0.00518
C	8.818997	2.932871	0.251916
C	7.416515	2.86175	0.287211
S	8.197353	0.455128	-0.21785
C	-8.72792	-1.03729	0.094301
C	6.902534	1.590689	0.066864
C	10.82697	1.569483	-0.04767
C	11.70371	0.521324	-0.25959
H	11.29455	2.527623	0.158127
C	13.15367	0.592579	-0.26892
C	13.94031	1.724078	-0.30989
C	14.59957	-2.93259	-0.49216
C	14.86189	-1.56092	-0.23359
C	13.65341	-0.79528	-0.36965
C	12.58199	-1.60385	-0.62476
C	11.31371	-0.85695	-0.55446
C	15.32933	1.708931	-0.59629
N	16.45326	1.761818	-0.86186
C	13.4305	3.046717	-0.20145
N	13.05818	4.137129	-0.10565
O	10.20173	-1.32137	-0.70273
C	2.820205	-2.30102	1.097528
H	2.028052	-3.0574	1.080556
H	3.785514	-2.81909	1.069589
H	2.750999	-1.75	2.041118

C	2.787523	-2.1407	-1.40847
H	1.996241	-2.89593	-1.46784
H	2.693409	-1.47359	-2.27149
H	3.753286	-2.65482	-1.47276
C	-2.06967	2.85809	1.503226
H	-1.28432	3.62008	1.558368
H	-3.03939	3.364237	1.572679
H	-1.96613	2.192206	2.366187
C	-2.11488	3.016155	-1.00193
H	-2.0445	2.464941	-1.94539
H	-3.08505	3.525126	-0.97138
H	-1.32931	3.779715	-0.98837
C	-8.57545	-3.60223	0.521216
H	-9.61768	-3.89532	0.581592
C	-7.5721	-4.48682	0.693687
C	9.307617	4.24638	0.490712
H	10.3547	4.53259	0.498413
C	8.310693	5.143816	0.703959
C	8.437229	6.596074	0.980053
H	8.005416	6.856823	1.951825
H	9.490539	6.884893	0.98407
H	7.919375	7.193341	0.222616
C	-7.66238	-5.94369	0.963539
H	-7.16388	-6.52898	0.183244
H	-7.19171	-6.20674	1.917011
H	-8.71016	-6.25175	1.003582
S	-5.96657	-3.74246	0.576278
S	6.709037	4.423668	0.625672
C	-10.1421	-0.7107	0.023614
C	-10.6426	0.511072	0.489949
C	-11.0634	-1.61699	-0.51676
C	-11.9896	0.807616	0.440845
H	-9.96307	1.232126	0.937157
C	-12.4116	-1.32884	-0.57115
H	-10.7094	-2.55105	-0.94282
C	-12.9071	-0.11184	-0.08427
H	-12.3456	1.755034	0.832825
H	-13.0965	-2.04582	-1.01251
N	-14.2742	0.168629	-0.12238
C	-14.7516	1.504843	-0.15947
C	-15.7306	1.925418	0.743235
C	-14.278	2.410841	-1.09989
C	-16.2185	3.214941	0.700825
H	-16.1103	1.224189	1.48097
C	-14.7473	3.717876	-1.13574
H	-13.5242	2.092704	-1.81464
C	-15.7271	4.126905	-0.23471
H	-16.98	3.550685	1.397744

H	-14.3511	4.399086	-1.87979
C	-15.2305	-0.88072	-0.06872
C	-16.2565	-0.94486	-1.0016
C	-15.1766	-1.84495	0.94077
C	-17.2195	-1.94525	-0.94109
H	-16.3079	-0.19484	-1.78609
C	-16.1159	-2.85292	0.997333
H	-14.385	-1.79641	1.683342
C	-17.1481	-2.91169	0.058139
H	-18.0104	-1.96642	-1.68228
H	-16.0838	-3.6083	1.776098
O	-18.0227	-3.9328	0.205557
O	-16.2597	5.369831	-0.18808
C	-19.0732	-4.04191	-0.72905
H	-18.6914	-4.18191	-1.74799
H	-19.6526	-4.91881	-0.44202
H	-19.7248	-3.15945	-0.7081
C	-15.7885	6.326114	-1.1116
H	-16.3321	7.247773	-0.90602
H	-14.7135	6.508221	-0.98883
H	-15.9836	6.017749	-2.14631
C	15.58546	-3.90715	-0.43183
H	15.36144	-4.94469	-0.65206
C	16.15484	-1.21678	0.181148
H	16.40633	-0.20103	0.45131
C	17.13982	-2.18314	0.263629
C	16.86476	-3.53455	-0.06207
S	12.91545	-3.2656	-0.8171
C	18.44881	-1.81133	0.691865
N	19.5083	-1.51685	1.039679
C	17.89181	-4.51916	0.014025
N	18.72035	-5.31956	0.073742

Table S8: Cartesian coordinates of **ATTD8.**

Atom	X-axis	Y-axis	Z-axis
C	-0.93259	-0.95893	0.477394
C	0.357253	-0.46669	0.471895
C	0.599775	0.923328	0.508118
C	-0.44732	1.846784	0.55115
C	-1.73452	1.355727	0.554192
C	-1.97832	-0.0371	0.5172
H	-1.12975	-2.02856	0.448577
H	-0.24995	2.916622	0.577847
C	-3.40964	-0.199	0.520209
C	-4.04556	1.016144	0.557794
C	-5.44475	0.917158	0.567892
H	-6.12786	1.760247	0.610758

C	2.667367	-0.13389	0.428671
C	4.057617	-0.03087	0.393873
C	4.493401	1.285133	0.419098
H	4.740331	-0.87476	0.361917
S	-4.52188	-1.51569	0.483192
S	3.13083	2.403306	0.487085
C	2.029977	1.087049	0.481715
C	-5.87897	-0.39334	0.531771
C	-3.03881	2.137929	0.587709
C	1.658052	-1.25358	0.425187
C	-9.14709	-2.25825	0.594469
C	-7.72893	-2.15137	0.690005
C	-7.22571	-0.8854	0.502465
S	-8.5565	0.202048	0.169509
C	8.361314	1.924455	0.174252
C	7.726727	3.150211	0.460823
C	6.328712	3.046105	0.569735
S	7.142062	0.658439	0.035582
C	-9.75782	-1.05064	0.321372
C	5.835483	1.764165	0.378037
C	9.747762	1.84822	0.020711
C	10.63889	0.834927	-0.25831
H	10.19434	2.824795	0.179342
C	12.07806	0.964369	-0.42388
C	12.79937	2.127015	-0.59418
C	13.66282	-2.49258	-0.68306
C	13.87869	-1.10345	-0.50804
C	12.62296	-0.40264	-0.52531
C	11.56891	-1.26666	-0.63257
C	10.28395	-0.57346	-0.46185
C	14.15115	2.173511	-1.02445
N	15.23441	2.292301	-1.41055
C	12.23851	3.429136	-0.48583
N	11.82796	4.506381	-0.39594
O	9.180878	-1.08153	-0.47364
C	1.811989	-2.14333	1.658575
H	1.028233	-2.90834	1.680641
H	2.783049	-2.65118	1.643913
H	1.744634	-1.55382	2.578764
C	1.757897	-2.08791	-0.8514
H	0.974813	-2.85361	-0.87332
H	1.650023	-1.45865	-1.74083
H	2.728919	-2.5936	-0.90169
C	-3.16041	2.958521	1.871478
H	-2.38071	3.727117	1.91386
H	-4.13444	3.459477	1.912917
H	-3.0638	2.319562	2.755339
C	-3.17685	3.040871	-0.63757

H	-3.0911	2.461994	-1.56302
H	-4.15162	3.541972	-0.6334
H	-2.39826	3.811672	-0.6389
C	-9.60568	-3.59151	0.869363
H	-10.6468	-3.89437	0.881176
C	-8.60554	-4.4553	1.138673
C	8.195698	4.477212	0.669975
H	9.234712	4.787732	0.622453
C	7.191435	5.351028	0.933568
C	7.298029	6.806598	1.201127
H	6.90171	7.061278	2.189533
H	8.344192	7.117889	1.160308
H	6.736448	7.390624	0.464709
C	-8.69766	-5.90034	1.465644
H	-8.15363	-6.5128	0.738439
H	-8.2756	-6.11754	2.45281
H	-9.74345	-6.21761	1.46411
S	-7.00302	-3.69747	1.083629
S	5.603272	4.593901	0.940065
C	-11.1674	-0.75415	0.12894
C	-11.7258	0.475817	0.497884
C	-12.0224	-1.70167	-0.44814
C	-13.0677	0.743003	0.315394
H	-11.1001	1.227547	0.972366
C	-13.3644	-1.44156	-0.63679
H	-11.6147	-2.64683	-0.79484
C	-13.9185	-0.21344	-0.25297
H	-13.4737	1.698254	0.632666
H	-13.9965	-2.1899	-1.10453
N	-15.2809	0.043647	-0.43132
C	-15.7598	1.362716	-0.64047
C	-16.8577	1.833203	0.083871
C	-15.173	2.200457	-1.58093
C	-17.3525	3.10208	-0.13437
H	-17.3259	1.185079	0.81924
C	-15.6501	3.488462	-1.79053
H	-14.3248	1.843968	-2.15839
C	-16.7505	3.944746	-1.07038
H	-18.2073	3.474975	0.421046
H	-15.1661	4.117686	-2.52856
C	-16.2261	-1.01478	-0.38617
C	-17.1841	-1.1477	-1.38234
C	-16.2316	-1.92171	0.676959
C	-18.1389	-2.15609	-1.33011
H	-17.1895	-0.44456	-2.21054
C	-17.1603	-2.93992	0.724881
H	-15.4944	-1.82072	1.468525
C	-18.1262	-3.06484	-0.27588

H	-18.8777	-2.22888	-2.1203
H	-17.1727	-3.65058	1.545229
O	-18.999	-4.08834	-0.13091
O	-17.3041	5.17247	-1.20565
C	-19.9895	-4.25437	-1.12082
H	-19.5457	-4.44096	-2.10668
H	-20.5796	-5.12151	-0.82583
H	-20.6466	-3.37794	-1.1834
C	-16.7397	6.049623	-2.15452
H	-17.3258	6.967283	-2.11466
H	-15.6939	6.281468	-1.9171
H	-16.793	5.633427	-3.16834
C	14.70876	-3.40782	-0.70364
H	14.52417	-4.46606	-0.85971
C	15.18981	-0.68277	-0.2523
H	15.40648	0.348566	-0.01525
C	16.23476	-1.58691	-0.26214
C	15.99742	-2.95172	-0.52556
S	11.96962	-2.91837	-0.80369
C	17.59632	-1.15002	0.148885
O	18.43711	-1.89013	0.594937
O	17.76488	0.161499	0.000732
C	17.09847	-3.9554	-0.61688
O	17.14508	-4.96238	0.037245
O	17.97131	-3.62274	-1.56482
C	19.02076	0.683063	0.435342
H	19.15806	0.508182	1.504905
H	19.83972	0.21257	-0.11292
H	18.98819	1.75019	0.226625
C	19.14914	-4.42713	-1.61937
H	19.71335	-4.30848	-0.69019
H	18.89583	-5.47978	-1.76044
H	19.72871	-4.05804	-2.46294

Table S9: Calculated energies (E) and energy gap (ΔE) of HOMO-1, LUMO+1, HOMO-2 and LUMO+2 for **ATTR1** and **ATTD2-ATTD8** at **M06/6-31G (d,p)**.

Compounds	HOMO-1	LUMO+1	ΔE	HOMO-2	LUMO+2	ΔE
**ATTR1**	-6.071	-3.217	2.854	-6.713	-2.604	4.109
**ATTD2**	-5.588	-2.517	3.071	-5.95	-2.034	3.916
**ATTD3**	-5.629	-3.27	2.359	-6.009	-2.836	3.173
**ATTD4**	-5.586	-2.957	2.629	-5.948	-2.033	3.915
**ATTD5**	-5.604	-3.047	2.557	-5.974	-2.074	3.9
**ATTD6**	-5.624	-3.169	2.455	-6.004	-2.204	3.8
**ATTD7**	-5.631	-3.177	2.454	-6.003	-2.281	3.722
**ATTD8**	-5.575	-2.959	2.616	-5.943	-2.040	3.903

E=energy, ΔE (eV) =E_{LUMO}-E_{HOMO}; HOMO=highest occupied molecular orbital; LUMO=lowest unoccupied molecular orbital, MO=molecular orbital, Units in eV

Table S10: Wavelength, excitation energy and oscillator strength of **ATTR1** in chloroform solvent at M06/6-311G (d,p) level.

NO	DFT λ (*nm*)	*E*(*eV*)	f_{os}	MO contributions
1	751.510	1.650	3.297	H→L (93%), H-1→L+1 (5%)
2	620.666	1.998	0.003	H→L+1 (90%), H-1→L (8%)
3	559.394	2.216	0.002	H-1→L (88%), H-2→L+1 (3%), H→L+1 (8%)
4	507.176	2.445	0.053	H-1→L+1 (54%), H→L+2 (32%), H-2→L (3%), H-1→L+3 (6%), H→L (2%)
5	498.429	2.488	0.272	H-1→L+1 (35%), H→L+2 (50%), H-1→L+3 (8%), H→L (3%)
6	491.708	2.522	0.001	H-1→L+2 (16%), H→L+3 (79%)

MO=molecular orbital, H=HOMO, L=LUMO, f_{os}= oscillator strength, wavelength= λ (*nm*)

Table S11: Wavelength, excitation energy and oscillator strength of **ATTD2** in chloroform solvent at M06/6-311G (d,p) level.

NO	DFT λ (*nm*)	*E*(*eV*)	f_{os}	MO contributions
1	818.864	1.514	1.578	H→L (92%), H-1→L (6%)
2	637.385	1.945	0.871	H-2→L (12%), H-1→L (78%), H→L (7%)
3	551.580	2.248	0.249	H-1→L+1 (10%), H→L+1 (79%), H-2→L+1 (3%), H→L+2 (5%)
4	531.984	2.331	0.115	H-2→L (82%), H-1→L (13%), H-3→L (4%)
5	499.775	2.481	0.700	H→L+2 (85%), H-1→L+1 (3%), H→L+1 (3%), H→L+3 (3%)
6	472.789	2.622	0.043	H-2→L+1 (19%), H-1→L+1 (59%), H→L+1 (17%)

MO=molecular orbital, H=HOMO, L=LUMO, f_{os}= oscillator strength, wavelength= λ (*nm*)

Table S12: Wavelength, excitation energy and oscillator strength of **ATTD3** in chloroform solvent at M06/6-311G (d,p) level.

NO	DFT λ (*nm*)	*E*(*eV*)	f_{os}	MO contributions
1	846.944	1.464	1.399	H→L (92%), H-1→L (5%)
2	781.052	1.587	0.035	H→L+1 (88%), H-2→L+1 (3%), H-1→L+1 (8%)
3	655.446	1.892	0.797	H-2→L (12%), H-1→L (65%), H-2→L+1 (3%), H-1→L+1 (7%), H→L (6%), H→L+1 (2%)
4	636.306	1.949	0.350	H-2→L+1 (16%), H-1→L+1 (49%), H→L+1 (10%), H-1→L (8%), H-1→L+2 (2%), H→L+2 (9%)
5	600.030	2.066	0.044	H→L+2 (84%), H-1→L (5%), H-1→L+1 (7%)
6	542.719	2.285	0.190	H-2→L (77%), H-1→L (14%), H-3→L (4%)

MO=molecular orbital, H=HOMO, L=LUMO, f_{os}= oscillator strength, wavelength= λ (*nm*)

Table S13: Wavelength, excitation energy and oscillator strength of **ATTD4** in chloroform solvent at M06/6-311G (d,p) level.

NO	DFT λ (*nm*)	*E*(*eV*)	f_{os}	MO contributions

1	799.434	1.551	1.465	H→L (88%), H-1→L (8%)
2	690.835	1.795	0.324	H-1→L+1 (13%), H→L+1 (78%), H-2→L+1 (4%)
3	632.766	1.959	0.635	H-2→L (14%), H-1→L (68%), H→L (11%), H→L+1 (3%)
4	570.514	2.173	0.296	H-2→L+1 (16%), H-1→L+1 (59%), H→L+1 (17%), H-1→L (3%)
5	520.133	2.384	0.125	H-2→L (76%), H-1→L (16%), H-3→L (4%)
6	503.612	2.462	0.832	H→L+2 (90%), H→L+3 (3%)

MO=molecular orbital, H=HOMO, L=LUMO, f_{os}= oscillator strength, wavelength= λ (*nm*)

Table S14: Wavelength, excitation energy and oscillator strength of **ATTD5** in chloroform solvent at M06/6-311G (d,p) level.

NO	DFT λ (*nm*)	*E*(*eV*)	f_{os}	MO contributions
1	816.169	1.519	1.474	H→L (90%), H-1→L (6%)
2	713.825	1.737	0.262	H-1→L+1 (11%), H→L+1 (82%), H-2→L+1 (4%)
3	638.337	1.942	0.640	H-2→L (15%), H-1→L (70%), H→L (9%), H→L+1 (2%)
4	584.170	2.122	0.291	H-2→L+1 (18%), H-1→L+1 (60%), H→L+1 (14%), H-1→L (4%)
5	528.627	2.345	0.159	H-2→L (75%), H-1→L (17%), H-3→L (4%)
6	506.079	2.450	0.812	H→L+2 (87%), H→L+3 (6%)

MO=molecular orbital, H=HOMO, L=LUMO, f_{os}= oscillator strength, wavelength= λ (*nm*)

Table S15: Wavelength, excitation energy and oscillator strength of **ATTD6** in chloroform solvent at M06/6-311G (d,p) level.

NO	DFT λ (*nm*)	E(*eV*)	f_{os}	MO contributions
1	835.698	1.484	1.368	H→L (90%), H-1→L (6%)
2	749.104	1.655	0.216	H→L+1 (85%), H-2→L+1 (3%), H-1→L+1 (9%)
3	651.725	1.902	0.658	H-2→L (16%), H-1→L (69%), H→L (8%)
4	609.349	2.035	0.387	H-2→L+1 (19%), H-1→L+1 (61%), H→L+1 (12%), H-1→L (4%)
5	539.296	2.299	0.181	H-2→L (75%), H-1→L (18%), H-3→L (4%)
6	509.887	2.432	0.819	H→L+2 (53%), H→L+3 (34%), H-1→L+1 (4%), H→L+4 (2%)

MO=molecular orbital, H=HOMO, L=LUMO, f_{os}= oscillator strength, wavelength= λ (*nm*)

Table S16: Wavelength, excitation energy and oscillator strength of **ATTD7** in chloroform solvent at M06/6-311G (d,p) level.

NO	DFT λ (*nm*)	*E*(*eV*)	f_{os}	MO contributions
1	836.093	1.483	1.431	H→L (90%), H-1→L (6%)
2	754.850	1.643	0.201	H→L+1 (85%), H-2→L+1 (3%), H-1→L+1 (9%)
3	649.302	1.910	0.622	H-2→L (15%), H-1→L (68%), H→L (8%)
4	610.910	2.030	0.416	H-2→L+1 (18%), H-1→L+1 (61%), H→L+1 (11%), H-1→L (6%)

5	535.916	2.314	0.169	H-2→L (75%), H-1→L (18%), H-3→L (4%)
6	512.458	2.419	0.755	H→L+2 (46%), H→L+3 (42%), H-1→L+1 (3%), H→L+4 (3%)

MO=molecular orbital, H=HOMO, L=LUMO, f_{os}= oscillator strength, wavelength= λ (*nm*)

Table S17: Wavelength, excitation energy and oscillator strength of **ATTD8** in chloroform solvent at M06/6-311G (d,p) level.

NO	DFT λ (*nm*)	*E*(*eV*)	f_{os}	MO contributions
1	796.558	1.557	1.439	H→L (88%), H-1→L (8%)
2	688.419	1.801	0.308	H-1→L+1 (13%), H→L+1 (79%), H-2→L+1 (4%)
3	633.445	1.957	0.657	H-2→L (15%), H-1→L (68%), H→L (11%), H→L+1 (3%)
4	571.277	2.170	0.286	H-2→L+1 (17%), H-1→L+1 (58%), H→L+1 (17%), H-1→L (3%)
5	523.339	2.369	0.147	H-2→L (75%), H-1→L (17%), H-3→L (4%)
6	503.407	2.463	0.842	H→L+2 (87%), H→L+3 (4%)

MO=molecular orbital, H=HOMO, L=LUMO, f_{os}= oscillator strength, wavelength= λ (*nm*)

Table S18: Wave length λ (*nm*), excitation energy (*E*) and oscillator strength (*f*) of **ATTR1** in gaseous phase at M06/6-311G (d,p) level.

NO	DFT λ (*nm*)	*E*(*eV*)	f_{os}	MO contributions
1	695.057	1.784	3.007	H→L (95%), H-1→L+1 (4%)
2	579.041	2.141	0.001	H→L+1 (97%)
3	540.095	2.296	0.003	H-1→L (95%), H-2→L+1 (3%)
4	489.456	2.533	0.215	H-1→L+1 (92%), H→L (4%)
5	479.277	2.587	0.080	H-1→L+3 (14%), H→L+2 (81%)
6	470.368	2.636	0.001	H-1→L+2 (16%), H→L+3 (78%)

MO=molecular orbital, H=HOMO, L=LUMO, f_{os}= oscillator strength, wavelength= λ (*nm*)

Table S19: Wave length λ (*nm*), excitation energy (*E*) and oscillator strength (*f*) of **ATTD2** in gaseous phase at M06/6-311G (d,p) level.

NO	DFT λ (*nm*)	*E*(*eV*)	f_{os}	MO contributions
1	767.181	1.616	1.310	H→L (96%), H-1→L (2%)
2	600.204	2.066	1.118	H-1→L (85%), H-2→L (6%), H→L (3%), H→L+2 (3%)
3	526.293	2.356	0.265	H→L+1 (76%), H-2→L+1 (2%), H-1→L (3%), H-1→L+1 (9%), H→L+2 (5%)
4	511.359	2.425	0.109	H-2→L (83%), H-3→L (2%), H-1→L (7%), H→L+1 (4%)
5	481.548	2.575	0.501	H→L+2 (82%), H-2→L (5%), H→L+1 (3%), H→L+3 (2%)
6	454.338	2.729	0.027	H-2→L+1 (16%), H-1→L+1 (63%), H→L+1 (15%)

MO=molecular orbital, H=HOMO, L=LUMO, f_{os}= oscillator strength, wavelength= λ (*nm*)

Table S20: Wavelength, excitation energy and oscillator strength of **ATTD3** in gaseous phase at M06/6-311G (d,p) level.

NO	DFT λ (*nm*)	*E*(*eV*)	f_{os}	MO contributions
1	832.444	1.489	1.035	H→L (97%)
2	772.295	1.605	0.045	H→L+1 (92%), H-2→L+1 (2%), H-1→L+1 (5%)
3	634.417	1.954	0.627	H-1→L (57%), H-1→L+1 (17%), H-2→L (8%), H-2→L+1 (9%), H→L (2%), H→L+1 (3%)
4	616.960	2.010	0.679	H-2→L+1 (14%), H-1→L (27%), H-1→L+1 (46%), H→L+1 (5%), H→L+2 (2%)
5	555.410	2.232	0.164	H→L+2 (86%), H-2→L (3%), H-1→L (5%)
6	534.968	2.318	0.308	H-2→L (75%), H-1→L (8%), H-1→L+1 (4%), H→L+2 (6%)

MO=molecular orbital, H=HOMO, L=LUMO, f_{os}= oscillator strength, wavelength= λ (*nm*)

Table S21: Wavelength, excitation energy and oscillator strength of **ATTD4** in gaseous phase at M06/6-311G (d,p) level.

NO	DFT λ (*nm*)	*E*(*eV*)	f_{os}	MO contributions
1	765.098	1.621	1.215	H→L (94%), H-1→L (3%)
2	670.402	1.849	0.216	H-1→L+1 (11%), H→L+1 (81%), H-2→L+1 (3%)
3	603.711	2.054	0.827	H-2→L (10%), H-1→L (72%), H→L (5%), H→L+1 (5%), H→L+2 (3%)
4	555.087	2.234	0.436	H-2→L+1 (15%), H-1→L+1 (58%), H→L+1 (13%), H-1→L (8%), H→L+2 (2%)
5	507.010	2.445	0.317	H-2→L (73%), H-1→L (13%), H-3→L (2%), H→L+2 (9%)
6	484.749	2.558	0.488	H-2→L (10%), H→L+2 (79%), H-1→L+1 (4%), H→L+3 (3%)

MO=molecular orbital, H=HOMO, L=LUMO, f_{os}= oscillator strength, wavelength= λ (*nm*)

Table S22: Wavelength, excitation energy and oscillator strength of **ATTD5** in gaseous phase at M06/6-311G (d,p) level.

NO	DFT λ (*nm*)	*E*(*eV*)	f_{os}	MO contributions
1	790.212	1.569	1.169	H→L (96%), H-1→L (2%)
2	702.102	1.766	0.170	H→L+1 (86%), H-2→L+1 (3%), H-1→L+1 (8%)
3	613.297	2.022	0.801	H-2→L (11%), H-1→L (72%), H-2→L+1 (3%), H-1→L+1 (3%), H→L (4%), H→L+1 (3%), H→L+2 (2%)
4	573.178	2.163	0.432	H-2→L+1 (17%), H-1→L (10%), H-1→L+1 (57%), H→L+1 (10%)
5	519.654	2.386	0.381	H-2→L (73%), H-1→L (13%), H→L+2 (8%)
6	488.608	2.538	0.487	H→L+2 (72%), H-2→L (8%), H-2→L+1 (3%), H-1→L+1 (8%), H→L+3 (4%)

MO=molecular orbital, H=HOMO, L=LUMO, f_{os}= oscillator strength, wavelength= λ (*nm*)

Table S23: Wavelength, excitation energy and oscillator strength of **ATTD6** in gaseous phase at M06/6-311G (d,p) level.

**NO**	**DFT** λ (*nm*)	$E(eV)$	f_{os}	**MO contributions**
1	822.122	1.508	1.041	H→L (97%), H-1→L (2%)
2	746.713	1.660	0.121	H→L+1 (90%), H-2→L+1 (2%), H-1→L+1 (6%)
3	630.161	1.968	0.735	H-2→L (10%), H-1→L (68%), H-2→L+1 (5%), H-1→L+1 (6%), H→L (3%), H→L+1 (3%)
4	600.902	2.063	0.552	H-2→L+1 (17%), H-1→L (15%), H-1→L+1 (56%), H→L+1 (7%)
5	533.908	2.322	0.462	H-2→L (74%), H-1→L (13%), H→L+2 (5%), H→L+3 (2%)
6	505.418	2.453	0.169	H-2→L+1 (55%), H-1→L+1 (29%), H-3→L+1 (3%), H→L+2 (8%), H→L+3 (3%)

MO=molecular orbital, H=HOMO, L=LUMO, f_{os}= oscillator strength, wavelength= λ (*nm*)

Table S24: Wavelength, excitation energy and oscillator strength of **ATTD7** in gaseous phase at M06/6-311G (d,p) level.

**NO**	**DFT** λ (*nm*)	$E(eV)$	f_{os}	**MO contributions**
1	822.940	1.507	1.073	H→L (97%)
2	753.245	1.646	0.134	H→L+1 (90%), H-2→L+1 (2%), H-1→L+1 (6%)
3	629.553	1.969	0.629	H-2→L (10%), H-1→L (63%), H-2→L+1 (7%), H-1→L+1 (9%), H→L (3%), H→L+1 (3%)
4	601.252	2.062	0.676	H-2→L+1 (15%), H-1→L (20%), H-1→L+1 (54%), H→L+1 (6%)
5	531.573	2.332	0.424	H-2→L (74%), H-1→L (13%), H-3→L (2%), H→L+2 (5%), H→L+3 (3%)
6	504.760	2.456	0.184	H-2→L+1 (52%), H-1→L+1 (28%), H→L+2 (10%), H-3→L+1 (3%), H→L+3 (4%)

MO=molecular orbital, H=HOMO, L=LUMO, f_{os}= oscillator strength, wavelength= λ (*nm*)

Table S25: Wavelength, excitation energy and oscillator strength of **ATTD8** in gaseous phase at M06/6-311G (d,p) level.

**NO**	**DFT** λ (*nm*)	$E(eV)$	f_{os}	**MO contributions**
1	757.850	1.636	1.234	H→L (94%), H-1→L (4%)
2	659.420	1.880	0.191	H-1→L+1 (12%), H→L+1 (80%), H-2→L+1 (4%)
3	601.427	2.062	0.892	H-2→L (10%), H-1→L (72%), H→L (5%), H→L+1 (5%), H→L+2 (3%)
4	550.747	2.251	0.360	H-2→L+1 (16%), H-1→L+1 (56%), H→L+1 (15%), H-1→L (7%)
5	507.861	2.441	0.347	H-2→L (72%), H-1→L (13%), H-3→L (2%), H→L+2 (9%)
6	484.389	2.560	0.497	H-2→L (10%), H→L+2 (78%), H-1→L+1 (4%), H→L+3 (3%)

Table S26: Natural bond orbitals analysis for the reference compound (**ATTR1**) with its representative values

**Donor(*i*)**	**Type**	**Acceptor(*j*)**	**Type**	***E*(2) [*kcal/mol*]**	***E*(*j*)-*E*(*i*) [*a.u*]**	***F*(*i,j*) [*a.u*]**
C23-C31	π	C33-C54	π*	33.74	0.3	0.091
C33-C54	π	C56-C57	π*	29.03	0.29	0.083
C34-C35	π	C47-O52	π*	24.88	0.3	0.08
C43-C44	π	C41-C42	π*	21.8	0.3	0.073
C9-C10	π	C11-C20	π*	20.07	0.3	0.071
C60-C61	π	C55-O58	π*	19.2	0.3	0.07
C11-C20	π	C9-C10	π*	18.5	0.32	0.071
C23-C31	π	C24-C25	π*	15.05	0.27	0.059
C24-C25	π	C11-C20	π*	13.26	0.31	0.059
C33-C54	π	C23-C31	π*	11.96	0.29	0.055
C27-C28	π	C90-C92	π*	11.64	0.31	0.055
C56-C57	π	C59-C64	π*	8.83	0.33	0.05
C37-C38	π	C34-C35	π*	8.06	0.33	0.047
C55-O58	π	C60-C61	π*	4.05	0.44	0.041
C47-O52	π	C34-C35	π*	3.59	0.42	0.038
C48-N49	π	C50-N51	π*	0.83	0.47	0.018
C67-N68	π	C69-N70	π*	0.78	0.48	0.017
C33-H53	σ	S26-C31	σ*	10.71	0.72	0.078
C25-S26	σ	C24-S101	σ*	8.72	0.86	0.077
C69-N70	σ	C57-C69	σ*	8.08	1.57	0.101
C67-N68	σ	C57-C67	σ*	7.97	1.58	0.101
C97-H100	σ	C89-S101	σ*	7.49	0.71	0.065
C11-C20	σ	C10-C21	σ*	6.76	1.18	0.08
C23-C24	σ	C24-C25	σ*	5.96	1.27	0.078
C40-C41	σ	C37-C42	σ*	5.77	1.19	0.074
C2-C3	σ	C3-C4	σ*	5.18	1.25	0.072
C10-C11	σ	C20-C25	σ*	4.94	1.24	0.07
C1-H7	σ	C5-C6	σ*	4.67	1.08	0.064
C93-H94	σ	C90-C92	σ*	4.3	0.54	0.046
C63-H105	σ	C61-C62	σ*	4.09	1.1	0.06
C64-H66	σ	C62-C63	σ*	3.92	1.11	0.059
C28-C29	σ	C27-C34	σ*	3.66	1.27	0.061
C11-H12	σ	C9-C10	σ*	3.08	1.11	0.052
C56-C57	σ	C59-C60	σ*	1.03	1.33	0.033
C89-C97	σ	C97-H100	σ*	0.57	1.08	0.022
S17-C20	σ	C11-C20	σ*	0.53	1.22	0.023
C67-N68	σ	C56-C57	σ*	0.5	1.65	0.026
S26	LP(2)	C24-C25	π*	28.61	0.25	0.078
S18	LP(2)	C14-C15	π*	22.8	0.27	0.071
S30	LP(2)	C27-C28	π*	19.08	0.27	0.066
O52	LP(2)	C43-C47	σ*	20.79	0.76	0.114
O52	LP(2)	C35-C47	σ*	18.06	0.76	0.106
S30	LP(1)	C27-C34	σ*	0.52	1.23	0.023

**Donor(*i*)**	**Type**	**Acceptor(*j*)**	**Type**	***E*(2) [*kcal/mol*]**	***E*(*j*)-*E*(*i*) [*a.u*]**	***F*(*i,j*) [*a.u*]**
C27-C28	π	C33-C34	π*	34.84	0.3	0.092
C33-C34	π	C36-C37	π*	29.88	0.29	0.084
C89-C93	π	C86-C87	π*	25.67	0.3	0.079
C39-C40	π	C41-C42	π*	23.5	0.3	0.075
C107-C108	π	C109-C112	π*	22.55	0.31	0.074
C86-C87	π	C89-C93	π*	20.98	0.29	0.07
C11-C20	π	C9-C10	π*	19.65	0.32	0.074
C5-C6	π	C1-C2	π*	18.84	0.3	0.069
C23-C31	π	C24-C25	π*	17.02	0.29	0.067
C29-C32	π	C14-C15	π*	13.21	0.31	0.059
C23-C31	π	C86-C87	π*	10.52	0.32	0.054
C36-C37	π	C41-C42	π*	8.12	0.33	0.048
C46-O51	π	C41-C42	π*	4.03	0.43	0.041
C46-O51	π	C33-C34	π*	3.56	0.42	0.038
C47-N48	π	C49-N50	π*	0.84	0.47	0.018
C49-N50	π	C47-N48	π*	0.79	0.48	0.017
C107-C108	π	C97-C99	π*	0.76	0.3	0.014
C33-H35	σ	C27-S30	σ*	10.85	0.72	0.079
C25-S26	σ	C24-S82	σ*	9.2	0.86	0.079
S30-C32	σ	C29-S83	σ*	8.7	0.86	0.077
C2-C3	σ	S18-C19	σ*	7.85	0.89	0.075
C68-H69	σ	C70-S82	σ*	6.9	0.72	0.063
C23-C24	σ	C20-C25	σ*	6.11	1.21	0.077
C14-C15	σ	C15-C32	σ*	5.84	1.27	0.077
S30-C32	σ	C15-S18	σ*	5.74	0.85	0.062
C68-C70	σ	C23-C31	σ*	5.19	1.35	0.075
C70-S82	σ	C24-C25	σ*	4.98	1.24	0.07
C99-H103	σ	C97-C98	σ*	4.84	1.1	0.065
C109-H113	σ	C107-C108	σ*	4.64	1.11	0.064
C97-C98	σ	C98-C100	σ*	3.96	1.31	0.065
C38-H85	σ	C42-C43	σ*	3.79	1.13	0.058
C24-S82	σ	C70-C78	σ*	2.98	1.12	0.052
C24-C25	σ	C11-C20	σ*	2.37	1.33	0.05
C34-C46	σ	C46-O51	σ*	1.99	1.25	0.045
C5-C21	σ	C9-S17	σ*	0.61	0.83	0.02
C123-H126	σ	C104-O118	σ*	0.54	0.91	0.02
C73-S83	σ	C28-C29	σ*	0.51	1.2	0.022
O118	LP(2)	C102-C104	π*	32.47	0.36	0.103
S83	LP(2)	C71-C73	π*	24.45	0.29	0.076
S30	LP(2)	C33-C34	π*	0.52	0.28	0.011
O51	LP(2)	C42-C46	σ*	20.86	0.76	0.114
N50	LP(1)	C37-C49	σ*	12.49	1.05	0.102
S83	LP(1)	S18-C19	σ*	0.52	0.89	0.019

Table S27: Natural bond orbitals analysis for **ATTD2** with its representative values

Table S28: Natural bond orbitals analysis for **ATTD3** with its representative values

Donor(*i*)	Type	Acceptor(*j*)	Type	*E*(2) [*kcal/mol*]	*E*(*j*)-*E*(*i*) [*a.u*]	*F*(*i,j*) [*a.u*]
C27-C28	π	C33-C34	π*	36.23	0.3	0.093
C33-C34	π	C36-C37	π*	28.21	0.29	0.081
C83-C87	π	C80-C81	π*	25.61	0.3	0.079
C80-C81	π	C82-C85	π*	22.59	0.3	0.074
C123-C125	π	C121-C126	π*	20.37	0.31	0.072
C92-C94	π	C91-C93	π*	19.33	0.3	0.07
C40-C41	π	C36-C37	π*	18.95	0.32	0.071
C14-C15	π	C13-C19	π*	17.99	0.32	0.07
C11-C20	π	C24-C25	π*	15.62	0.29	0.065
C29-C32	π	C14-C15	π*	12.96	0.31	0.058
C23-C31	π	C80-C81	π*	10.72	0.32	0.054
C36-C37	π	C33-C34	π*	7.76	0.33	0.047
N131-O132	π	C121-C126	π*	2.92	0.52	0.038
C36-C37	π	C43-N44	π*	1.16	0.41	0.02
C43-N44	π	C45-N46	π*	0.85	0.47	0.018
C45-N46	π	C43-N44	π*	0.79	0.47	0.017
C101-C102	π	C91-C93	π*	0.76	0.3	0.014
C33-H35	σ	C27-S30	σ*	10.77	0.72	0.079
C25-S26	σ	C24-S78	σ*	9.19	0.86	0.079
S30-C32	σ	C29-S79	σ*	8.64	0.86	0.077
C2-C3	σ	S18-C19	σ*	7.86	0.89	0.075
C15-C32	σ	C29-C32	σ*	6.87	1.31	0.085
C23-C24	σ	C20-C25	σ*	6.08	1.21	0.077
C34-C42	σ	C41-S127	σ*	5.88	0.89	0.065
C28-C29	σ	C15-C32	σ*	5.62	1.25	0.075
C13-C19	σ	C3-C4	σ*	5.05	1.29	0.072
C66-S78	σ	C24-C25	σ*	4.97	1.24	0.07
C66-S78	σ	C64-H65	σ*	4.53	1.06	0.062
C92-H95	σ	C94-C98	σ*	4.07	1.09	0.06
C38-C121	σ	C126-N131	σ*	3.98	1.03	0.058
C29-S79	σ	C69-C70	σ*	3.18	1.13	0.054
C67-H68	σ	C28-C29	σ*	2.86	1.07	0.049
C104-C108	σ	C102-H105	σ*	2.28	1.14	0.046
C21-C60	σ	C5-C21	σ*	1.99	1.05	0.041
C22-C48	σ	C48-H50	σ*	0.6	1.01	0.022
C117-H120	σ	C98-O112	σ*	0.53	0.91	0.02
C69-S79	σ	C28-C29	σ*	0.51	1.2	0.022
O112	LP(2)	C96-C98	π*	32.44	0.36	0.103
S26	LP(2)	C24-C25	π*	22.66	0.27	0.071
S30	LP(2)	C33-C34	π*	0.54	0.28	0.011
O47	LP(2)	C41-C42	σ*	22.65	0.74	0.117
N46	LP(1)	C37-C45	σ*	12.59	1.04	0.102
O129	LP(2)	C125-C126	σ*	0.5	0.84	0.019

Table S29: Natural bond orbitals analysis for **ATTD4** with its representative values.

**Donor(*i*)**	**Type**	**Acceptor(*j*)**	**Type**	***E*(2) [*kcal/mol*]**	***E*(*j*)-*E*(*i*) [*a.u*]**	***F*(*i,j*) [*a.u*]**
C27-C28	π	C33-C34	π*	34.1	0.3	0.091
C83-C87	π	C80-C81	π*	25.81	0.3	0.079
C3-C4	π	C5-C6	π*	24.76	0.3	0.077
C80-C81	π	C82-C85	π*	22.7	0.3	0.074
C40-C41	π	C42-O47	π*	21.28	0.33	0.075
C39-C123	π	C40-C41	π*	19.5	0.28	0.067
C104-C108	π	C103-C106	π*	18.41	0.31	0.068
C82-C85	π	C80-C81	π*	18.07	0.3	0.068
C5-C6	π	C9-C10	π*	16.18	0.29	0.062
C9-C10	π	C5-C6	π*	15.75	0.32	0.066
C24-C25	π	C11-C20	π*	14.57	0.32	0.064
C27-C28	π	C67-C69	π*	11.57	0.31	0.055
C36-C37	π	C33-C34	π*	7.79	0.33	0.047
C42-O47	π	C40-C41	π*	4.24	0.41	0.041
C36-C37	π	C43-N44	π*	1.17	0.41	0.021
C43-N44	π	C45-N46	π*	0.84	0.47	0.018
C45-N46	π	C43-N44	π*	0.77	0.48	0.017
C33-H35	σ	C27-S30	σ*	10.58	0.72	0.078
C25-S26	σ	C24-S78	σ*	9.21	0.86	0.079
C37-C45	σ	C45-N46	σ*	8.16	1.61	0.103
C20-C25	σ	C24-C25	σ*	7.76	1.33	0.091
C27-C33	σ	C33-C34	σ*	6.89	1.34	0.086
C23-C24	σ	C20-C25	σ*	6.08	1.21	0.077
C28-C29	σ	C15-C32	σ*	5.68	1.24	0.075
C64-C66	σ	C23-C31	σ*	5.19	1.35	0.075
C66-S78	σ	C24-C25	σ*	4.97	1.24	0.07
C82-H86	σ	C80-C81	σ*	4.35	1.09	0.062
C96-H100	σ	C94-C98	σ*	4.04	1.1	0.06
S18-C19	σ	C13-C22	σ*	3.69	1.15	0.059
C14-H16	σ	C13-C19	σ*	3.09	1.11	0.052
C125-C126	σ	C123-H124	σ*	2.02	1.17	0.044
C10-C21	σ	C60-H63	σ*	1.49	1.01	0.035
C33-C34	σ	C41-C42	σ*	1.06	1.19	0.032
C23-C24	σ	C25-S26	σ*	0.56	0.88	0.02
C117-H120	σ	C98-O112	σ*	0.54	0.91	0.02
S17-C20	σ	C10-C21	σ*	0.51	1.11	0.021
O111	LP(2)	C104-C108	π*	32.59	0.36	0.103
S78	LP(2)	C64-C66	π*	22.01	0.29	0.072
S30	LP(2)	C33-C34	π*	0.5	0.28	0.011
O47	LP(2)	C41-C42	σ*	22.21	0.74	0.116
O112	LP(2)	C117-H119	σ*	6.08	0.69	0.06
Cl128	LP(2)	C38-C121	σ*	0.5	0.9	0.019

Table S30: Natural bond orbitals analysis for **ATTD5** with its representative values

**Donor(*i*)**	**Type**	**Acceptor(*j*)**	**Type**	***E*(2) [*kcal/mol*]**	***E*(*j*)-*E*(*i*) [*a.u*]**	***F*(*i,j*) [*a.u*]**
C27-C28	π	C33-C34	π*	35.22	0.3	0.093
C33-C34	π	C36-C37	π*	27.35	0.29	0.08
C85-C87	π	C80-C82	π*	25.89	0.3	0.079
C96-C98	π	C91-C93	π*	24.45	0.3	0.078
C29-C32	π	C27-C28	π*	23.12	0.31	0.08
C80-C82	π	C81-C83	π*	22.85	0.3	0.074
C92-C94	π	C96-C98	π*	22.72	0.29	0.075
C101-C102	π	C103-C106	π*	22.61	0.31	0.074
C103-C106	π	C104-C108	π*	22.29	0.3	0.075
C81-C83	π	C85-C87	π*	21.72	0.3	0.074
C38-C39	π	C123-C125	π*	19.41	0.31	0.071
C64-C66	π	C23-C31	π*	18.23	0.32	0.074
C23-C31	π	C24-C25	π*	17.07	0.29	0.067
C36-C37	π	C40-C41	π*	9.09	0.31	0.049
C42-O47	π	C40-C41	π*	4.28	0.41	0.041
C101-C102	π	C91-C93	π*	0.84	0.3	0.014
C45-N46	π	C43-N44	π*	0.78	0.48	0.017
C33-H35	σ	C27-S30	σ*	10.72	0.72	0.078
C37-C43	σ	C43-N44	σ*	8.41	1.61	0.104
C20-C25	σ	C24-C25	σ*	7.76	1.33	0.091
C29-C32	σ	C15-C32	σ*	6.83	1.3	0.084
C123-C125	σ	C125-C126	σ*	6.19	1.27	0.079
C3-C4	σ	C2-C3	σ*	5.77	1.27	0.076
C2-C22	σ	C13-C14	σ*	5.53	1.19	0.073
C1-C6	σ	C5-C6	σ*	5.51	1.27	0.075
C4-H8	σ	C5-C6	σ*	5.15	1.08	0.067
C5-C6	σ	C1-C6	σ*	5.04	1.25	0.071
C93-H97	σ	C91-C92	σ*	4.84	1.1	0.065
C91-C92	σ	C91-C93	σ*	4.76	1.3	0.07
C93-C96	σ	N90-C91	σ*	4.63	1.13	0.065
C94-C98	σ	C96-C98	σ*	4.8	1.28	0.07
C103-C106	σ	N90-C101	σ*	4.5	1.14	0.064
O111-C113	σ	C106-C108	σ*	3.08	1.42	0.059
N90-C91	σ	C87-N90	σ*	2.58	1.21	0.05
C21-C60	σ	C60-H63	σ*	0.61	1.01	0.022
C117-H120	σ	C98-O112	σ*	0.54	0.91	0.02
C45-N46	σ	C36-C37	σ*	0.5	1.64	0.026
O112	LP(2)	C96-C98	π*	32.51	0.36	0.103
S17	LP(2)	C11-C20	π*	23.61	0.28	0.073
F134	LP(2)	C123-C125	π*	0.52	0.48	0.015
O47	LP(2)	C41-C42	σ*	22.37	0.74	0.117
O47	LP(1)	S30-C32	σ*	1.21	0.93	0.03
F130	LP(2)	C125-C126	σ*	0.51	1	0.02

Table S31: Natural bond orbitals analysis for **ATTD6** with its representative values.

**Donor(*i*)**	**Type**	**Acceptor(*j*)**	**Type**	***E*(2) [*kcal/mol*]**	***E*(*j*)-*E*(*i*) [*a.u*]**	***F*(*i,j*) [*a.u*]**
C27-C28	π	C33-C34	π*	36.16	0.3	0.093
C33-C34	π	C36-C37	π*	28.25	0.29	0.081
C33-C34	π	C42-O47	π*	26.76	0.3	0.081
C104-C108	π	C101-C102	π*	24.11	0.3	0.077
C91-C93	π	C92-C94	π*	22.59	0.31	0.074
C81-C83	π	C85-C87	π*	21.69	0.3	0.074
C40-C41	π	C42-O47	π*	19.97	0.33	0.073
C85-C87	π	C81-C83	π*	18.89	0.3	0.068
C24-C25	π	C11-C20	π*	14.33	0.32	0.063
C29-C32	π	C14-C15	π*	12.97	0.31	0.058
C23-C31	π	C80-C82	π*	10.12	0.32	0.053
C36-C37	π	C40-C41	π*	9.14	0.31	0.049
C36-C37	π	C33-C34	π*	7.75	0.33	0.047
C42-O47	π	C40-C41	π*	4.3	0.41	0.041
C36-C37	π	C43-N44	π*	1.19	0.41	0.021
C43-N44	π	C45-N46	π*	0.82	0.47	0.018
C101-C102	π	C91-C93	π*	0.77	0.3	0.014
C33-H35	σ	C27-S30	σ*	10.74	0.72	0.079
C25-S26	σ	C24-S78	σ*	9.2	0.86	0.079
S30-C32	σ	C29-S79	σ*	8.63	0.86	0.077
C24-C25	σ	C20-C25	σ*	7.68	1.29	0.089
C27-C33	σ	C33-C34	σ*	6.85	1.34	0.085
C23-C24	σ	C20-C25	σ*	6.07	1.21	0.077
C34-C42	σ	C41-S127	σ*	5.91	0.88	0.065
C67-C69	σ	C27-C28	σ*	5.71	1.29	0.077
C121-H122	σ	C38-C39	σ*	4.99	1.06	0.065
C102-H105	σ	C101-C103	σ*	4.81	1.1	0.065
C28-C67	σ	C67-C69	σ*	4.22	1.3	0.066
C36-C40	σ	C33-C34	σ*	3.97	1.28	0.064
C52-H53	σ	C13-C22	σ*	3.73	0.95	0.053
C24-S78	σ	C66-C74	σ*	2.98	1.12	0.052
C96-C98	σ	C94-H99	σ*	1.99	1.14	0.043
N90-C91	σ	C93-C96	σ*	1.49	1.36	0.04
S18-C19	σ	C13-C19	σ*	1.02	1.28	0.032
C23-C24	σ	C25-S26	σ*	0.56	0.88	0.02
S17-C20	σ	C10-C21	σ*	0.51	1.11	0.021
C45-N46	σ	C36-C37	σ*	0.5	1.64	0.026
O112	LP(2)	C96-C98	π*	32.44	0.36	0.103
S79	LP(2)	C67-C69	π*	24.62	0.29	0.076
S30	LP(2)	C33-C34	π*	0.53	0.28	0.011
O131	LP(1)	S128-O129	σ*	2.68	1.02	0.048
O129	LP(1)	S128-O130	σ*	1.61	1.17	0.04
O136	LP(2)	C123-C125	σ*	0.5	0.97	0.02

Table S32: Natural bond orbitals analysis for **ATTD7** with its representative values.

**Donor(*i*)**	**Type**	**Acceptor(*j*)**	**Type**	***E*(2) [*kcal/mol*]**	***E*(*j*)-*E*(*i*) [*a.u*]**	***F*(*i,j*) [*a.u*]**
C27-C28	π	C33-C34	π*	35.93	0.3	0.093
C33-C34	π	C36-C37	π*	27.92	0.29	0.081
C33-C34	π	C42-O47	π*	26.79	0.3	0.081
C39-C123	π	C125-C126	π*	24.52	0.27	0.075
C29-C32	π	C27-C28	π*	23.38	0.31	0.081
C92-C94	π	C96-C98	π*	22.6	0.29	0.075
C3-C4	π	C1-C2	π*	21.08	0.3	0.073
C92-C94	π	C91-C93	π*	19.33	0.3	0.07
C23-C31	π	C80-C81	π*	10.39	0.32	0.054
C36-C37	π	C40-C41	π*	9.03	0.31	0.049
C36-C37	π	C33-C34	π*	7.77	0.33	0.047
C42-O47	π	C40-C41	π*	4.32	0.41	0.041
C42-O47	π	C33-C34	π*	3.47	0.43	0.038
C36-C37	π	C43-N44	π*	1.09	0.41	0.02
C67-C69	π	C67-C69	π*	0.91	0.32	0.016
C43-N44	π	C45-N46	π*	0.82	0.47	0.018
C45-N46	π	C43-N44	π*	0.76	0.47	0.017
C33-H35	σ	C27-S30	σ*	10.72	0.72	0.078
C25-S26	σ	C24-S78	σ*	9.2	0.86	0.079
C126-C130	σ	C130-N131	σ*	8.93	1.62	0.108
C2-C3	σ	S18-C19	σ*	7.87	0.89	0.075
C15-C32	σ	C29-C32	σ*	6.9	1.32	0.085
C38-C39	σ	C36-C40	σ*	6.39	1.18	0.078
C36-C37	σ	C37-C45	σ*	6.01	1.27	0.078
S30-C32	σ	C15-S18	σ*	5.72	0.85	0.062
C9-C10	σ	C1-C6	σ*	5.08	1.29	0.072
C66-S78	σ	C24-C25	σ*	4.97	1.24	0.07
S17-C20	σ	C11-H12	σ*	3.89	1.06	0.058
C85-C87	σ	N90-C91	σ*	3.56	1.13	0.057
C29-S79	σ	S30-C32	σ*	3.08	0.89	0.047
C125-C128	σ	C39-C123	σ*	2.99	1.3	0.056
C5-C21	σ	C3-C4	σ*	2.06	1.19	0.044
C10-C21	σ	C21-C60	σ*	1.73	1.01	0.038
C102-H105	σ	N90-C101	σ*	1.01	0.94	0.028
C66-S78	σ	C23-C24	σ*	0.58	1.17	0.023
C117-H120	σ	C98-O112	σ*	0.54	0.91	0.02
C69-S79	σ	C28-C29	σ*	0.51	1.2	0.022
N90	LP(1)	C83-C87	π*	32.73	0.29	0.091
S127	LP(2)	C38-C121	π*	23.85	0.27	0.072
S30	LP(2)	C33-C34	π*	0.53	0.28	0.011
O47	LP(2)	C41-C42	σ*	22.62	0.74	0.117
N90	LP(1)	C91-C93	σ*	3.88	0.85	0.055

S30	LP(1)	C27-C33	σ*	0.51	1.24	0.022

Table S33: Natural bond orbitals analysis for **ATTD8** with its representative values.

**Donor(*i*)**	**Type**	**Acceptor(*j*)**	**Type**	***E*(2) [*kcal/mol*]**	***E*(*j*)-*E*(*i*) [*a.u*]**	***F*(*i,j*) [*a.u*]**
C27-C28	π	C33-C34	π*	34.19	0.3	0.092
C33-C34	π	C36-C37	π*	26.56	0.29	0.079
C83-C87	π	C80-C81	π*	25.51	0.3	0.079
C125-C126	π	C38-C121	π*	24.92	0.28	0.074
C5-C6	π	C3-C4	π*	23.89	0.29	0.074
C1-C2	π	C5-C6	π*	22.77	0.3	0.076
C80-C81	π	C83-C87	π*	21.04	0.29	0.07
C13-C19	π	C14-C15	π*	20.6	0.29	0.072
C1-C2	π	C3-C4	π*	19.34	0.3	0.07
C96-C98	π	C92-C94	π*	18.63	0.31	0.068
C23-C31	π	C24-C25	π*	17.04	0.29	0.067
C40-C41	π	C39-C123	π*	16.6	0.33	0.069
C24-C25	π	C23-C31	π*	15.34	0.33	0.067
C36-C37	π	C33-C34	π*	7.82	0.33	0.047
C131-O132	π	C125-C126	π*	1.32	0.44	0.024
C36-C37	π	C43-N44	π*	0.98	0.41	0.019
C101-C102	π	C91-C93	π*	0.72	0.3	0.013
C33-H35	σ	C27-S30	σ*	10.61	0.72	0.078
C25-S26	σ	C24-S78	σ*	9.2	0.86	0.079
S30-C32	σ	C29-S79	σ*	8.73	0.86	0.077
C2-C3	σ	S18-C19	σ*	7.84	0.9	0.075
C15-C32	σ	C29-C32	σ*	6.91	1.32	0.085
C27-S30	σ	C28-C67	σ*	5.73	1.2	0.074
C64-C66	σ	C23-C31	σ*	5.19	1.35	0.075
C121-H122	σ	C38-C39	σ*	4.98	1.05	0.065
C93-H97	σ	C91-C92	σ*	4.81	1.1	0.065
C37-C43	σ	C34-C36	σ*	3.95	1.24	0.063
C9-C10	σ	C11-H12	σ*	3.69	1.11	0.057
C80-C82	σ	S26-C31	σ*	3.34	0.91	0.049
C38-C121	σ	C126-C131	σ*	2.98	1.18	0.054
C80-C82	σ	C85-H89	σ*	2.33	1.12	0.046
C96-C98	σ	C94-H99	σ*	2.01	1.14	0.043
C20-C25	σ	C23-C24	σ*	1.77	1.25	0.042
C36-C40	σ	C38-C39	σ*	1.01	1.2	0.031
C23-C24	σ	C25-S26	σ*	0.56	0.88	0.02
C117-H120	σ	C98-O112	σ*	0.54	0.91	0.02
C123-H124	σ	C125-C128	σ*	0.5	0.96	0.02
O130	LP(2)	C128-O129	π*	50.66	0.37	0.124
S17	LP(2)	C9-C10	π*	23.76	0.29	0.075
S30	LP(2)	C33-C34	π*	0.5	0.28	0.011
O132	LP(2)	C131-O133	σ*	33.13	0.68	0.135
O133	LP(2)	C138-H139	σ*	5.28	0.71	0.058

S79	LP(1)	S18-C19	σ*	0.51	0.89	0.019

Table S34: Dipole moment (*u*) and major contributing tensors (*D*) of the studied compounds.

**Compounds**	u_x	u_y	u_z	u_{total}

**ATTR1**	-0.0005	-0.0001	0.4618	0.46
**ATTD2**	-18.1919	1.1142	2.3406	18.38
**ATTD3**	27.2462	0.6546	0.7476	27.26
**ATTD4**	19.2814	-1.3926	0.2677	19.33
**ATTD5**	22.8245	-0.2264	0.2871	22.83
**ATTD6**	-24.6976	0.6917	1.9786	24.79
**ATTD7**	-28.0619	1.3336	-1.5738	28.14
**ATTD8**	-16.4007	1.2622	-1.2087	16.49

Table S35: Linear polarizabilities and major contributing tensors (*esu*) of the compounds (**ATTR1** and **ATTD2-ATTD8**).

**Compounds**	$\alpha_{xx} \times 10^{-22}$	$\alpha_{yy} \times 10^{-22}$	$\alpha_{zz} \times 10^{-22}$	$<\alpha> \times 10^{-22}$
**ATTR1**	6.30	2.19	0.65	3.05
**ATTD2**	5.84	2.21	0.87	2.97
**ATTD3**	6.24	2.35	0.99	3.19
**ATTD4**	5.98	2.37	0.91	3.09
**ATTD5**	6.05	2.36	0.92	3.11
**ATTD6**	6.23	2.39	1.02	3.21
**ATTD7**	6.30	2.39	0.92	3.20
**ATTD8**	5.98	2.41	0.99	3.13

Table S36: The calculated first hyperpolarizability (β_{tot}) along its major contributing tensors (*esu*) of **ATTR1** and **ATTD2-ATTD8**.

**Systems**	**ATTR1**	**ATTD2**	**ATTD3**	**ATTD4**	**ATTD5**	**ATTD6**	**ATTD7**	**ATTD8**
$\beta_{xxx}\times10^{-27}$	-0.0001	-6.97	8.06	5.98	6.70	-7.45	-7.74	-5.86
$\beta_{xxy}\times10^{-28}$	-0.0003	4.36	-7.50	-6.91	-8.97	-9.74	-7.41	-7.16
$\beta_{xyy}\times10^{-29}$	0.0003	-0.69	12.4	1.10	8.80	-12.0	-7.24	-3.83
$\beta_{yyy}\times10^{-30}$	0.001	-9.90	29.8	-16.4	-3.27	5.04	15.9	-8.46
$\beta_{xxz}\times10^{-29}$	5.00	-1.36	-17.0	-5.74	-15.8	14.5	6.51	13.6
$\beta_{yyz}\times10^{-30}$	3.60	-4.25	-11.3	-4.92	-1.47	9.83	5.61	3.97
$\beta_{xzz}\times10^{-30}$	0.001	4.31	6.97	-3.37	2.56	-4.96	2.92	-9.69
$\beta_{yzz}\times10^{-31}$	0.003	-1.84	8.08	3.41	-5.90	2.95	4.71	-0.099
$\beta_{zzz}\times10^{-30}$	-0.31	-1.40	-1.07	-1.15	-1.30	0.614	1.12	0.956
$\beta_{tot}\times10^{-27}$	0.05	6.98	8.23	6.03	6.86	7.64	7.84	5.96

Table S37: Second hyper-polarizabilities and major contributing tensors (*esu*) of the studied compounds.

**Compounds**	$\gamma_x\times10^{-31}$	$\gamma_y\times10^{-33}$	$\gamma_z\times10^{-35}$	$<\gamma>\times10^{-31}$
**ATTR1**	0.72	0.10	0.43	0.72
**ATTD2**	1.28	0.83	0.96	1.29
**ATTD3**	1.63	2.88	12.7	1.66
**ATTD4**	1.08	1.69	0.98	1.10
**ATTD5**	1.21	2.48	6.69	1.24
**ATTD6**	1.42	3.01	9.51	1.45
**ATTD7**	1.47	2.21	1.10	1.49
**ATTD8**	1.06	1.89	10.2	1.08

Table S38: The IUPAC names and abbreviations of investigated compounds

Compound	IUPAC name
**ATTD2**	(Z)-2-(2-((6-(7-(4-(4-(bis(4-methoxyphenyl)amino)phenyl)-2-methylthieno[3,4-b]thiophen-6-yl)-4,4,9,9-tetramethyl-4,9-dihydro-s-indaceno[1,2-b:5,6-b']dithiophen-2-yl)-2-methylthieno[3,4-b]thiophen-4-yl)methylene)-3-oxo-2,3-dihydro-1*H*-inden-1-ylidene)malononitrile
**ATTD3**	(Z)-2-(2-((6-(7-(4-(4-(bis(4-methoxyphenyl)amino)phenyl)-2-methylthieno[3,4-b]thiophen-6-yl)-4,4,9,9-tetramethyl-4,9-dihydro-s-indaceno[1,2-b:5,6-b']dithiophen-2-yl)-2-methylthieno[3,4-b]thiophen-4-yl)methylene)-6,7-dinitro-3-oxo-2,3-dihydro-1*H*-benzo[b]cyclopenta[d]thiophen-1-ylidene)malononitrile
**ATTD4**	(Z)-2-(2-((6-(7-(4-(4-(bis(4-methoxyphenyl)amino)phenyl)-2-methylthieno[3,4-b]thiophen-6-yl)-4,4,9,9-tetramethyl-4,9-dihydro-s-indaceno[1,2-b:5,6-b']dithiophen-2-yl)-2-methylthieno[3,4-b]thiophen-4-yl)methylene)-6,7-dichloro-3-oxo-2,3-dihydro-1*H*-benzo[b]cyclopenta[d]thiophen-1-ylidene)malononitrile
**ATTD5**	(Z)-2-(2-((6-(7-(4-(4-(bis(4-methoxyphenyl)amino)phenyl)-2-methylthieno[3,4-b]thiophen-6-yl)-4,4,9,9-tetramethyl-4,9-dihydro-s-indaceno[1,2-b:5,6-b']dithiophen-2-yl)-2-methylthieno[3,4-b]thiophen-4-yl)methylene)-3-oxo-6,7-bis(trifluoromethyl)-2,3-dihydro-1*H*-benzo[b]cyclopenta[d]thiophen-1-ylidene)malononitrile
**ATTD6**	(Z)-2-((6-(7-(4-(4-(bis(4-methoxyphenyl)amino)phenyl)-2-methylthieno[3,4-b]thiophen-6-yl)-4,4,9,9-tetramethyl-4,9-dihydro-s-indaceno[1,2-b:5,6-b']dithiophen-2-yl)-2-methylthieno[3,4-b]thiophen-4-yl)methylene)-1-(dicyanomethylene)-3-oxo-2,3-dihydro-1*H*-benzo[b]cyclopenta[d]thiophene-6,7-disulfonic acid
**ATTD7**	Z)-2-((6-(7-(4-(4-(bis(4-methoxyphenyl)amino)phenyl)-2-methylthieno[3,4-b]thiophen-6-yl)-4,4,9,9-tetramethyl-4,9-dihydro-s-indaceno[1,2-b:5,6-b']dithiophen-2-yl)-2-methylthieno[3,4-b]thiophen-4-yl)methylene)-1-(dicyanomethylene)-3-oxo-2,3-dihydro-1*H*-benzo[b]cyclopenta[d]thiophene-6,7-dicarbonitrile
**ATTD8**	dimethyl(Z)-2-((6-(7-(4-(4-(bis(4-methoxyphenyl)amino)phenyl)-2-methylthieno[3,4-b]thiophen-6-yl)-4,4,9,9-tetramethyl-4,9-dihydro-s-indaceno[1,2-b:5,6-b']dithiophen-2-yl)-2-methylthieno[3,4-b]thiophen-4-yl)methylene)-1-(dicyanomethylene)-3-oxo-2,3-dihydro-1*H*-benzo[b]cyclopenta[d]thiophene-6,7-dicarboxylate

Table S39: Representative values of NBOs study for the entitled compounds (**ATTR1** and **ATTD2-ATTD8**).

Compounds	Donor(*i*)	Type	Acceptor(*j*)	Type	*E*(2) [*kcal/mol*]	*E*(*j*)-*E*(*i*) [*a.u*]	*F*(*i,j*) [*a.u*]
	C23-C31	π	C33-C54	π*	33.74	0.3	0.091
	C67-N68	π	C69-N70	π*	0.78	0.48	0.017
	C33-H53	σ	S26-C31	σ*	10.71	0.72	0.078
**ATTR1**	C67-N68	σ	C56-C57	σ*	0.5	1.65	0.026
	S26	LP(2)	C24-C25	π*	28.61	0.25	0.078

	O52	LP(2)	C43-C47	σ*	20.79	0.76	0.114
**ATTD2**	C27-C28	π	C33-C34	π*	34.84	0.3	0.092
	C107-C108	π	C97-C99	π*	0.76	0.3	0.014
	C33-H35	σ	C27-S30	σ*	10.85	0.72	0.079
	C73-S83	σ	C28-C29	σ*	0.51	1.2	0.022
	O118	LP(2)	C102-C104	π*	32.47	0.36	0.103
	O51	LP(2)	C42-C46	σ*	20.86	0.76	0.114
**ATTD3**	C27-C28	π	C33-C34	π*	36.23	0.3	0.093
	C101-C102	π	C91-C93	π*	0.76	0.3	0.014
	C33-H35	σ	C27-S30	σ*	10.77	0.72	0.079
	C69-S79	σ	C28-C29	σ*	0.51	1.2	0.022
	O112	LP(2)	C96-C98	π*	32.44	0.36	0.103
	O47	LP(2)	C41-C42	σ*	22.65	0.74	0.117
**ATTD4**	C27-C28	π	C33-C34	π*	34.1	0.3	0.091
	C45-N46	π	C43-N44	π*	0.77	0.48	0.017
	C33-H35	σ	C27-S30	σ*	10.58	0.72	0.078
	S17-C20	σ	C10-C21	σ*	0.51	1.11	0.021
	O111	LP(2)	C104-C108	π*	32.59	0.36	0.103
	O47	LP(2)	C41-C42	σ*	22.21	0.74	0.116
**ATTD5**	C27-C28	π	C33-C34	π*	35.22	0.3	0.093
	C45-N46	π	C43-N44	π*	0.78	0.48	0.017
	C33-H35	σ	C27-S30	σ*	10.72	0.72	0.078
	C45-N46	σ	C36-C37	σ*	0.5	1.64	0.026
	O112	LP(2)	C96-C98	π*	32.51	0.36	0.103
	O47	LP(2)	C41-C42	σ*	22.37	0.74	0.117
**ATTD6**	C27-C28	π	C33-C34	π*	36.16	0.3	0.093
	C101-C102	π	C91-C93	π*	0.77	0.3	0.014
	C33-H35	σ	C27-S30	σ*	10.74	0.72	0.079
	C45-N46	σ	C36-C37	σ*	0.5	1.64	0.026
	O112	LP(2)	C96-C98	π*	32.44	0.36	0.103
	O131	LP(1)	S128-O129	σ*	2.68	1.02	0.048
**ATTD7**	C27-C28	π	C33-C34	π*	35.93	0.3	0.093
	C45-N46	π	C43-N44	π*	0.76	0.47	0.017
	C33-H35	σ	C27-S30	σ*	10.72	0.72	0.078
	C69-S79	σ	C28-C29	σ*	0.51	1.2	0.022
	N90	LP(1)	C83-C87	π*	32.73	0.29	0.091
	O47	LP(2)	C41-C42	σ*	22.62	0.74	0.117
**ATTD8**	C27-C28	π	C33-C34	π*	34.19	0.3	0.092
	C101-C102	π	C91-C93	π*	0.72	0.3	0.013
	C33-H35	σ	C27-S30	σ*	10.61	0.72	0.078
	C123-H124	σ	125-C128	σ*	0.5	0.96	0.02
	O130	LP(2)	C128-O129	π*	50.66	0.37	0.124
	O132	LP(2)	C131-O133	σ*	33.13	0.68	0.135

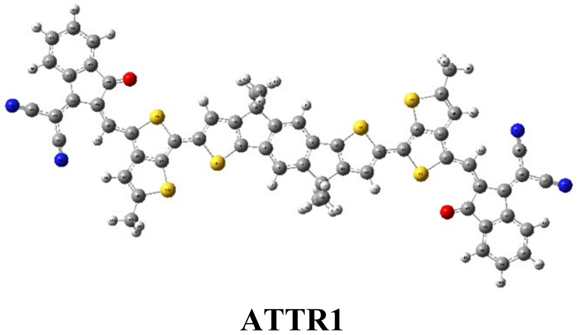

ATTR1

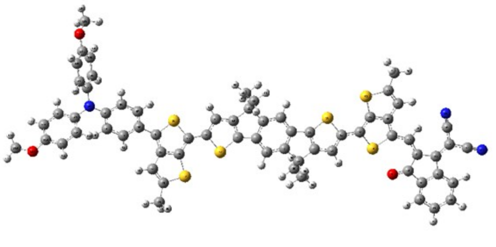

ATTD2

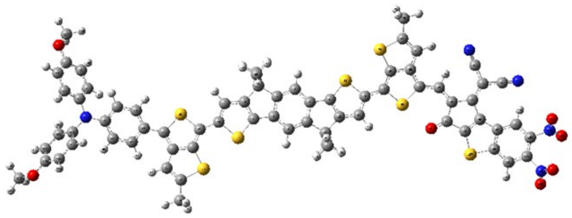

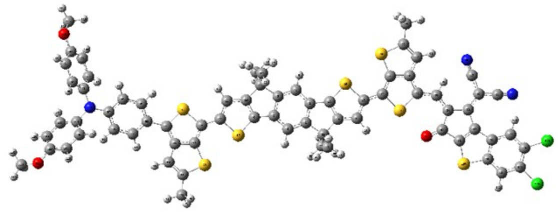

ATTD3 **ATTD4**

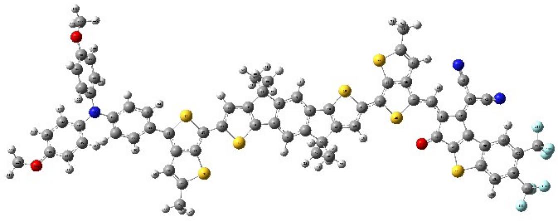

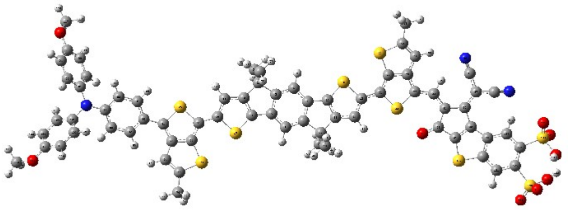

ATTD5 **ATTD6**

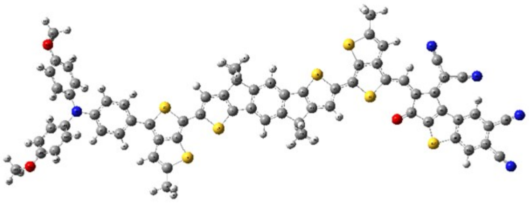

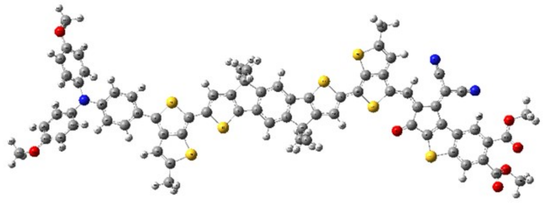

ATTD7 **ATTD8**

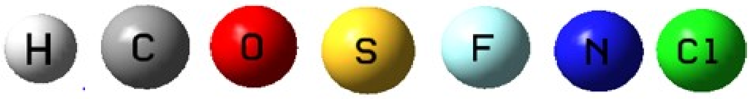

Figure S1: Optimized structures of reference compound (**ATTR1**) and designed derivatives (**ATTD2-ATTD8**).

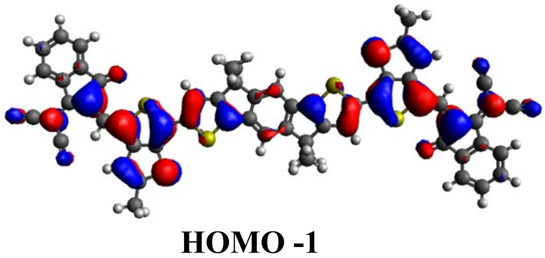

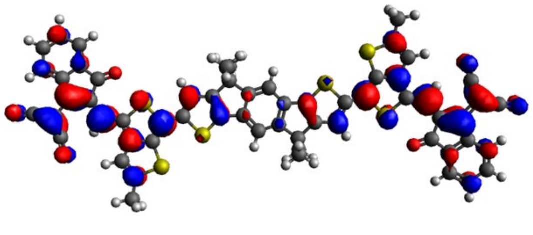

HOMO -1 **LUMO +1**

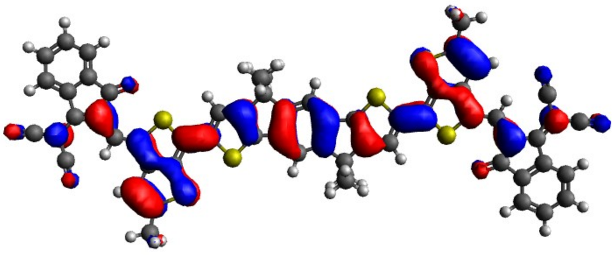

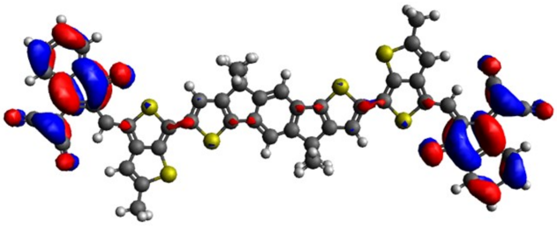

HOMO -2 **LUMO +2**

ATTR1

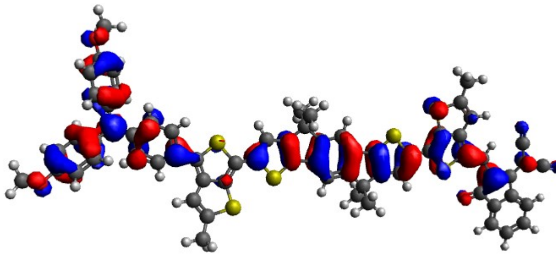

HOMO -1

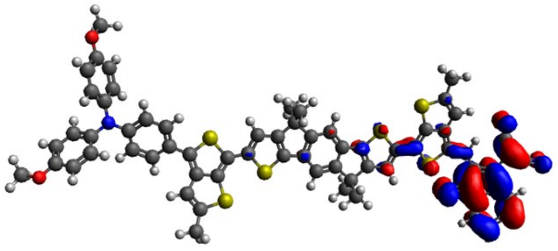

LUMO +1

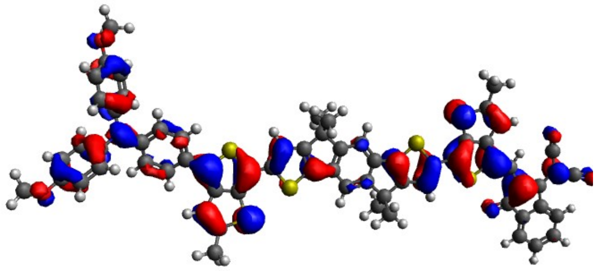

HOMO -2

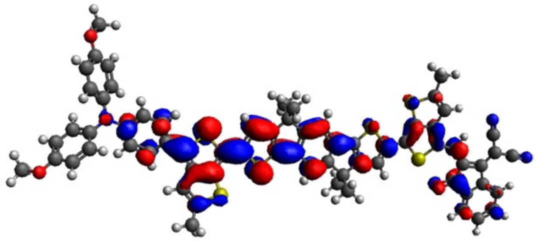

LUMO +2

ATTD2

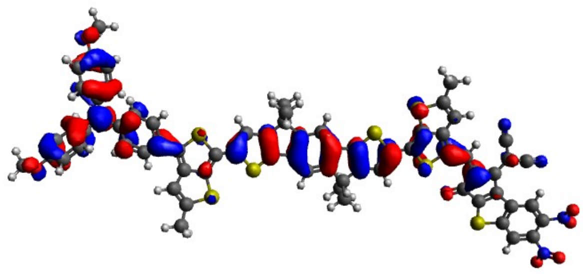

HOMO -1

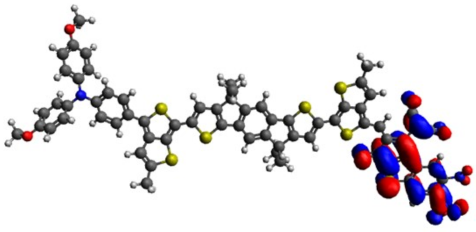

LUMO +1

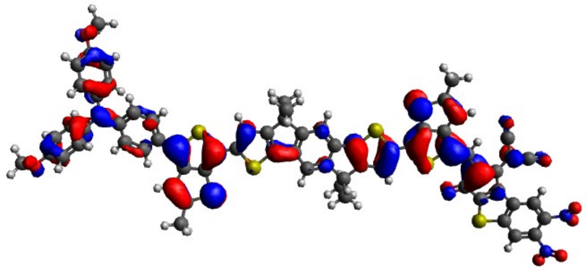

HOMO -2

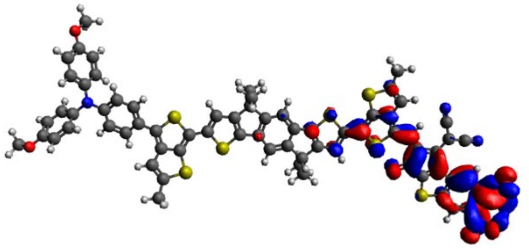

LUMO +2

ATTD3

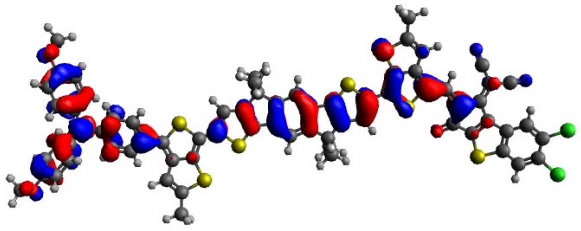

HOMO -1

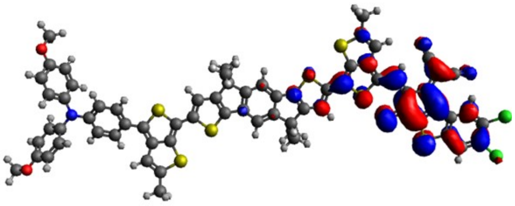

LUMO +1

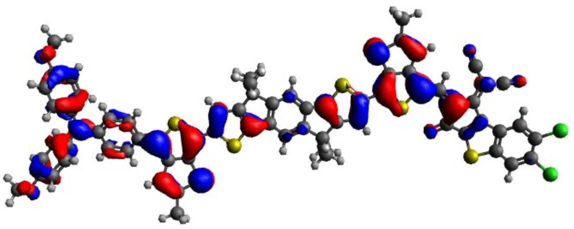

HOMO -2

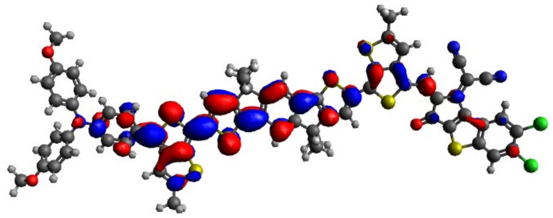

LUMO +2

ATTD4

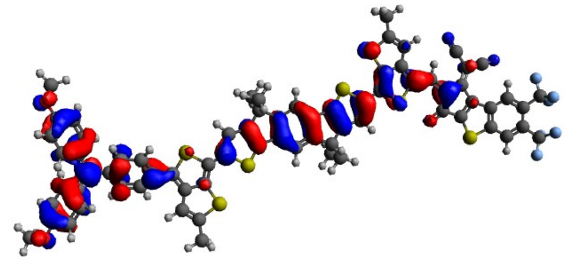

HOMO -1

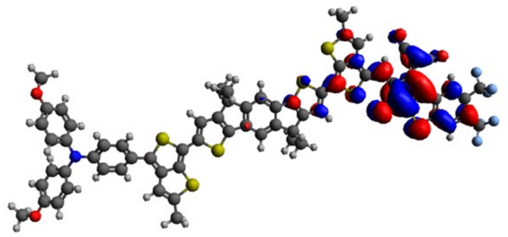

LUMO +1

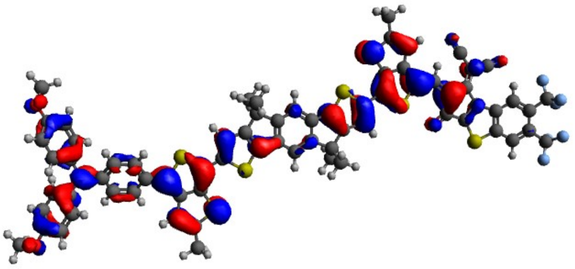

HOMO -2

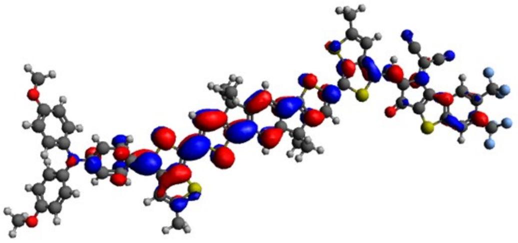

LUMO +2

ATTD5

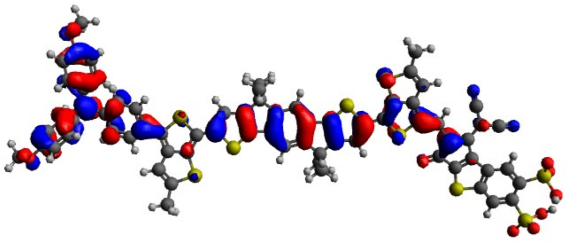

HOMO -1

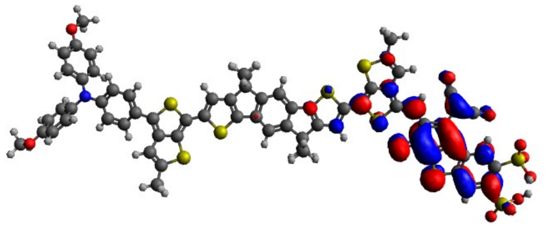

LUMO +1

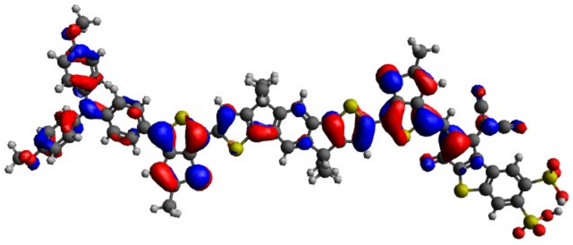

HOMO -2

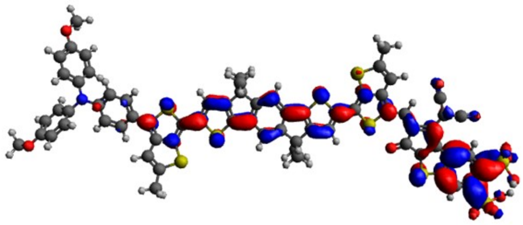

LUMO +2

ATTD6

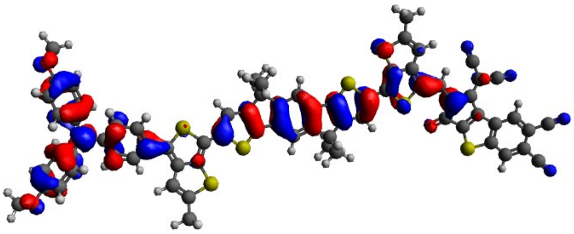

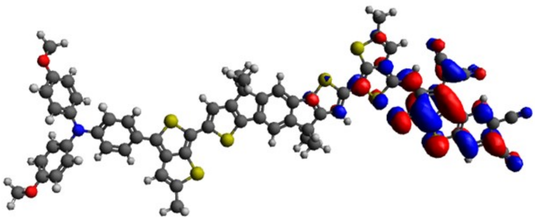

HOMO -1 **LUMO +1**

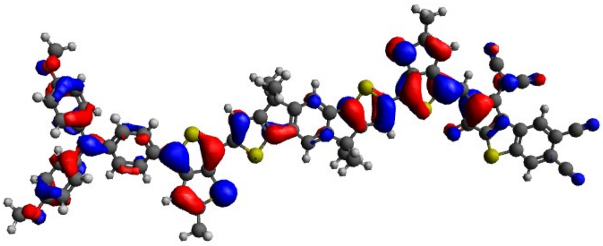

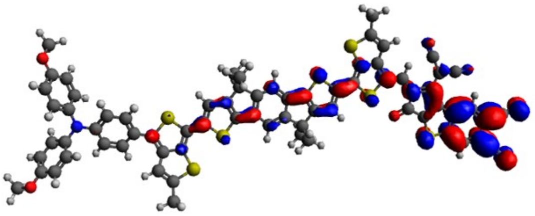

HOMO -2 **LUMO +2**

ATTD7

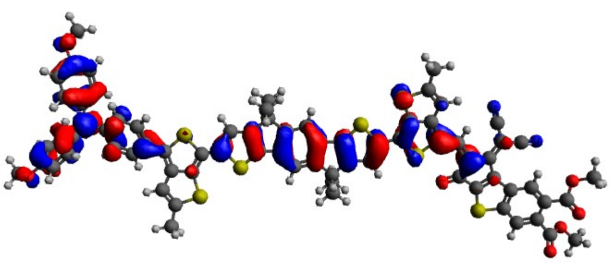

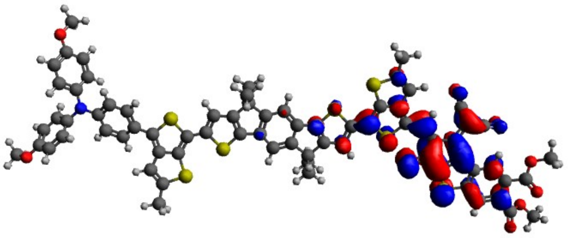

HOMO -1 **LUMO +1**

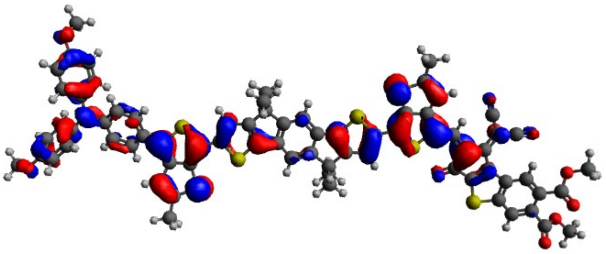

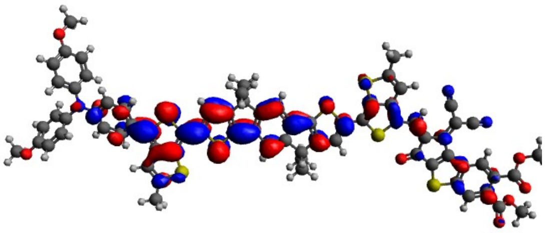

HOMO -2 **LUMO +2**

ATTD8

Figure S2: The pictograph of HOMO-1, LUMO+1, HOMO-2 and LUMO+2 of tailored compounds (**ATTR1** and **ATTD2-ATTD8**).

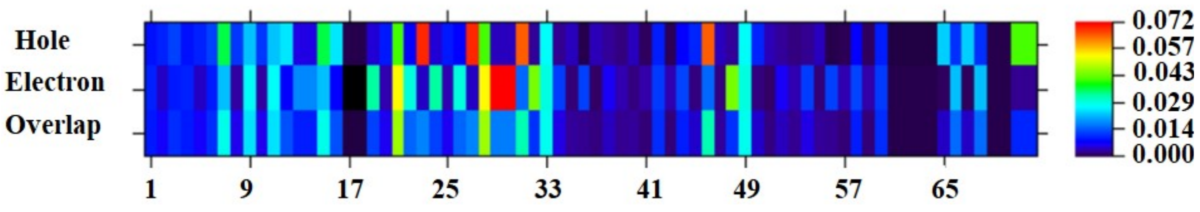

ATTR1

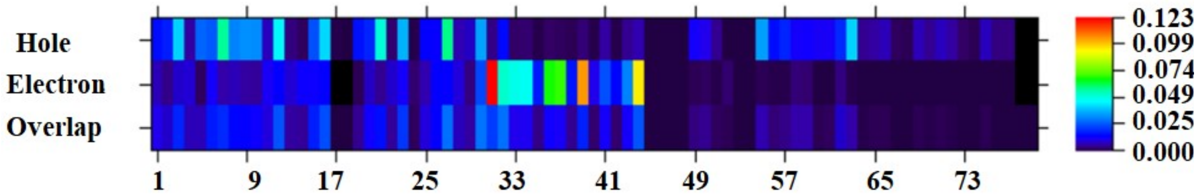

ATTD2

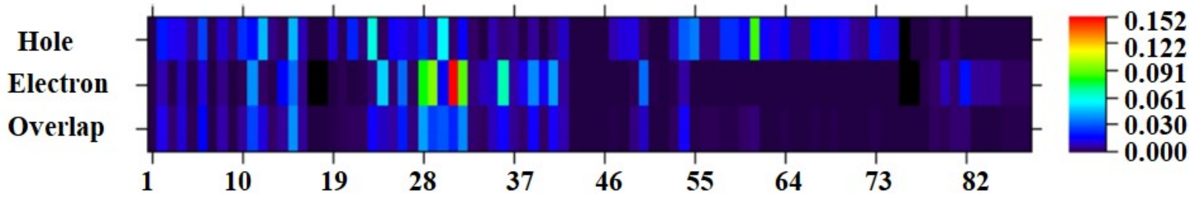

ATTD3

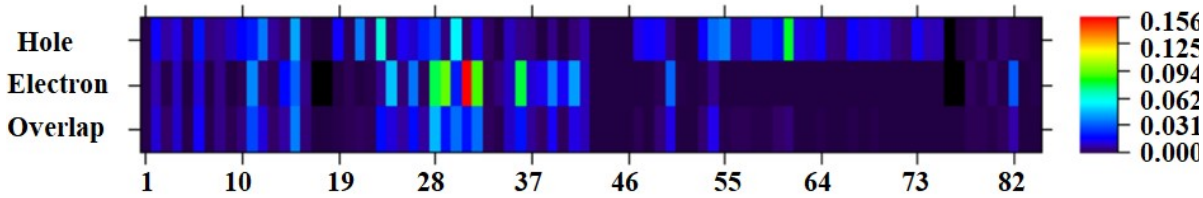

ATTD4

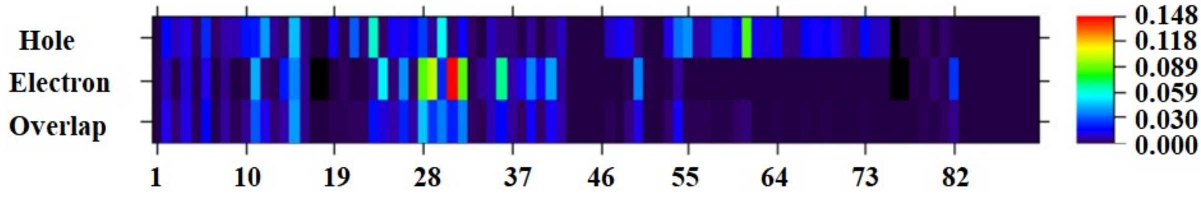

ATTD5

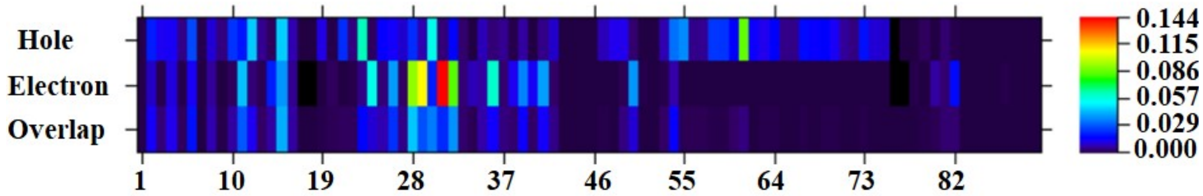

ATTD6

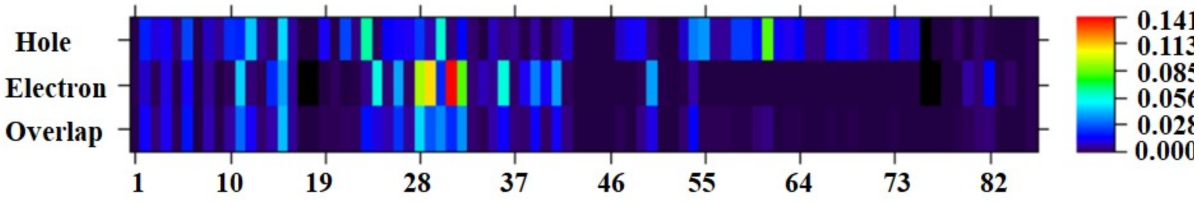

ATTD7

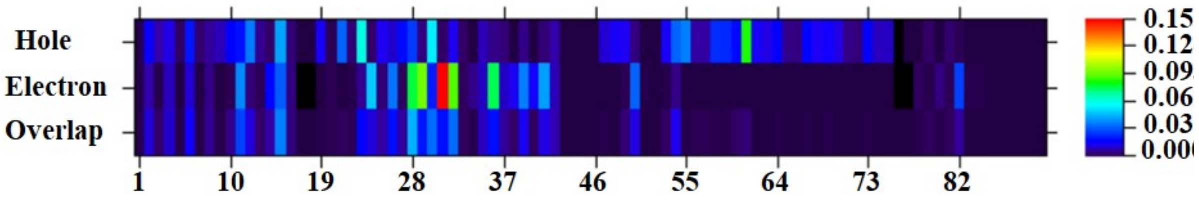

ATTD8

Figure S3. Pictorial representation of hole-electron transport investigation.

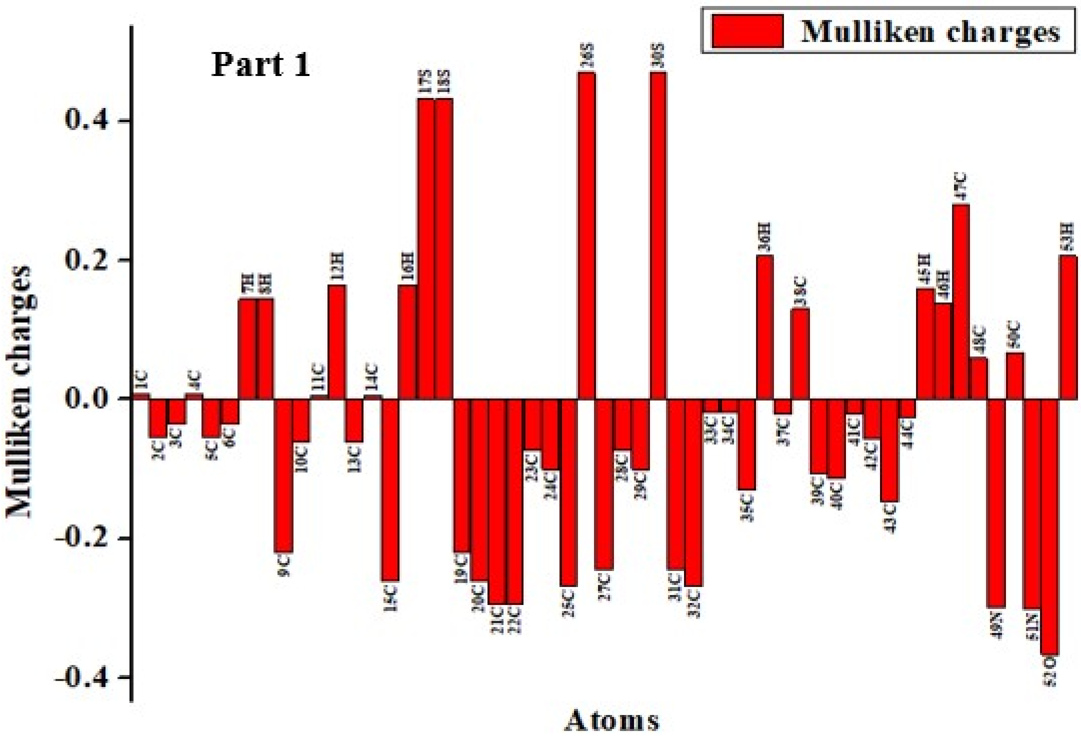

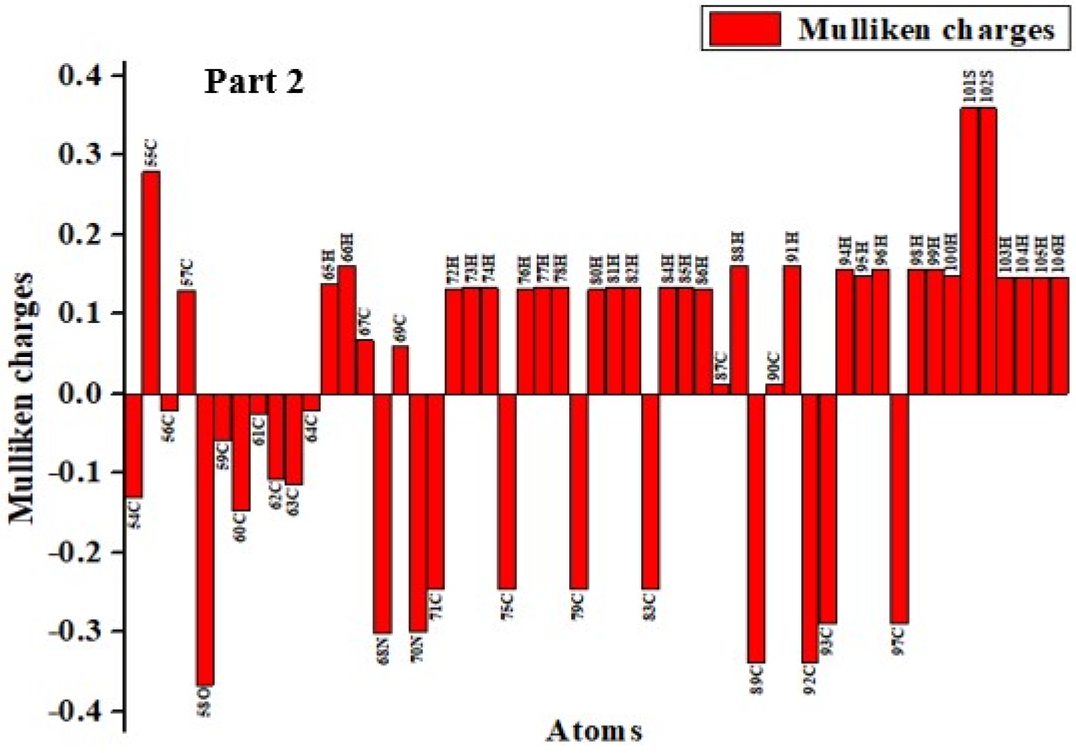
Mulliken charges
Part 2
Mulliken charges
Atoms

ATTR1

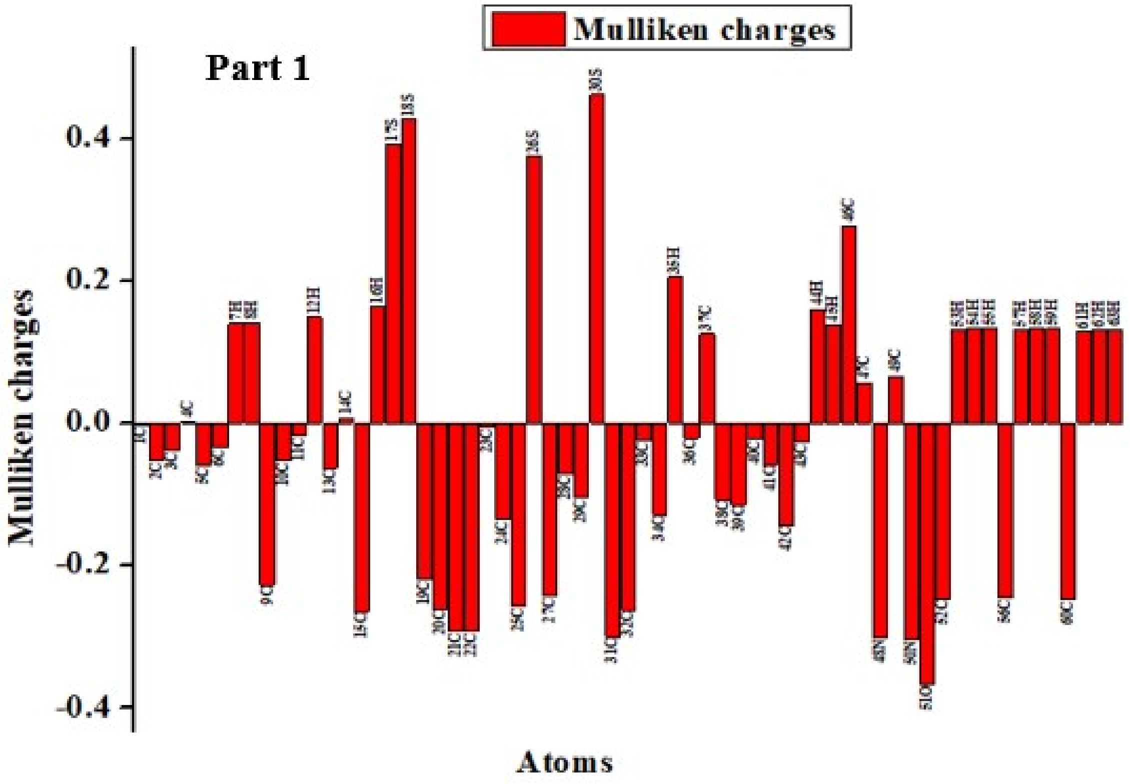
Mulliken charges
Part 1
Mulliken charges
Atoms

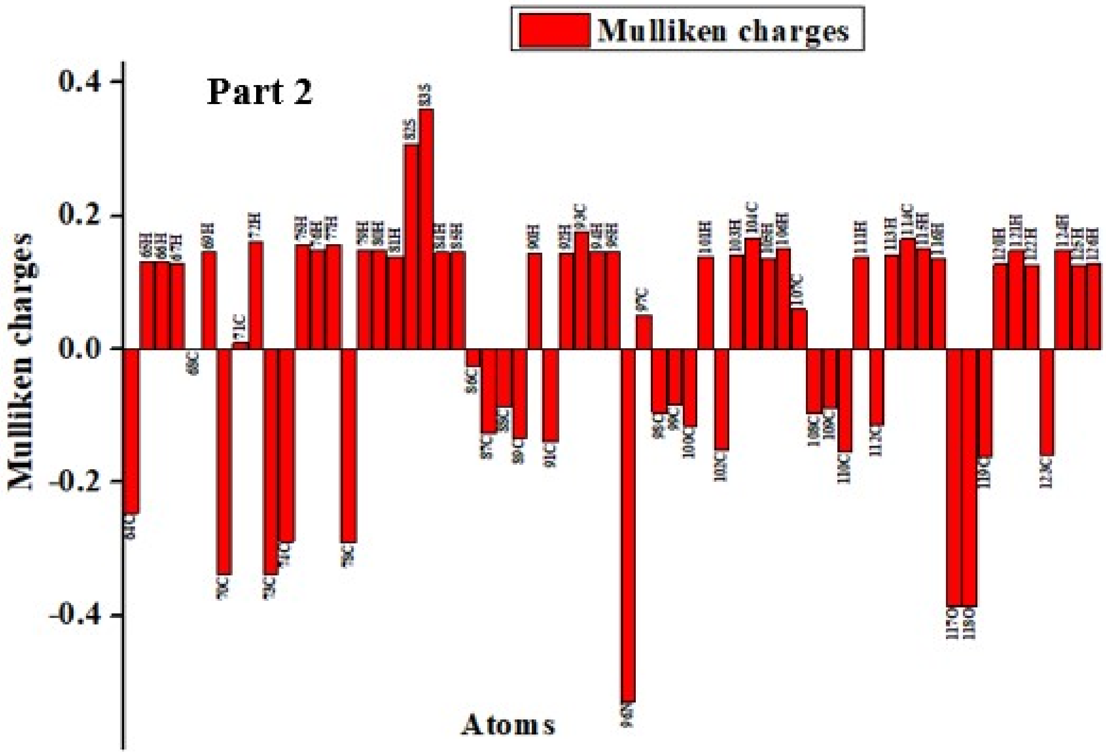
Mulliken charges
Part 2
Mulliken charges
0.4
0.2
0.0
-0.2
-0.4
Atoms

ATTD2

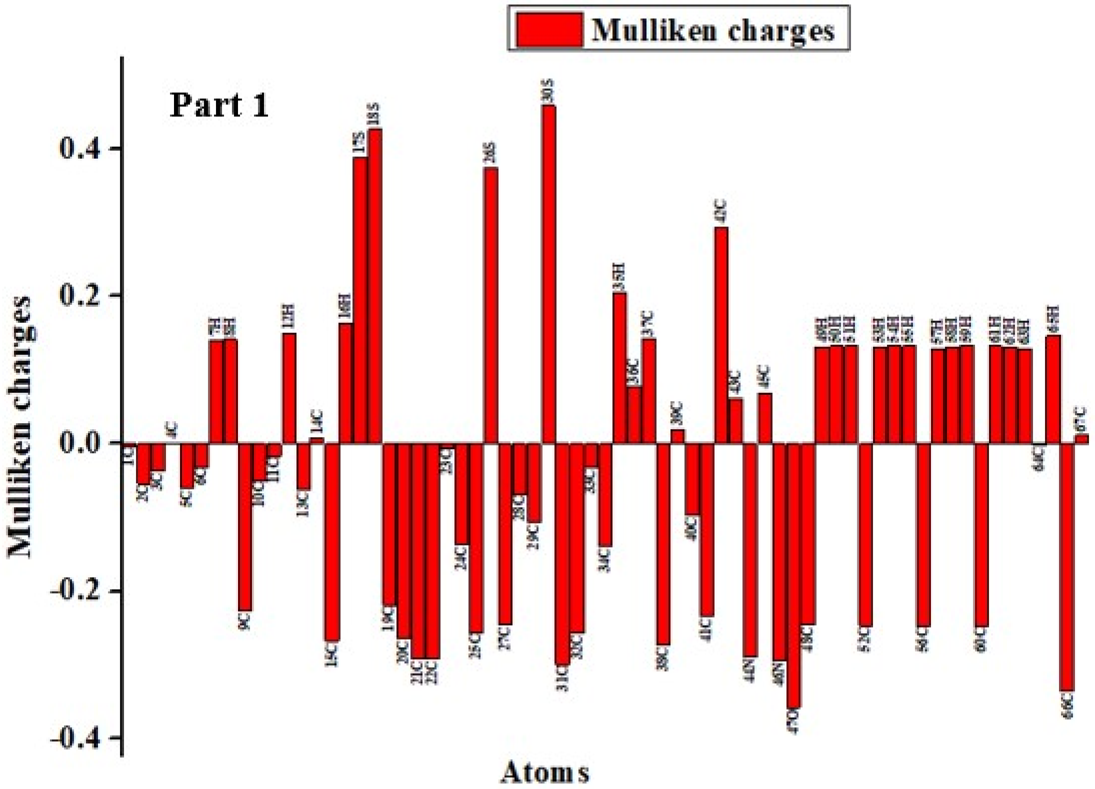
Mulliken charges
Part 1
Mulliken charges
0.4
0.2
0.0
-0.2
-0.4
Atoms

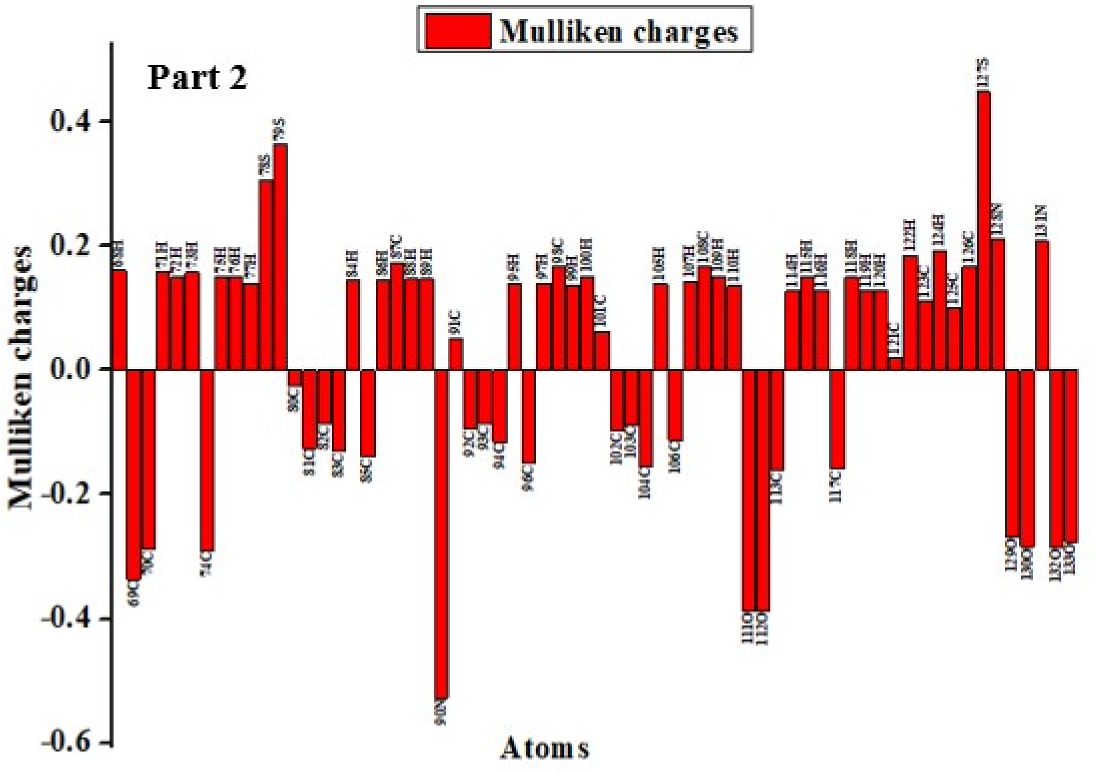
Mulliken charges
Part 2
Mulliken charges
Atoms
0.4
0.2
0.0
-0.2
-0.4
-0.6

ATTD3

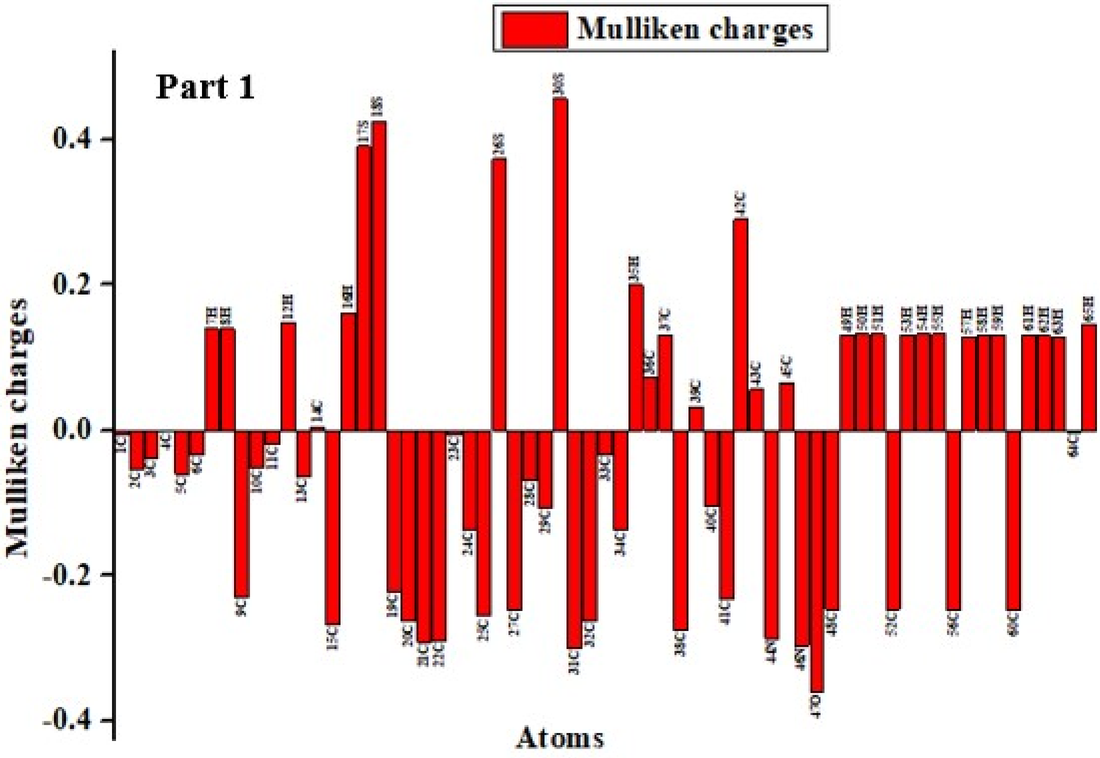
Mulliken charges
Part 1
Mulliken charges
Atoms
0.4
0.2
0.0
-0.2
-0.4

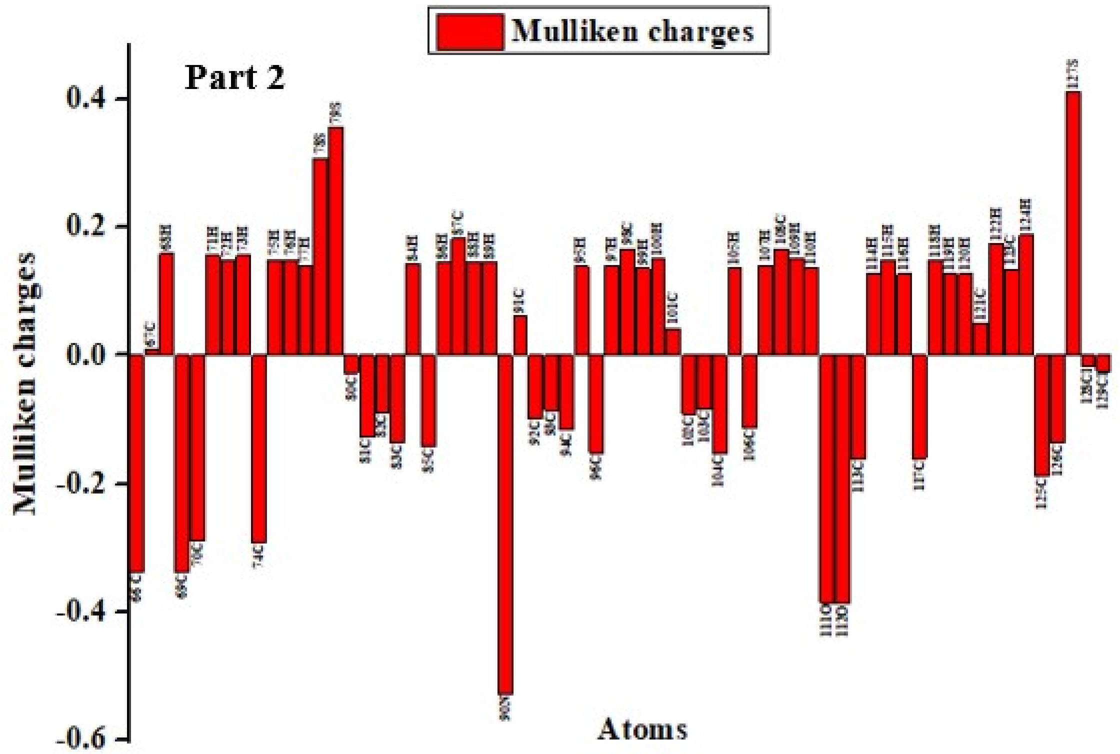
Mulliken charges
Part 2
Mulliken charges
Atoms

ATTD4

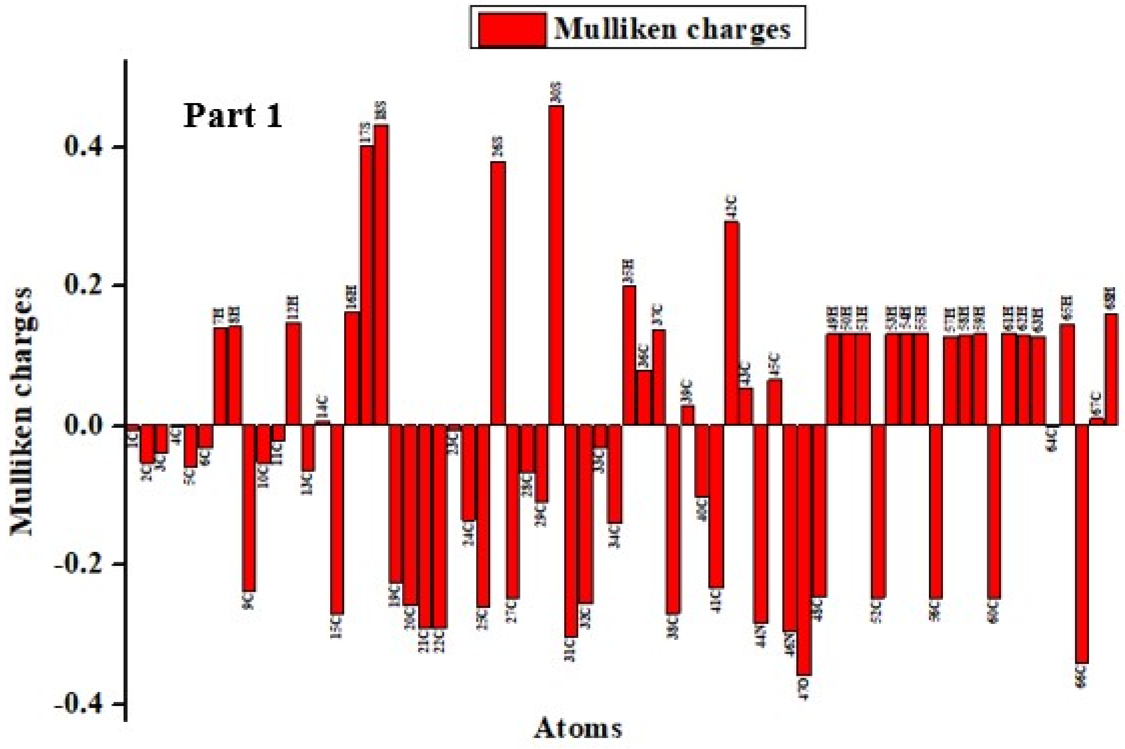
Mulliken charges
Part 1
Mulliken charges
Atoms

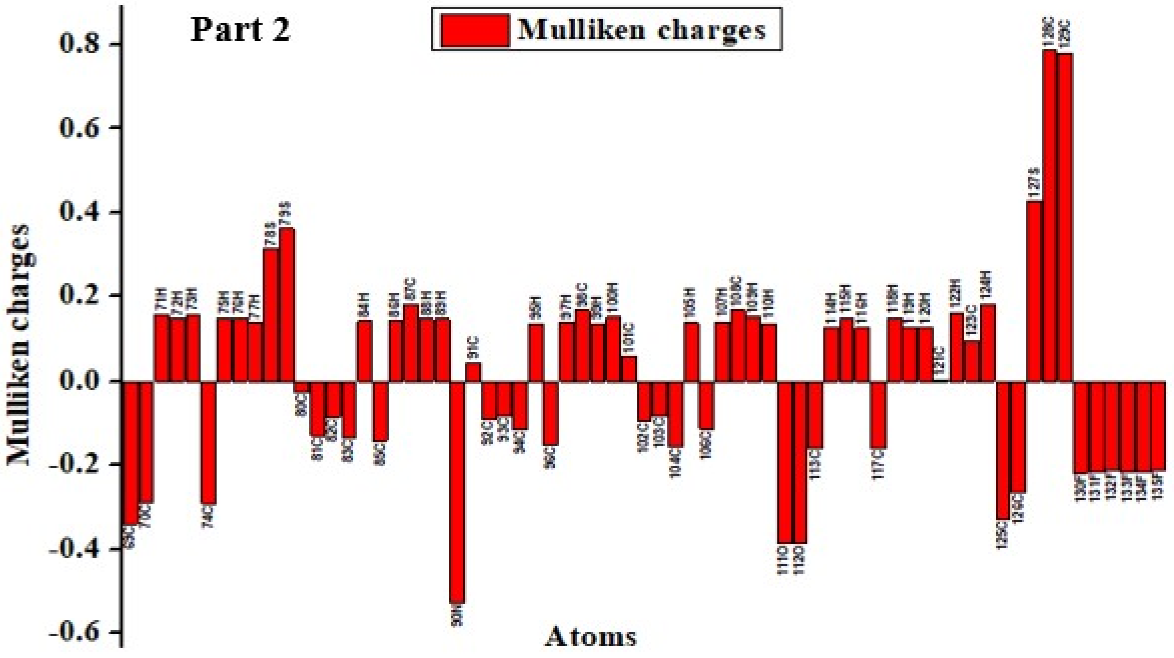
Part 2
Mulliken charges
Mulliken charges
Atoms
0.8
0.6
0.4
0.2
0.0
-0.2
-0.4
-0.6

ATTD5

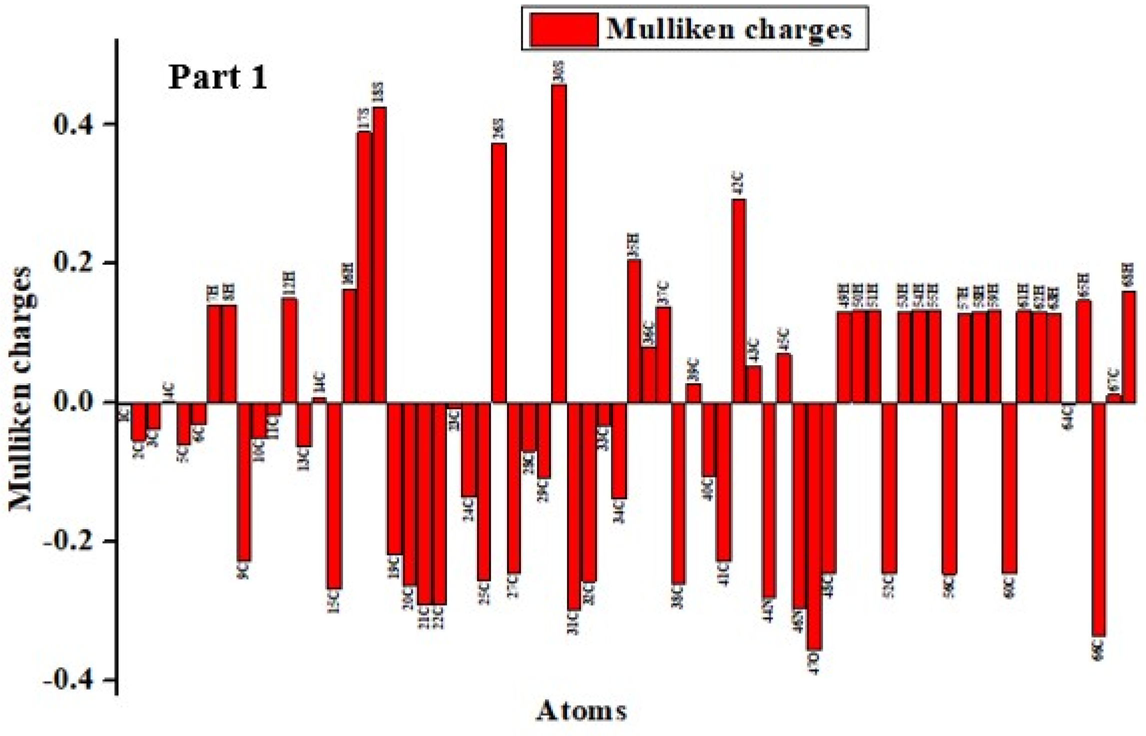
Part 1
Mulliken charges
Mulliken charges
Atoms
0.4
0.2
0.0
-0.2
-0.4

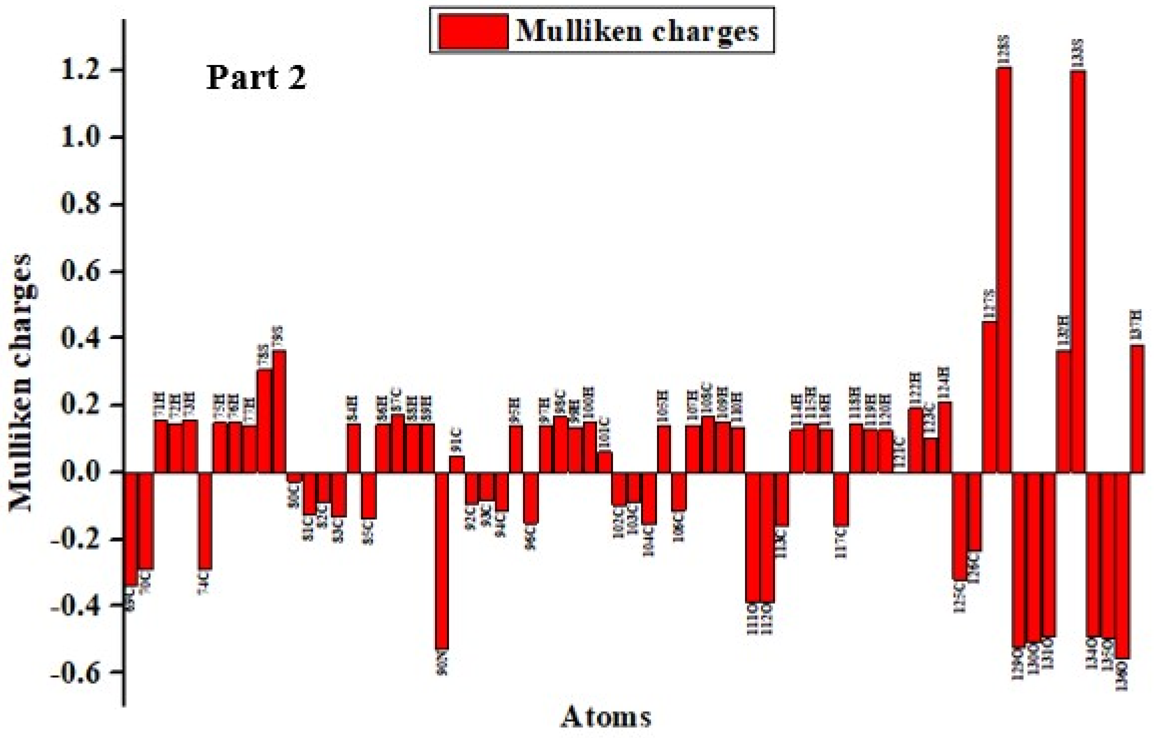
Mulliken charges
Part 2
Mulliken charges
Atoms

ATTD6

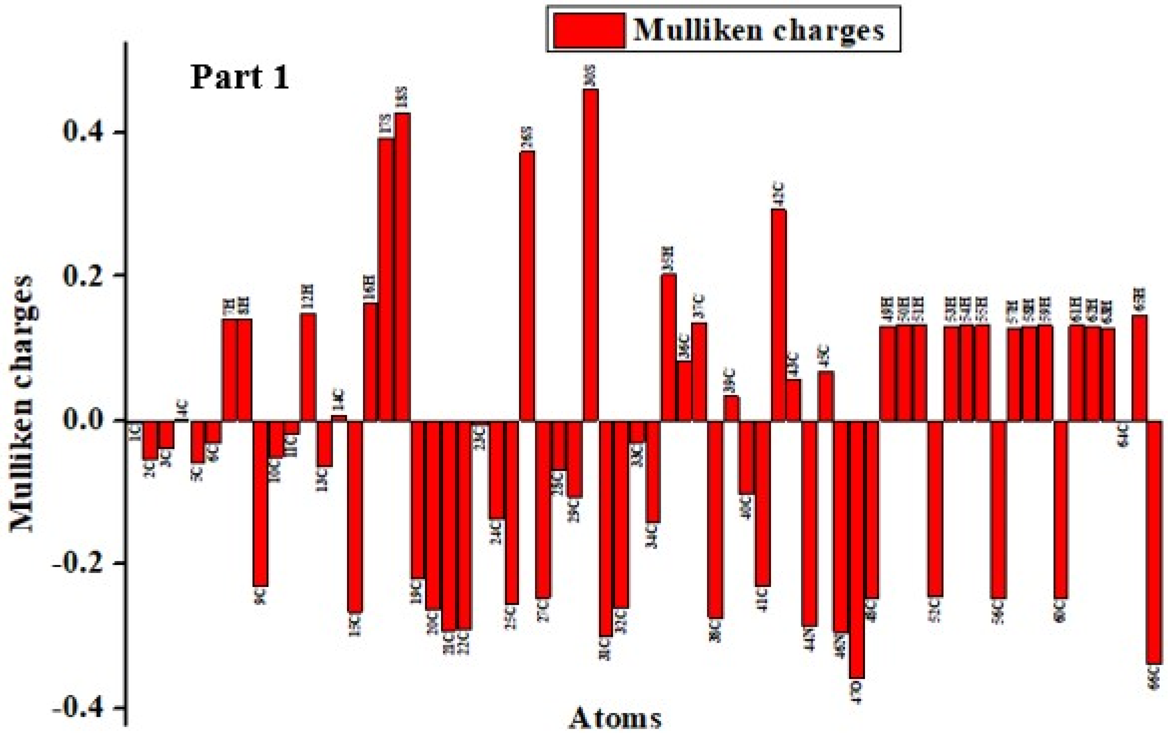
Mulliken charges
Part 1
Mulliken charges
Atoms

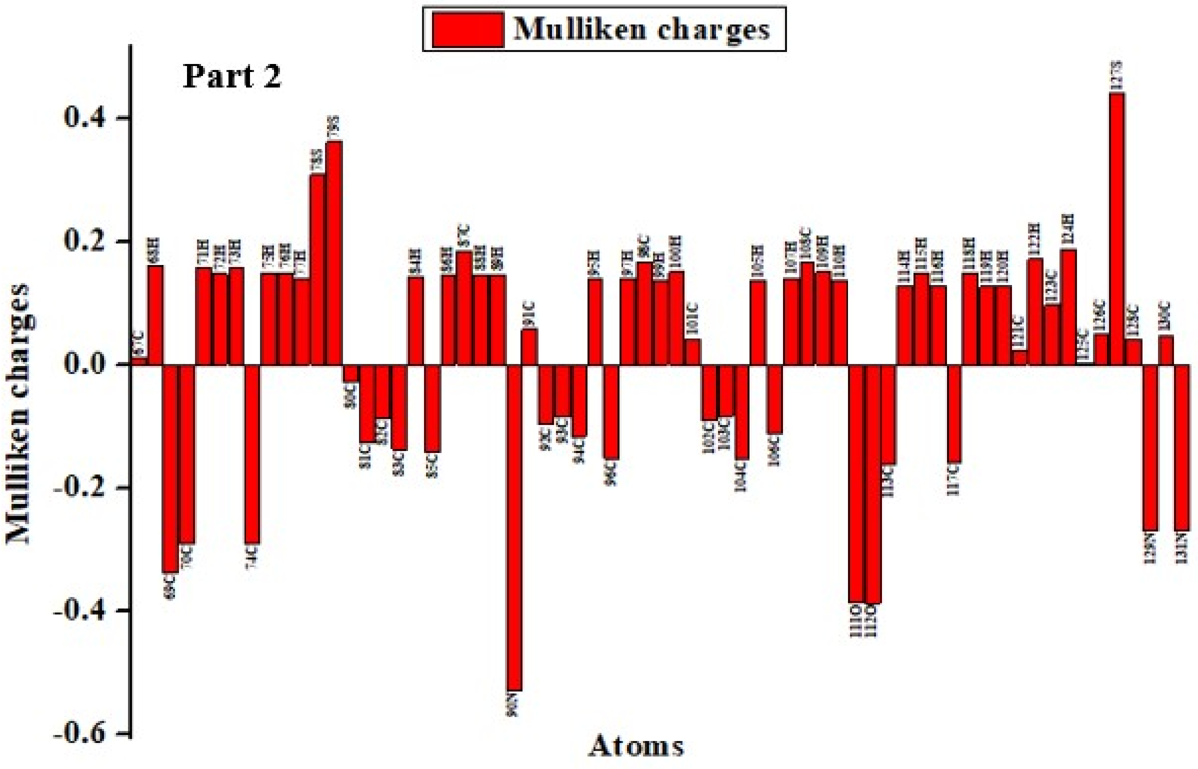
Mulliken charges
Part 2
Mulliken charges
Atoms

ATTD7

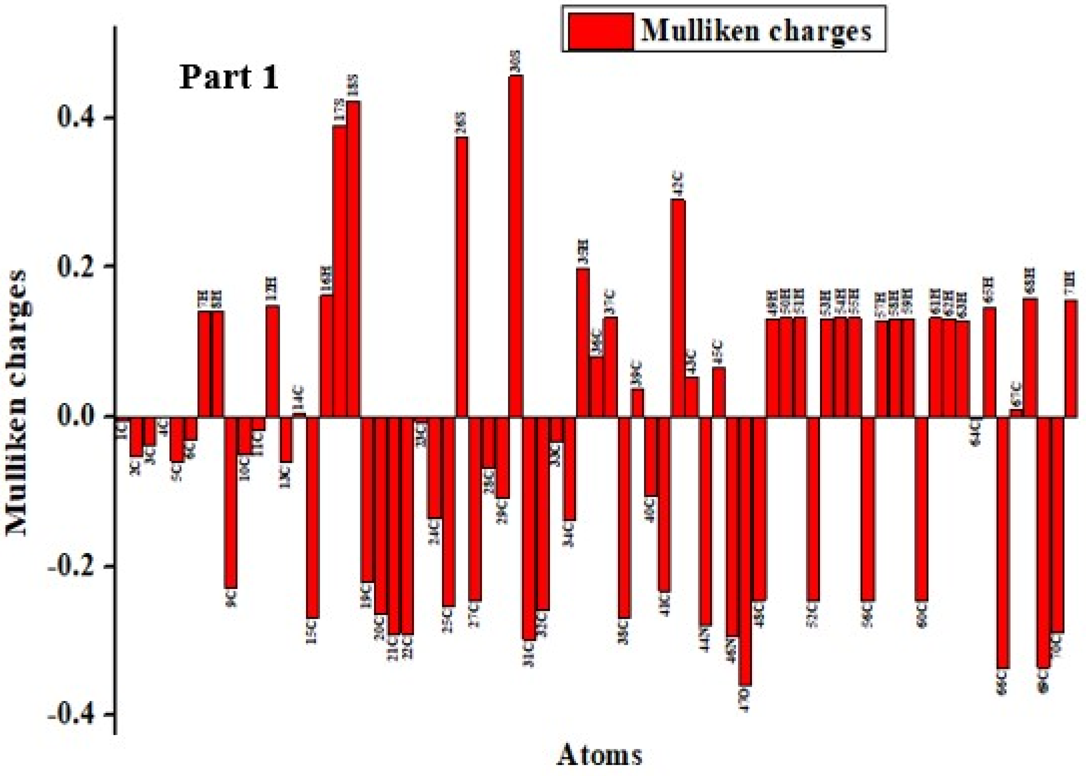
Mulliken charges
Part 1
Mulliken charges
Atoms

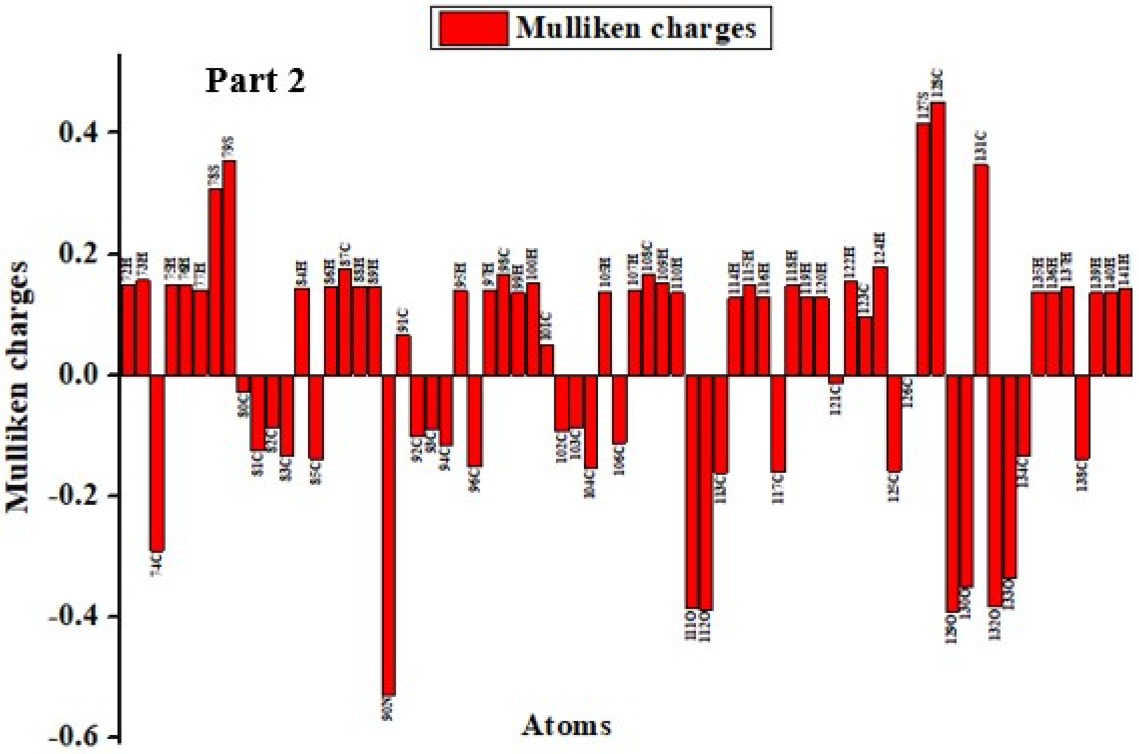

ATTD8

Figure S4: The graphs of Natural population analysis of **ATTR** and **ATTD2-ATTD8.**

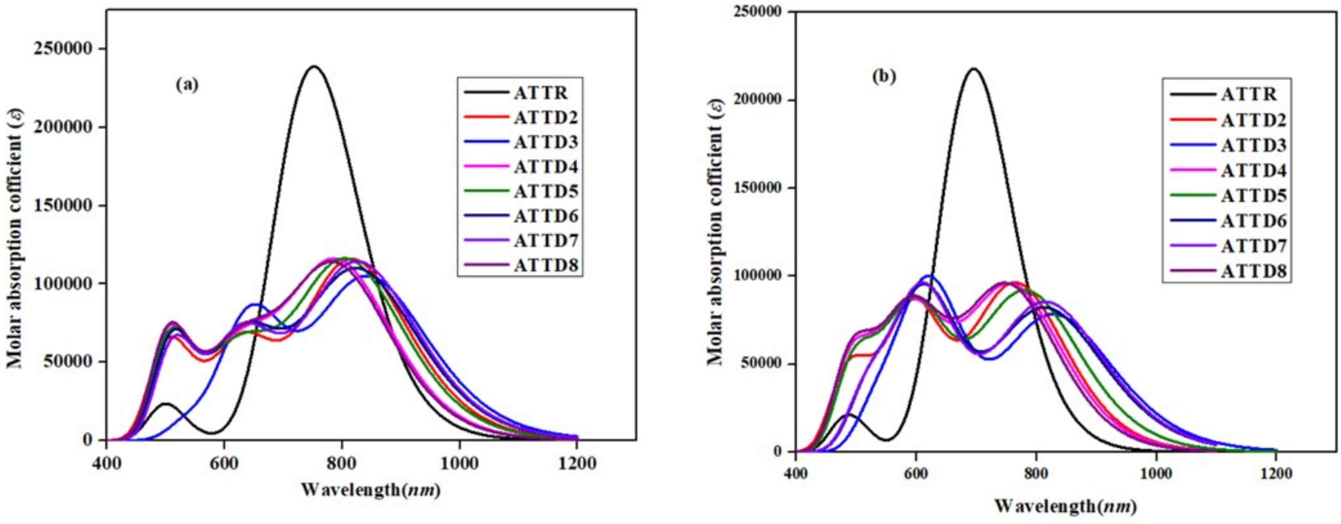

Figure S5: Absorption spectra of **ATTR** and **ATTD2-ATTD8 (a)** in chloroform and (b) in gaseous phase.

CH_3

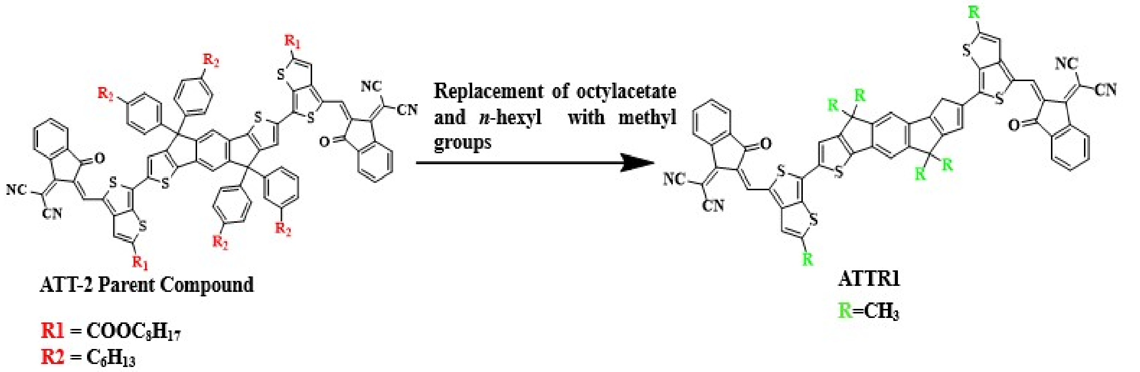

(a)

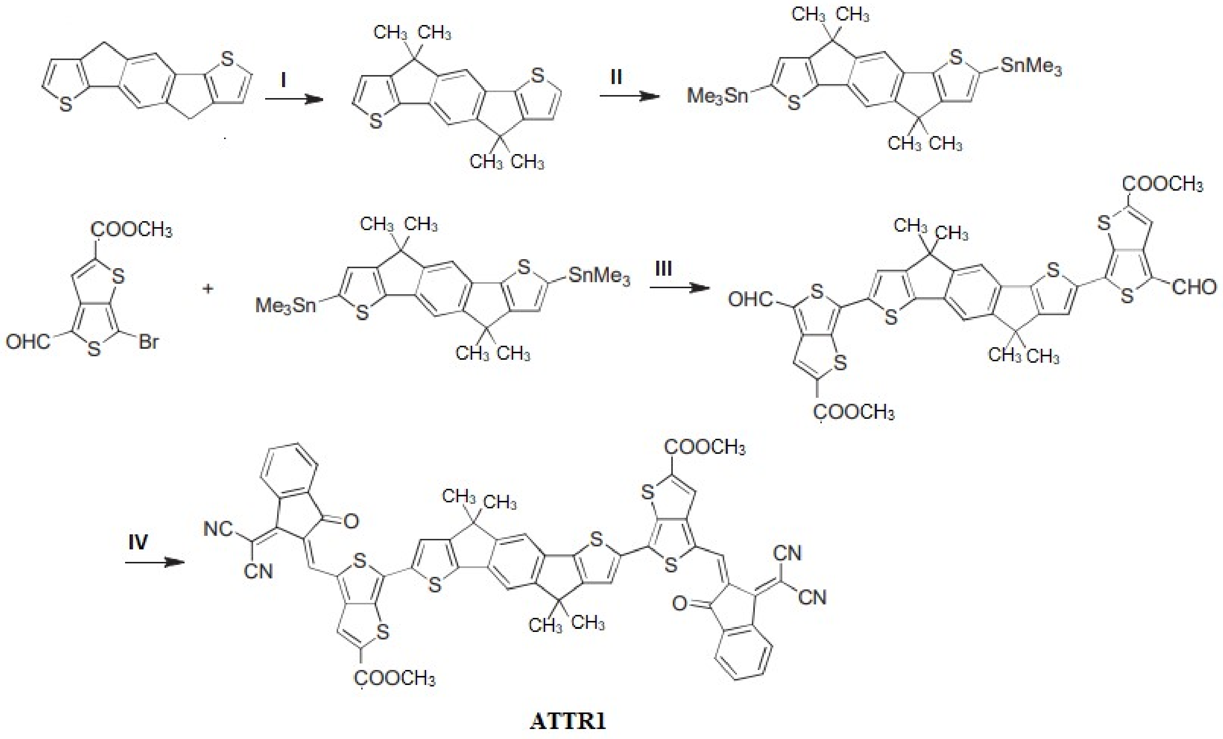

(b)

Figure S6: Side-chain variation of parent compound **ATT-2** into a reference **ATTR1 (a),** Synthesis of reference compound **ATTR1** reagents and conditions: I) NaOH/H_2O, CH_3-Br, 50 wt %, II) n-BuLi, $SnMe_3CL$,THF 95% III) Pd(PPh3)4, toluene, reflux; IV) 3-(dicyanomethylidene)indan-1-one, pyridine, chloroform, reflux **(b)**
